# Kidney fibrosis is mediated by GARP-restricted TGF-**β** activation in fibroblasts

**DOI:** 10.1172/jci.insight.203039

**Published:** 2026-09-17

**Authors:** Yintong Chen, Weiwei Xu, Jieli Yu, Pei Deng, Nianping Liu, Yinyin Li, Hui Zhou, Hong Zhou, Jianchuan Wang, Bo Zhao, Florian Winau, Fan Fan Hou, Yu Hu

**Affiliations:** 1 Division of Nephrology, Nanfang Hospital, Southern Medical University, State Key Laboratory of Multi-organ Injury Prevention and Treatment; National Clinical Research Center for Kidney and Urological Diseases, Guangdong Provincial Institute of Nephrology, Guangdong Provincial Key Laboratory of Renal Failure Research, Guangzhou, Guangdong, China.; 2 The First Affiliated Hospital of USTC, School of Basic Medical Sciences, Division of Life Sciences and Medicine, University of Science and Technology of China, Hefei, China.; 3 School of Pharmaceutical Sciences, Sun Yat-sen University, Guangzhou, Guangdong, China.; 4 Molecular Cancer Research Center, School of Medicine, Shenzhen Campus of Sun Yat-sen University, Shenzhen, Guangdong, China.; 5 Program in Cellular and Molecular Medicine, Boston Children’s Hospital, Department of Pediatrics, Harvard Medical School, Boston, Massachusetts, USA.; 6 Singapore Immunology Network (SIgN), Agency for Science, Technology and Research (A*STAR), Singapore.

**Keywords:** Nephrology, Public Health, Fibrosis

## Abstract

TGF-β is a central driver of kidney fibrosis, a common pathological hallmark of chronic kidney disease (CKD). Initiation of TGF-β signaling requires not only its synthesis but also the conversion of latent TGF-β to its bioactive form. However, the mechanisms governing TGF-β activation in the kidney and their contribution to kidney fibrosis remain poorly understood. Glycoprotein A repetitions predominant (GARP) anchors latent TGF-β on the cell surface and facilitates its bioactive release. Here, we show that GARP-mediated TGF-β activation promotes kidney fibrosis. GARP was upregulated in both human and mouse CKD kidneys, predominantly in fibroblasts, and was induced by TNF in an NF-κB–dependent fashion. In multiple mouse models of kidney fibrosis, either global or fibroblast-specific deletion of GARP markedly reduced fibrosis. Mechanistically, GARP enables sustained production of active TGF-β, thereby amplifying fibroblast stimulation. Deletion of GARP in kidney fibroblasts lowered active TGF-β levels and attenuated fibroblast activation, whereas GARP overexpression enhanced TGF-β signaling. Notably, tamoxifen-induced deletion of GARP after fibrosis onset attenuated kidney fibrosis. Together, our findings identify GARP-mediated release of active TGF-β as a critical step in sustaining fibroblast activation during kidney fibrosis and highlight GARP as a promising therapeutic target for CKD.

## Abstract 2

Fibroblast-expressed GARP promotes kidney fibrosis by enhancing TGF-β activation, identifying a potential therapeutic target to limit the progression of chronic kidney disease

## Graphical abstract

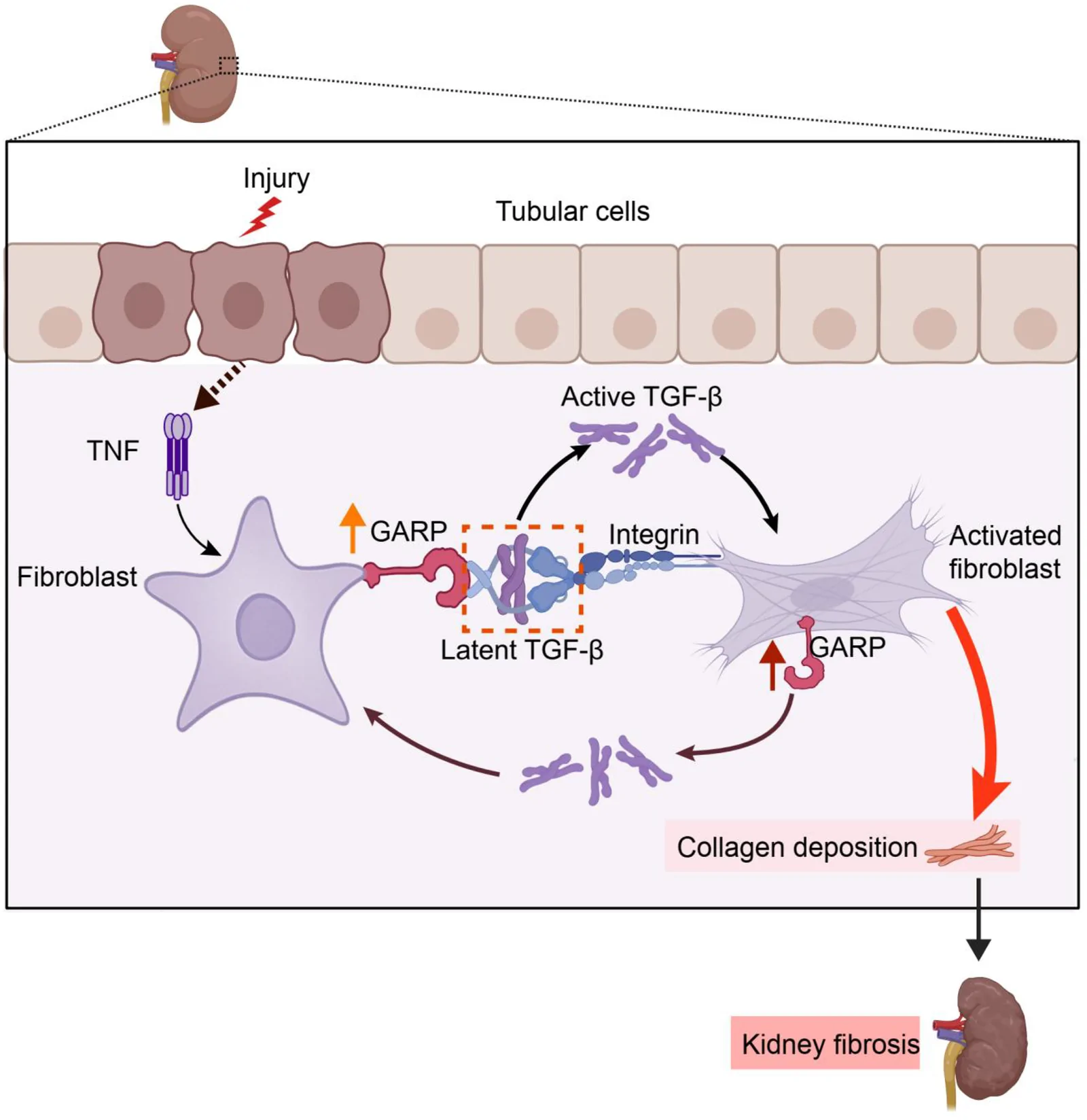

## Introduction

Chronic kidney disease (CKD) affects an estimated 800 million people worldwide, placing a substantial burden on patients and health care systems (1, 2). Progressive CKD frequently culminates in kidney fibrosis, characterized by excessive extracellular matrix (ECM) accumulation (3). The aberrant ECM deposition initiates a self-perpetuating scarring process that progressively impairs organ function (4). With no specific therapies currently available to halt or reverse fibrosis, there is an urgent need to identify new therapeutic targets (4).

Although multiple cell types contribute to kidney fibrosis, activation and differentiation of fibroblasts are central to disease progression due to their capacity to produce large quantities of ECM (5). Controlled activation of fibroblasts supports tissue repair after injury, whereas dysregulated and persistent activation drives excessive collagen synthesis and deposition, ultimately fueling fibrosis progression (6). Among the various stimuli for fibroblasts, TGF-β is arguably the most potent (7). TGF-β exerts diverse effects through both canonical Smad-dependent and noncanonical Smad-independent pathways, regulating distinct downstream targets in a cell-type- and context-dependent manner (8, 9). Even though blockade of TGF-β signaling efficiently reduces kidney fibrosis in animal models, complete inhibition is undesirable given its critical roles in vascular development and immune regulation, making direct therapeutic targeting of the TGF-β pathway in human CKD challenging (7, 10–12).

TGF-β is synthesized as an inactive latent complex (latent TGF-β) in which the latency-associated peptide (LAP) forms a loop around mature TGF-β, shielding the receptor-binding site (13, 14). Two main mechanisms mediate the transport and activation of latent TGF-β; latent TGF-β–binding proteins (LTBPs) direct its deposition in the ECM, whereas glycoprotein A repetitions predominant (GARP) anchors and concentrates latent TGF-β on the cell surface (15). Inhibiting components of the 2 systems has been shown to reduce fibrosis in the lung (16), heart (17), liver (18), kidney (19), and bone marrow (14), highlighting the therapeutic potential of targeting TGF-β activation at its source.

GARP (encoded by the *Lrrc32* gene) is a type I transmembrane protein that presents latent TGF-β on the surface of the cell (20). The GARP–latent TGF-β complex is activated through integrin αvβ6- or αvβ8-dependent mechanisms (21, 22), thereby releasing mature TGF-β. The function of GARP has been most extensively characterized in immune cells, particularly regulatory T lymphocytes (Tregs), where it mediates TGF-β activation and supports immunoregulatory processes such as oral tolerance and tumor immune evasion (22–25). Notably, autoimmune-associated variants at the 11q13.5 locus disrupt a Treg-specific, NF-κB–responsive enhancer that drives *LRRC32*/GARP expression, leading to impaired Treg suppressive function (26). Beyond the immune system, we recently showed that GARP is abundantly expressed on hepatic stellate cells and promotes liver fibrosis by facilitating TGF-β activation (18). However, the expression pattern and functional role of GARP in the kidney have not been defined.

## Results

### Upregulation of GARP in human and mouse fibrotic kidneys.

To investigate the potential role of GARP-mediated TGF-β activation in kidney fibrosis, we first assessed its expression by analyzing publicly available single-cell RNA sequencing (scRNA-seq) data from murine fibrotic kidneys (GSE190887) (27). At the whole-kidney sample level, analysis of unilateral ureteral obstruction (UUO) kidneys across multiple time points revealed that *Lrrc32* expression progressively increased along with fibrosis progression (Figure 1A). To validate these scRNA-seq findings, we next examined GARP expression by quantitative real-time PCR (RT-PCR) in 3 established rodent models of kidney fibrosis: UUO, unilateral renal ischemia-reperfusion injury (UIRI), and 5/6 nephrectomy (5/6Nx). In the UUO mouse model, renal *Lrrc32* mRNA levels were upregulated by day 7 and further increased by day 14 (Figure 1B) and correlated positively with fibrosis markers *Col1a1* and *Acta2* (Figure 1C). Immunoblot confirmed elevated protein levels of GARP and fibronectin (FN) in UUO kidneys (Figure 1, D and E). Similarly, increased expression of GARP was observed in 5/6Nx rats (Supplemental Figure 1, A–C; supplemental material available online with this article; https://doi.org/10.1172/jci.insight.203039DS1) and UIRI mice (Supplemental Figure 1, D–F). Immunohistochemistry revealed robust GARP staining in the interstitial regions of fibrotic kidneys, whereas expression was minimal in normal kidneys (Figure 1, F and G, and Supplemental Figure 1G). We next examined GARP expression in human kidneys. Microarray analysis of renal biopsy specimens from CKD patients (GSE116626) showed significantly elevated *LRRC32*, along with fibrosis markers *FN1* and *COL3A1*, compared with healthy controls (Figure 1H). To define the cellular sources of GARP in fibrotic kidneys, we re-analyzed the scRNA-seq datasets using the original cell-type annotations provided in the published study (27). Among all kidney cell populations, *Lrrc32* was predominantly expressed in myofibroblasts in the fibrotic kidney, with relatively low levels in fibroblasts and endothelial cells (Figure 1I). We next performed flow cytometry of kidney single-cell suspensions from sham and UUO mice to characterize GARP-expressing cells in fibrotic kidneys. The gating strategy used to identify fibroblasts, endothelial cells, and immune cells is shown in Supplemental Figure 1H. Briefly, fibroblasts were defined as PDGFRα^+^CD31^–^CD45^–^ cells, endothelial cells as CD31^+^CD45^–^PDGFRα^–^ cells, and immune cells as CD45^+^CD31^–^PDGFRα^–^ cells. Isotype control was used to exclude non-specific binding (Supplemental Figure 1I). Flow cytometric analysis showed that UUO markedly increased the proportion of GARP^+^LAP (TGF-β1)^+^ cells in the fibroblast population, whereas only modest increases were observed in endothelial and immune cells (Figure 1, J and K). Collectively, these data demonstrate an upregulation of GARP in human and murine fibrotic kidneys and identify fibroblasts as a major source of GARP-mediated latent TGF-β1 presentation in fibrotic kidney, suggesting a potential role of GARP-dependent TGF-β activation in the pathogenesis of kidney fibrosis.

### Global GARP deletion attenuates kidney fibrosis.

Given the critical role of TGF-β in fibrosis, we next evaluated the in vivo function of GARP using GARP-knockout (GARP-KO) mice. Because constitutive GARP deletion causes perinatal lethality due to defective palatogenesis (28), we crossed *Lrrc32^fl/fl^* mice (wild-type, WT) (29) with UBC^CreERT2^ mice to establish inducible global GARP-KO mice (UBC^CreERT2^ *Lrrc32^fl/fl^*). To this end, both WT and GARP-KO mice were administered tamoxifen before surgery and were subsequently subjected to UUO (Figure 2A). As expected, GARP expression was drastically reduced in both sham and UUO kidneys of GARP-KO compared with WT littermates (Figure 2, B–D), confirming efficient gene deletion. RT-PCR and immunoblot analysis demonstrated that ECM components including *Fn1* (FN) and *Col1a1* (COL1A1), as well as the myofibroblast marker *Acta2* (α-SMA), (Figure 2, B, C, and E) and Vimentin (Figure 2C), were markedly upregulated in fibrotic kidneys, whereas the upregulation was largely abrogated by GARP deletion (Figure 2, B–F). Consistently, Sirius red staining revealed extensive kidney fibrosis in WT-UUO mice, whereas fibrosis was substantially reduced in GARP-KO kidneys (Figure 2, E and F).

To corroborate these findings in another animal model, WT and GARP-KO mice were treated with tamoxifen and subjected to UIRI combined with contralateral uninephrectomy (Supplemental Figure 2A). Compared with WT controls, GARP-KO mice exhibited reduced ECM deposition, decreased expression of Vimentin and α-SMA, and significantly attenuated tubulointerstitial fibrosis, as assessed by Sirius red staining (Supplemental Figure 2, B–E). Notably, serum creatinine and blood urea nitrogen (BUN) levels were significantly lower in GARP-KO mice following UIRI (Supplemental Figure 2F). Together, these results demonstrate that global deletion of GARP markedly mitigates kidney fibrosis.

### Fibroblast-specific GARP deficiency ameliorates kidney fibrosis.

Given that GARP expression was predominantly enriched in myofibroblasts and, to a lesser degree, in fibroblasts of fibrotic kidneys (Figure 1I), we generated inducible, fibroblast-specific GARP conditional KO mice (Col1a2^CreERT^ × *Lrrc32^fl/fl^*, GARP-CKO) to assess the fibroblast-restricted role of GARP in kidney fibrosis. Accordingly, both genotypes (GARP-CKO and WT) were administered tamoxifen before UUO surgery, with administration continued until sacrifice to ensure sustained GARP deletion in activated fibroblasts (Figure 3A). Immunoblotting confirmed efficient GARP deletion, with reduced UUO-induced expression in GARP-CKO kidneys compared with WT controls (Figure 3, B and C), indicating fibroblasts as the major source of elevated GARP during fibrosis. Consistently, GARP-CKO mice exhibited reduced expression of FN, COL1A1, and Vimentin relative to WT controls (Figure 3, B and C). Sirius red staining revealed extensive tubulointerstitial fibrosis in WT UUO kidneys, whereas fibrosis was attenuated in GARP-CKO kidneys (Figure 3, D and E). In addition, immunohistochemical staining validated that fibroblast-specific GARP deletion weakened ECM deposition and α-SMA expression (Figure 3, D and E). Together, these findings suggest that fibroblast-expressed GARP is required for enhanced activation of fibroblasts and ECM accumulation during kidney fibrosis.

### TGF-β signaling as a major pathway driving fibroblast profibrotic activity.

Kidney fibrosis develops through complex crosstalk between injured parenchymal cells, such as tubular epithelial cells, and non-parenchymal populations, including immune-inflammatory and mesenchymal cells (30, 31). To clarify the contributions of different cell types during kidney fibrosis, we re-analyzed a published integrated single-cell and single-nucleus RNA-seq (sc/snRNA-seq) dataset comprising 219,906 single cells/nuclei from human kidneys (GSE183279) (31). This dataset included specimens from healthy individuals (Ref, *n* = 38) and patients with various forms of CKD (*n* = 28), encompassing 16 distinct cell clusters (Figure 4A). To quantify ECM-related activity, we computed an ECM score using the MSigDB Matrisome (NABA) core matrisome gene sets (collagens, ECM glycoproteins and proteoglycans) (31). Fibroblasts exhibited the highest ECM score among all cell types (Figure 4B), supporting their central role in ECM production and deposition (32–34). The above finding was further validated in the scRNA-seq dataset from UUO-kidneys (27) (Supplemental Figure 3A). Given the pivotal role of TGF-β in fibrogenesis (7), we next assessed the expression of TGF-β pathway–related genes across kidney cell types. Similar to the ECM score, fibroblasts demonstrated the highest TGF-β signaling score in both human and mouse datasets (Figure 4C and Supplemental Figure 3B). Uniform Manifold Approximation and Projection (UMAP) visualization and feature plotting showed that *LRRC32* signal was enriched in the fibroblast compartment (Supplemental Figure 3C), and violin plots further confirmed increased *LRRC32* expression in CKD compared with Ref kidneys (Supplemental Figure 3D). Gene Ontology (GO) enrichment analysis of fibroblast-specific differentially expressed genes highlighted ECM organization and TGF-β signaling as key biological processes (Figure 4D). Gene set enrichment analysis (GSEA) of the top 5 ECM score–enriched cell clusters further confirmed significant activation of TGF-β signaling in fibroblasts (Figure 4E and Supplemental Figure 3E). To spatially map the relationship among fibroblasts, ECM production, and TGF-β signaling, we analyzed a spatial transcriptomics dataset of CKD and control kidneys (31). Spatial analysis revealed colocalization of fibroblast markers with high ECM and TGF-β signaling scores (Figure 4F and Supplemental Figure 3, F–H), and *LRRC32* showed spatial enrichment in fibrotic regions (Figure 4F). Moreover, ordinary least-squares regression analysis demonstrated a positive correlation among fibroblast abundance, ECM score, and TGF-β signaling score (Figure 4G). Within fibroblasts, *LRRC32* expression was increased in CKD and positively correlated with ECM, TGF-β signaling, and fibrotic program scores (Supplemental Figure 3, I and J). Together, these analyses identify fibroblasts as key mediators of ECM accumulation in kidney fibrosis and highlight TGF-β signaling as a major pathway driving their profibrotic activity, with *LRRC32* showing fibroblast enrichment and positive association with fibrotic programs.

Because perturbation of TGF-β–related pathways in fibroblasts may affect not only matrix production but also matrix remodeling, we next performed an unbiased matrisome-focused analysis to further characterize the ECM program associated with *LRRC32*-expressing fibroblasts. Fibroblasts from the human kidney sc/snRNA-seq dataset were stratified into *LRRC32*-positive and *LRRC32*-negative populations, and differentially expressed genes annotated by the Matrisome Project were analyzed (Supplemental Figure 4A). Among detected matrisome genes, 158 were upregulated and only 1 was downregulated in *LRRC32*-positive fibroblasts (Supplemental Figure 4B and Supplemental Table 1). *LRRC32*-positive fibroblasts exhibited a broad increase in matrisome genes spanning multiple categories, including collagens, ECM glycoproteins, ECM-affiliated proteins, secreted factors, proteoglycans, and ECM regulators (Supplemental Figure 4C). Within the MMP/TIMP family of ECM regulators, MMP2 was modestly increased, whereas TIMP1, TIMP2, and TIMP3 were also significantly elevated in *LRRC32*-positive fibroblasts (Supplemental Figure 4D). These findings suggest that *LRRC32*-positive fibroblasts are associated with a coordinated matrisome program involving both matrix-producing and matrix-regulatory components.

We further examined matrisome features in the mouse kidney fibrosis scRNA-seq dataset (GSE190887). Because the sci-RNA-seq3 platform showed sparse detection of low-abundance matrisome transcripts in fibroblasts, we first evaluated matrisome gene detection rates and found that many matrisome genes were detected in only a small fraction of fibroblasts, with overall lower detection rates in the mouse dataset than in the human sc/snRNA-seq dataset (Supplemental Figure 5, A and B). Therefore, the mouse analysis was used as supportive evidence rather than as a direct one-to-one validation of all human matrisome genes. In mouse fibroblasts, *Lrrc32*-positive cells were identified and compared with *Lrrc32*-negative fibroblasts (Supplemental Figure 5C). Differential analysis showed a broad upward shift of matrisome genes in *Lrrc32*-positive fibroblasts, with 47 upregulated and no downregulated matrisome genes at the nominal significance level (Supplemental Figure 5D). To examine whether GARP loss affects fibroblast matrisome-related programs in vivo, we FACS-isolated PDGFRα-positive fibroblasts from WT and fibroblast-specific GARP-CKO mice after UUO for RT-PCR analysis (Supplemental Figure 5E). GARP-deficient fibroblasts showed reduced expression of selected fibrotic ECM genes, including *Col1a1* and *Col3a1*, whereas *Col1a2* showed no significant change (Supplemental Figure 5F). Among matrix-remodeling genes, *Mmp2* showed a modest, nonsignificant decrease (*P* = 0.1504), whereas *Timp2* was reduced to a greater extent (*P* < 0.01) in GARP-deficient fibroblasts (Supplemental Figure 5F). Together, these data suggest that *LRRC32*/GARP is associated with a coordinated fibroblast matrisome program and that GARP deletion attenuates selected matrix-producing and TIMP2-associated matrix-stabilizing responses.

### GARP-dependent TGF-β activation sustains Smad signaling and induces fibroblast stimulation.

Because global or fibroblast-specific deletion of GARP attenuated kidney fibrosis, we next examined how GARP regulates TGF-β signaling in fibroblasts and contributes to their activation. GARP is known to tether latent TGF-β on the plasma membrane of blood cells and hepatic stellate cells (18, 35), thereby providing a platform for TGF-β activation. In agreement with this mechanism, flow cytometric analysis showed that fibroblast-specific deletion of GARP markedly reduced the proportion of GARP^+^LAP (TGF-β1)^+^ fibroblasts following UUO (Figure 5, A and B). Moreover, immunoblot analysis revealed robust induction of p-Smad2/3 after UUO (Figure 5, C and D) and UIRI (Supplemental Figure 6, A and B), which was significantly reduced in GARP-KO kidneys compared with WT controls. These results led us to hypothesize that GARP promotes the local activation of latent TGF-β1, thereby sustaining TGF-β signaling during kidney fibrosis. Indeed, a TGF-β reporter assay (36) showed impaired generation of active TGF-β in GARP-deficient primary kidney fibroblasts (PKFs) (Figure 5E and Supplemental Figure 7, A and B). Upon TGF-β1 stimulation, PKFs from WT mice exhibited elevated p-Smad2/3 levels that persisted for at least 48 hours, whereas GARP deletion clearly blunted Smad2/3 phosphorylation (Figure 5, F and G). Because GARP expression is relatively low at baseline, we reasoned that newly induced GARP may reinforce TGF-β activation upon stimulation. To test whether GARP upregulation promotes TGF-β activation, we overexpressed GARP in normal rat kidney fibroblasts (NRK-49F) using lentivirus (LV) (Figure 5H). Immunoblot analysis showed that, compared with control cells (LV-vector), GARP-overexpressing NRK-49F cells (LV-GARP) exhibited increased levels of mature TGF-β1 (Figure 5I), regardless of exogenous TGF-β1 stimulation. Because no antibody is available to detect rat latent TGF-β1, we were unable to directly assess whether GARP overexpression increased the surface docking of latent TGF-β1. To address this, we performed a TGF-β reporter assay, which showed that GARP overexpression significantly increased active TGF-β levels in the supernatant compared with the control, an effect abolished by incubation with the αv inhibitor CWHM-12 (Figure 5J). Consistently, Smad2/3 phosphorylation was enhanced in GARP-overexpressing cells (Figure 5, K and L), which could be also blocked by CWHM-12 (Supplemental Figure 7C) or pharmacological inhibition of TGF-β receptor I with SB431542 (Supplemental Figure 7D). Together, these findings demonstrate that upregulation of GARP enables integrin-dependent activation of latent TGF-β by fibroblasts, thereby sustaining TGF-β signaling.

We next assessed the impact of GARP-dependent TGF-β activation on fibroblast phenotype. Activated fibroblasts are characterized by increased ECM production and elevated expression of filament proteins such as α-SMA and Vimentin (6). In PKFs, TGF-β1 stimulation induced upregulation of GARP, ECM proteins (FN and COL1A1), and Vimentin. Deletion of GARP prevented the TGF-β1–induced upregulation of ECM proteins and Vimentin (Figure 5, M and N). Conversely, GARP overexpression in NRK-49F cells enhanced FN and α-SMA expression compared with the vector control (Figure 5, O and P). Thus, these findings indicate that increased expression of GARP amplifies TGF-β signaling to promote renal fibroblast activation.

### TNF upregulates GARP in an NF-κB–dependent fashion and synergizes with TGF-β1.

Since GARP expression was upregulated in both fibrotic kidneys and activated fibroblasts, we next sought to elucidate the mechanisms underlying its induction. Using Harmonizome 3.0 (37), we searched for putative transcription factors regulating GARP expression (https://maayanlab.cloud/Harmonizome/gene/LRRC32). After cross-referencing results from ChEA Transcription Factor Targets 2022 and JASPAR Predicted Transcription Factor Targets (https://maayanlab.cloud/Harmonizome/gene/LRRC32), NFKB1 emerged as the remaining candidate (Figure 6A), in line with prior studies showing NF-κB binding to the *Lrrc32* promoter (38) and enhancer (26). Furthermore, in human kidney single-cell transcriptomic data (GSE183279) (31), fibroblast-restricted comparison between CKD and Ref further showed significant enrichment of the Hallmark TNFA signaling via NF-κB gene set, indicating activation of NF-κB–related transcriptional programs in fibroblasts under CKD condition (Figure 6B). Given that NF-κB signaling is tightly linked to kidney inflammation and fibrosis (39) and can be activated by cytokines such as TNF (40), we examined whether TNF regulates GARP expression through NF-κB. Stimulation of NRK-49F fibroblasts with TNF induced a time-dependent increase in GARP expression (Figure 6, C and D). During UUO-induced kidney fibrosis, upregulation of *Nfkb1* preceded that of *Lrrc32* (Supplemental Figure 8A). Similarly, TNF rapidly activated NF-κB signaling in NRK-49F cells, as evidenced by increased phosphorylation of P105 (NF-κB1) and P65 and reduced IκBα expression, preceding the subsequent induction of GARP protein expression (Supplemental Figure 8B). Finally, pharmacological inhibition of NF-κB with pyrrolidinedithiocarbamate (PDTC) blocked TNF-induced GARP expression (Figure 6, E–G). Guided by the consensus NF-κB binding sequence 5′-GGGRNNNYCC-3′ (where R = A or G, Y = C or T, and N = any nucleotide) (41), we identified 2 potential NF-κB binding sites (NBSs) in the rat *Lrrc32* promoter. Cross-species sequence alignment showed that the promoter NBS (NBS1) is highly conserved across rat, human, mouse, macaque, and dog, whereas NBS2 is less well conserved (Figure 6H). Chromatin immunoprecipitation followed by quantitative PCR (ChIP-qPCR) confirmed that TNF increased P65 occupancy at NBS1 and, to a lesser extent, at NBS2 (Figure 6I). Interestingly, TNF increased P65 occupancy not only at the *Lrrc32* promoter but also at the previously reported distal enhancer (26) (Figure 6I). These data indicate that TNF-induced NF-κB directly binds both proximal and distal regulatory elements of the *Lrrc32* locus in fibroblasts.

Given that GARP expression was elevated in activated fibroblasts and TGF-β1 is a central driver of fibroblast activation during kidney fibrosis, we next examined whether TGF-β1 also regulates GARP expression. TGF-β1 induced a time-dependent upregulation of GARP (Figure 6, J–L). Interestingly, co-stimulation with TNF and TGF-β1 synergistically enhanced *Lrrc32* mRNA and GARP protein expression, exhibiting a greater effect than either cytokine alone (Figure 6, M and N). Together, these findings demonstrate that TNF/NF-κB signaling engages evolutionarily conserved regulatory elements of *Lrrc32* in renal fibroblasts and that TNF synergizes with TGF-β1 to reinforce GARP upregulation during fibroblast activation.

### Therapeutic deletion of GARP attenuates established kidney fibrosis.

Lastly, we asked whether therapeutic deletion of GARP could mitigate established fibrosis. To this end, kidney fibrosis was first allowed to develop for 7 days in inducible GARP-CKO UUO mice, after which tamoxifen was administered for 5 consecutive days to induce GARP deletion (Figure 7A). Immunoblots confirmed reduced renal GARP expression, along with attenuated induction of FN, COL1A1, and Vimentin on day 14 compared with controls (Figure 7, B and C). Sirius red staining showed comparable fibrosis between the 2 groups on day 7. However, following tamoxifen-induced GARP deletion, fibrosis progressed to a much greater extent in control than in GARP-CKO mice (Figure 7, D and E). Immunohistochemistry also confirmed reduced α-SMA expression after GARP deletion (Figure 7F). To extend these findings, we next induced global GARP deletion after fibrosis onset, which similarly reduced collagen deposition, ECM accumulation, and Smad2/3 phosphorylation, yielding a more pronounced antifibrotic response than fibroblast-specific deletion (Supplemental Figure 9). Collectively, these results demonstrate that therapeutic deletion of GARP effectively attenuates ongoing kidney fibrosis, highlighting GARP as a promising therapeutic target.

## Discussion

TGF-β signaling is one of the most potent drivers of organ fibrosis, including in the kidney (7). While previous studies have primarily focused on TGF-β synthesis and downstream signaling (42–44), the precise mechanisms controlling the activation of latent TGF-β, an indispensable step for its biological activity, have been largely overlooked in the context of kidney fibrosis. The present study extends the role of GARP to the context of kidney fibrosis and identifies it as a fibroblast-associated regulator of profibrotic activation. We demonstrate that GARP expression is upregulated in both human and experimental models of CKD. Genetic ablation of GARP, either globally or specifically in fibroblasts, attenuates ECM accumulation, myofibroblast activation, and TGF-β signaling, thereby mitigating kidney fibrosis. Moreover, the inflammatory cytokine TNF cooperates with TGF-β1 to upregulate GARP expression, establishing a feed-forward loop that amplifies fibroblast activation and ECM deposition. Notably, therapeutic deletion of GARP after fibrosis onset effectively halts disease progression, underscoring its translational potential as a selective antifibrotic target.

Although GARP was initially characterized in immune cells such as Tregs (20), our findings extend its relevance to mesenchymal cells in fibrotic kidneys. scRNA-seq analyses revealed that GARP expression is predominantly localized to fibroblasts and myofibroblasts in fibrotic kidney, aligning with their high TGF-β signaling and ECM production scores. Conditional deletion of GARP in fibroblasts markedly reduced Smad2/3 phosphorylation and ECM gene expression, demonstrating that fibroblast-intrinsic GARP is required for maintaining autocrine TGF-β signaling loops. This finding positions GARP as a mechanistic bridge linking profibrotic cytokine signaling with persistent fibroblast activation, reminiscent of its role in hepatic stellate cells during liver fibrosis (18). The partial but not complete suppression of fibrosis in fibroblast-specific KOs suggests that GARP expression in other cell types, such as endothelial cells or pericytes, may also contribute to disease progression, a hypothesis warranting future investigation.

Kidney fibrogenesis advances in distinct stages, beginning with the establishment of a profibrotic niche and ultimately culminating in widespread fibrosis (11, 30). This process is driven by a dynamic interplay of tubular epithelial injury (45), inflammatory infiltration (46), and fibroblast activation (6), the latter representing the central event (6). Because TGF-β is a principal inducer of fibroblast responses, the spatially restricted activation of latent TGF-β likely represents a key determinant of local fibroblast stimulation and ECM deposition. Supporting this idea, our re-analysis of publicly available scRNA-seq and spatial transcriptomics datasets revealed a strong spatial correlation between TGF-β signaling, fibroblast activation, and ECM score (Figure 4), suggesting that sustained local generation of active TGF-β could be a defining feature of the “profibrotic niche” (11). However, inducible *Tgfbr2* deletion in matrix-producing interstitial or PDGFRβ^+^ cells, while suppressing Smad signaling and profibrotic gene expression, does not translate into reduced tissue-level collagen deposition in kidney injury models (47, 48). Residual ECM production, impaired matrix turnover, compensatory profibrotic pathways, incomplete recombination, and contributions from non-fibroblast compartments can collectively sustain fibrosis despite reduced canonical signaling (49, 50). In this context, GARP deletion may not phenocopy fibroblast-specific *Tgfbr2* deletion, because it is expected to reduce the local activation of latent TGF-β rather than fully abolish receptor-mediated signaling. This distinction may lead to different effects on fibroblast matrisome remodeling, matrix turnover, and paracrine crosstalk within the fibrotic niche. Although the present study was not designed to comprehensively profile the fibroblast matrisome in an unbiased manner, such analyses will be important in future work to clarify the differential consequences of GARP loss versus direct interruption of TGF-β receptor signaling. In addition, altered ECM-remodeling programs, including matrix metalloproteinase–related pathways, may also contribute to these divergent phenotypes. In addition to its soluble functions, TGF-β1 can be activated allosterically, as integrin αvβ8 binding remodels the latent complex to expose its active domain without release (51). In this setting, fibroblasts may function as GARP-driven signaling hubs that reinforce their own activation and propagate fibrotic responses to neighboring cells. In addition, crosstalk between inflammatory and fibrogenic pathways underlies the progression of chronic kidney injury. Our results demonstrate that NF-κB directly binds to the *Lrrc32* promoter and mediates transcriptional upregulation of GARP in response to TNF. In addition to NBS1, our ChIP-qPCR data showed that TNF increased p65 occupancy at the previously reported distal *Lrrc32* +70 kb enhancer in fibroblasts (26), suggesting that this enhancer is also engaged under inflammatory stimulation. However, whether the distal enhancer functionally cooperates with the promoter to regulate *Lrrc32* transcription in myofibroblasts remains to be determined. The synergistic induction of GARP by TNF and TGF-β1 provides a mechanistic explanation for how inflammatory cues amplify profibrotic TGF-β signaling. This dual regulation may establish a self-reinforcing loop in which inflammatory cytokines upregulate GARP, while GARP sustains local TGF-β activation and fibroblast differentiation. Consistent with this model, recent single-cell transcriptomic analyses revealed that concurrent activation of TGF-β and TNF pathways is enriched in NKD2^+^ myofibroblasts, a population crucial for collagen production and matrix remodeling in fibrotic kidneys (5).

Despite extensive effort to therapeutically modulate the TGF-β pathway, clinical translation has been hampered by its pleiotropic and context-dependent nature (10). Because TGF-β is broadly expressed and regulates various physiological processes, global inhibition strategies, such as pan-neutralizing antibodies and receptor kinase inhibitors, have invariably led to toxicities, including impaired wound healing, chronic inflammation, and cardiovascular abnormalities (8). Our finding that deletion of GARP after fibrosis onset attenuates collagen deposition and ECM accumulation supports the potential of targeting GARP as an alternative strategy to modulate profibrotic TGF-β activity. By regulating the activation of latent TGF-β at the cell surface, GARP represents a distinct intervention point targeting the TGF-β pathway. However, the present study did not directly compare GARP targeting with pharmacological inhibition of TGF-β or NF-κB signaling, and therefore does not establish whether targeting GARP provides greater antifibrotic efficacy, selectivity, or safety than direct inhibition of these pathways. Nevertheless, our findings provide a rationale for future evaluation of approaches targeting the GARP–latent TGF-β complex in kidney fibrosis.

Several limitations of the present study should be acknowledged. First, as noted above, we did not directly compare GARP targeting with pharmacological inhibition of TGF-β or NF-κB signaling, and the relative efficacy, selectivity, and safety of these approaches thus remain undetermined. Second, although GARP overexpression promoted TGF-β signaling and increased the profibrotic markers FN and α-SMA in cultured fibroblasts, these experiments do not establish whether GARP upregulation alone is sufficient to initiate kidney fibrosis in vivo in the absence of tissue injury or inflammatory signals. Thus, our findings support an important role for GARP in amplifying and sustaining profibrotic signaling in the injured kidney but do not establish GARP as an initiator of fibrosis. Finally, although our study defines the role and regulation of fibroblast-associated GARP in kidney fibrosis, GARP has previously been implicated in fibrotic responses in other organs, including the liver. Therefore, the broader concept of GARP as a profibrotic regulator is not unique to the kidney.

## Methods

### Sex as a biological variable.

Because sex differences in susceptibility to UUO (52), IRI (53), and 5/6Nx (54) rodent models are well recognized, animal experiments were conducted in male mice or rats. Whether the findings observed in males extend to females remains an important question for future investigation.

### Mice.

C57BL/6 mice and Sprague-Dawley rats were obtained from the Animal Centre of Southern Medical University. For inducible global deletion of *Lrrc32*, *Lrrc32^fl/fl^* mice were obtained as previously described (29) and were crossed with UBC^CreERT2^ mice (007001, Jackson Laboratory) to generate UBC^CreERT2^ *Lrrc32^fl/fl^* mice (GARP-KO). Cre activity was induced by i.p. injection of tamoxifen (Sigma-Aldrich) at 30 mg/kg/day. For fibroblast-specific deletion of *Lrrc32*, *Lrrc32^fl/fl^* mice were crossed with Col1a2^CreER^ mice (029567, Jackson Laboratory) to generate Col1a2^CreER^ *Lrrc32^fl/fl^* mice (GARP-CKO). Tamoxifen was administered i.p. at 30 mg/kg/day for 5 consecutive days before induction of kidney fibrosis and was continued after UUO until sacrifice to ensure deletion of *Lrrc32* in newly generated myofibroblasts. For therapeutic deletion of *Lrrc32*, kidney fibrosis was allowed to develop for 7 days in UUO mice before tamoxifen was administered for 5 days. Sex-matched *Lrrc32^fl/fl^* littermates served as controls and received the same tamoxifen dosing schedule.

### UUO model.

Eight- to 10-week-old male mice were anesthetized with sodium pentobarbital (30 mg/kg, i.p.). To induce UUO, the left ureter was exposed and tightly ligated near the lower pole of the kidney using a 4-0 silk suture. Sham-operated animals underwent identical surgical manipulation except that the ureter was left unligated. Postoperative analgesia was provided by subcutaneous administration of meloxicam. Kidneys were collected 7 or 14 days after surgery for downstream analyses.

### UIRI model.

Mice were anesthetized with sodium pentobarbital (30 mg/kg, i.p.) and a midline abdominal incision was made to expose the kidneys. The left renal pedicle was occluded using a microvascular clamp for 35 minutes to induce ischemia, while body temperature was maintained at 37°C on a heating pad. Reperfusion was confirmed by observing the restoration of kidney color immediately after clamp removal. The incision was then closed in layers, and animals were allowed to recover with unrestricted access to food and water. Postoperative analgesia was provided by subcutaneous administration of meloxicam. The contralateral (right) kidney was removed 10 days after reperfusion, and mice were euthanized on day 11 for collection of blood and kidney samples for subsequent analyses.

### 5/6Nx model.

Progressive CKD was established in Sprague-Dawley rats using the 5/6Nx procedure. Under anesthesia, approximately two-thirds of the left kidney were removed by surgically excising the upper and lower poles through a left flank incision. Postoperative analgesia was provided by subcutaneous administration of meloxicam. After a 7-day recovery period, the right kidney was completely excised via a right flank incision to accomplish subtotal nephrectomy. Rats were euthanized 35 days after the initial surgery, and kidney tissues were harvested for subsequent analyses.

### sc/snRNA-seq data analysis.

Two datasets were analyzed: GSE183279 (human kidney integrated sc/snRNA-seq), which includes kidney samples from healthy controls (acute kidney injury samples were excluded) and patients with CKD; and GSE190887, which contains single-cell transcriptomes from mouse models of kidney fibrosis. For GSE183279, we downloaded the author-provided processed AnnData (.h5ad) from CZ CELLxGENE (https://cellxgene.cziscience.com/collections/bcb61471-2a44-4d00-a0af-ff085512674c) with cell-type annotations and performed gene-signature scoring in Scanpy (https://scanpy.readthedocs.io/). For GSE190887, we used the processed expression matrix and metadata from the original study (27).

The ECM activity gene signature scoring method used in this study was based on the approach proposed by Abedini et al. (32). Specifically, the ECM score is calculated by combining the expression of collagens, glycoproteins, and proteoglycans (Supplemental Table 2). The TGF-β signaling gene set was retrieved from MSigDB (KEGG_TGF_BETA_SIGNALING_PATHWAY, M2642; https://www.gsea-msigdb.org/gsea/msigdb/cards/KEGG_TGF_BETA_SIGNALING_PATHWAY), and the cell annotations and scoring, including for fibroblasts, were derived from the code uploaded in Lake et al. (31), specifically using the “subclass.l1” field for cell annotation and scoring. Functional pathway activity at the single-cell level was evaluated using the scanpy.tl.score_genes function. Pathway-level enrichment was evaluated by GSEA implemented in clusterProfiler(https://bioconductor.org/packages/clusterProfiler/) under default settings. In addition, spatial transcriptomics data from CKD patients and healthy controls (GSE183456) were analyzed to explore the spatial colocalization of ECM activity, TGF-β signaling, and fibroblast-associated transcriptional programs. Signature scores for each program were computed using the same approach as for the scRNA-seq datasets. Correlations among spatial features were assessed using ordinary least-squares regression implemented in statsmodels (https://www.statsmodels.org/).

### Matrisome analysis in human and mouse fibroblasts.

For human fibroblast matrisome analysis, fibroblasts were extracted from the human kidney sc/snRNA-seq dataset and stratified into *LRRC32*-positive and *LRRC32*-negative populations based on detectable *LRRC32* expression. Matrisome genes were annotated using the Matrisome Project (https://sites.google.com/uic.edu/matrisome/home?pli=1&authuser=0). Differential expression analysis between *LRRC32*-positive and *LRRC32*-negative fibroblasts was performed using the Wilcoxon rank-sum test implemented in Scanpy.

For the mouse kidney fibrosis single-cell dataset, fibroblast-lineage cell populations were extracted based on the original cell-type annotations from GSE190887. *Lrrc32*-positive and *Lrrc32*-negative fibroblast-lineage cells were defined according to detectable *Lrrc32* expression. Given the sparse detection of low-abundance ECM transcripts in this sci-RNA-seq3 dataset, we first evaluated matrisome gene detection rates in fibroblast-lineage cells and compared detection across different thresholds. Mouse matrisome genes were annotated using the Matrisome Project, and differential expression analysis of detected matrisome genes between *Lrrc32*-positive and *Lrrc32*-negative fibroblast-lineage cells was performed using the Wilcoxon rank-sum test implemented in Scanpy.

### RT-PCR.

Total RNA was extracted from kidney tissues or cultured cells using TRIzol reagent (R401, Vazyme). For FACS-isolated cells, RNA was extracted using the FastPure Micro Cell/Tissue RNA Isolation Kit (RC350-01, Vazyme). cDNA was synthesized from purified RNA using a HiScript III 1st Strand cDNA Synthesis Kit (R323, Vazyme) according to the manufacturer’s instructions. Quantitative RT-PCR was performed on 7500 Fast Real-Time PCR System (Applied Biosystems). Relative mRNA levels of target genes were calculated using the 2^−ΔΔCt^ method after normalization to GAPDH. Primer sequences are listed in Supplemental Table 3.

### Immunoblot.

Proteins were extracted from cultured cells or kidney tissues using RIPA lysis buffer supplemented with 1% protease and phosphatase inhibitor cocktail. Protein concentrations were determined with the Pierce BCA Protein Assay Kit (23227, Thermo Fisher Scientific). Equal amounts of protein were mixed with 5× loading buffer, boiled for denaturation, and resolved on 10% SDS-polyacrylamide gels by electrophoresis. Separated proteins were transferred onto nitrocellulose membranes (HATF00010, Merck Millipore), which were then blocked and incubated with primary antibodies at 4°C overnight. After incubation with appropriate secondary antibodies, signals were visualized using a MiniChemi 910 Plus chemiluminescent imaging system (Sagecreation). Band intensities were quantified with ImageJ software (NIH). Detailed information on the brands and working concentrations of the antibodies used is provided in Supplemental Table 4.

### Sirius red staining.

Mouse kidneys were fixed in 4% paraformaldehyde, embedded in paraffin, and sectioned at 4 μm thickness using a microtome (Leica). Tissue sections were deparaffinized and rehydrated through a graded ethanol series prior to staining. Collagen deposition was visualized by staining with Sirius red solution (BA4356, Baso) for 15 minutes, following the manufacturer’s protocol. The extent of fibrosis was quantified as the percentage of Sirius red–positive area using ImageJ software.

### Immunohistochemistry.

Paraffin-embedded kidney sections (4 μm) were deparaffinized and rehydrated through graded ethanol, followed by antigen retrieval via heat-induced epitope recovery. Endogenous peroxidase activity was quenched with 3% H_2_O_2_ at room temperature. After blocking with 10% donkey serum, sections were incubated overnight at 4°C with primary antibodies against α-SMA, FN, COL1A1, and GARP. Following PBS washes, α-SMA, FN, and COL1A1 were visualized using the AEC chromogen kit (SK-4200, Vector), while GARP was detected with the DAB chromogen kit (SK-4100, Vector). Nuclei were counterstained with hematoxylin, and slides were imaged using an Olympus BX53 (LED) microscope (Olympus). Image processing and quantitative analysis were performed using ImageJ software. Details of all antibodies are listed in Supplemental Table 4.

### Kidney single-cell preparation.

The kidneys were harvested and thoroughly minced using sterile surgical blades. Each kidney tissue sample was incubated with 3 mL of digestion buffer containing collagenase IV (1 mg/mL; LS004188, Worthington) and DNase I (100 U/mL; BS137, Biosharp) in RPMI medium supplemented with 2% FBS. The tissues were digested at 37°C for 40 minutes with agitation at 70 rpm. Following digestion, the cell suspension was resuspended in ice-cold RPMI medium containing 2% FBS and passed through a 70 μm cell strainer (Biosharp). Cells were collected by centrifugation at 300*g* for 5 minutes at 4°C. The cell pellet was resuspended in red blood cell lysis buffer (0.15 M NH_4_Cl, 10 mM KHCO_3_, and 0.1 mM Na_2_EDTA) and incubated at room temperature for 4 minutes to remove erythrocytes. Subsequently, cells were washed with RPMI medium containing 10% FBS, and the resulting renal single-cell suspension was used for flow cytometric staining.

### Flow cytometry.

For staining of mouse primary kidney fibroblast, cells were incubated with anti-CD16/CD32 antibody (101320, BioLegend) for 5 minutes to block Fc receptors. Cells were then resuspended in FACS buffer (PBS containing 0.5% BSA and 2 mM EDTA) and incubated on ice for 30 minutes with APC-GARP (17-9891-82, eBioscience), PE-PDGFRα (12-1401-81, eBioscience), BV421-LAP (565638, BD Biosciences), PE-Cy7-CD31 (561410, BD Biosciences), BV510-CD45 (103138, BioLegend), APC-rat IgG2a, κ (GARP isotype control, 400512, BioLegend), BV421-rat IgG1, κ (LAP isotype control, 562438, BD Biosciences), and APC-Cy7 Fixable Viability Dye (65-0865-14, eBioscience). After staining, cells were washed with FACS buffer and passed through a 40 μm cell strainer (Biosharp). Flow cytometric acquisition and sorting of fibroblasts were performed using BD FACSAria Fusion. To evaluate the cell-surface expression of mouse GARP, NRK-49F cells were incubated with an APC-conjugated anti-GARP antibody in the presence of Fixable Viability Dye (65-0865-18, eBioscience) at 4°C for 30 minutes. After washing, samples were analyzed using a BD FACSCanto II flow cytometer. Data were analyzed using FlowJo. Detailed information on antibodies is provided in Supplemental Table 4.

### Cell culture and treatments.

HEK293T (CRL-3216, ATCC) and NRK-49F (CRL-1570, ATCC) cells were maintained in DMEM or DMEM/Nutrient Mixture F-12 (DMEM/F12) supplemented with 10% FBS and 1% penicillin-streptomycin. For stimulation assays, NRK-49F cells were seeded in 6-well plates and cultured in complete medium for 24 hours, followed by serum reduction to 1% FBS for synchronization. Cells were then treated with recombinant mouse TNF (10 ng/mL; 410-MT-010, R&D Systems) or recombinant human TGF-β1 (2 ng/mL; Z03411, GenScript), as indicated. For inhibitor studies, NRK-49F cells were pretreated with the NF-κB inhibitor PDTC (0.5 μM; HY-18738, MedChemExpress) for 1 hour before stimulation with TNF. The integrin inhibitor CWHM-12 (100 nM; HY-18644, MedChemExpress) was co-administered with TGF-β1 during treatment. The TGF-β receptor kinase inhibitor SB-431542 (1.5 μM; HY-10431, MedChemExpress) was added 24 hours after TGF-β1 stimulation.

PKFs were isolated as previously reported (55, 56). Briefly, kidneys were excised and immediately placed in ice-cold PBS, then minced into small fragments and digested with 0.25% trypsin-EDTA for 30 minutes at 37°C. The resulting cell suspension was filtered through a 70-μm strainer and centrifuged at 800*g* for 10 minutes. The pellet was resuspended in DMEM/F12 medium containing 10% FBS, and cultures were maintained under standard conditions. The culture medium was refreshed on day 3, and PKFs were confirmed by Vimentin staining. Before experimental stimulation, PKFs were serum-starved overnight in medium containing 1% FBS.

### TGF-β reporter assay.

NRK-49F or PKF cells were cultured with recombinant TGF-β1 for 48 hours, after which the culture supernatants were collected for analysis of active TGF-β levels. TGF-β bioactivity was assessed using NIH-3T3 reporter cells stably expressing a firefly luciferase gene driven by 4 tandem SMAD-binding elements (provided by Bing Sun, Center for Excellence in Molecular Cell Science, Shanghai, China) (36). Reporter cells were seeded into 24-well plates at a density of 1 × 10^5^ cells per well, reaching approximately 80% confluence the following day. After overnight incubation in serum-free medium to minimize background activity from serum-derived TGF-β, the cells were treated for 16 hours with conditioned media collected from NRK-49F or PKF cultures. TGF-β activity was then quantified by measuring luminescence using the Luciferase Assay System (Promega) according to the manufacturer’s protocol.

### Generation of GARP-overexpressing NRK-49F cell line.

To generate NRK-49F cells stably overexpressing GARP, pUltra (24129, Addgene) carrying the coding sequence of *Lrrc32* (NM_001113379) was co-transfected with the packaging plasmids into HEK293T cells. Empty pUltra vector served as the negative control. Viral supernatant was collected and filtered through a 0.45-μm filter. NRK-49F cells were then infected with the viral supernatant in the presence of 8 μg/mL polybrene (Sigma-Aldrich). After 72 hours, transduced cells were sorted using a BD FACSAria Fusion cell sorter to generate stable cell lines. GARP overexpression was validated by flow cytometry and immunoblotting.

### Immunofluorescence.

PKFs were fixed with 4% paraformaldehyde for 15 minutes, permeabilized with 0.1% Triton X-100 in PBS for 15 minutes, and subsequently blocked with 10% donkey serum for 1 hour at room temperature. Cells were then incubated overnight at 4°C with anti- Vimentin. After washing, samples were incubated for 1 hour at room temperature with donkey anti-goat Alexa Fluor 594 and donkey anti-rabbit Alexa Fluor 488 secondary antibodies. Nuclei were counterstained with DAPI Fluoromount (SouthernBiotech). Fluorescence images were captured using an Olympus FV1000 confocal microscope. Detailed antibody information is listed in Supplemental Table 4.

### ChIP assay.

ChIP assays were performed using the SimpleChIP Plus Sonication Chromatin IP Kit (56383, Cell Signaling Technology) following the manufacturer’s protocol. Chromatin was immunoprecipitated with a rabbit anti–NF-κB p65 antibody (A00284-1, BOSTER). The recovered DNA was analyzed by qPCR to evaluate p65 occupancy at the rat *Lrrc32* locus, including 2 predicted NBSs in the proximal promoter region, NBS1 and NBS2, and the previously reported distal *Lrrc32* +70 kb enhancer. Primer sequences used for ChIP-qPCR are listed in Supplemental Table 3. Relative enrichment was calculated using the 2^−ΔΔCt^ method and normalized to a 100-fold diluted input DNA sample.

### Statistics.

All data analyses were performed using Prism 9.0 (GraphPad Software) or R version 4 (https://www.r-project.org/). Quantitative data are presented as mean ± SEM, and for all in vivo experiments, *n* values denote biological replicates. Blinding was implemented during animal surgeries, tissue collection, and image quantification to minimize bias. Comparisons between 2 groups were conducted using an unpaired 2-tailed Student’s *t* test, whereas 1-way or 2-way ANOVA was applied for multiple group comparisons, as appropriate to the experimental design. Data normality was verified using the Shapiro-Wilk test. Pearson’s correlation analysis was used to determine correlation coefficients (*r*) and *P* values, and linear regression was performed to assess relationships between variables.

### Study approval.

Animal experiments and protocols were approved by the Ethics Committee of Animal Breeding and Experimentation of Southern Medical University.

### Data availability.

The underlying data values for the main and supplemental figures are provided in the Supporting Data Values file. Publicly available transcriptomic datasets of mouse and human kidney tissues, as well as single-cell combinatorial indexed RNA-seq datasets, were retrieved from the NCBI Gene Expression Omnibus (GEO) under accession numbers GSE183279, GSE190887, GSE116626, and GSE118339.

## Author contributions

YH, FFH, and FW conceived and designed the study, supervised the project, and wrote the manuscript. YC, WX, and JY contributed to study design, performed experiments, and conducted data analysis. PD was involved in animal experiments and pathological staining. NL carried out the scRNA-seq and spatial transcriptomics analyses. YL assisted with in vitro experiments. Hui Zhou and Hong Zhou assisted with partial data organization and management. JW and BZ provided technical and methodological support. YC, WX, and JY contributed equally to this work.

## Conflict of interest

The authors have declared that no conflict of interest exists.

## Funding support

- The National Natural Science Foundation of China (grants 82090022 and 82171725) to YH.
- Guangdong Basic and Applied Basic Research Foundation (grant 2023A1515010257) to YH.
- The National Natural Science Foundation of China (Key Program) (grant 82330020) to FFH.
- The Program of Introducing Talents of Discipline to Universities, 111 Plan (grant D18005) to FFH.
- Key Technologies R&D Program of Guangdong Province (grant 2023B1111030004) to FFH.

## Supplementary Material

Supplemental data

Unedited blot and gel images

Supplemental table 1

Supplemental table 2

Supplemental table 3

Supplemental table 4

Supporting data values

## Figures and Tables

**Figure 1:**
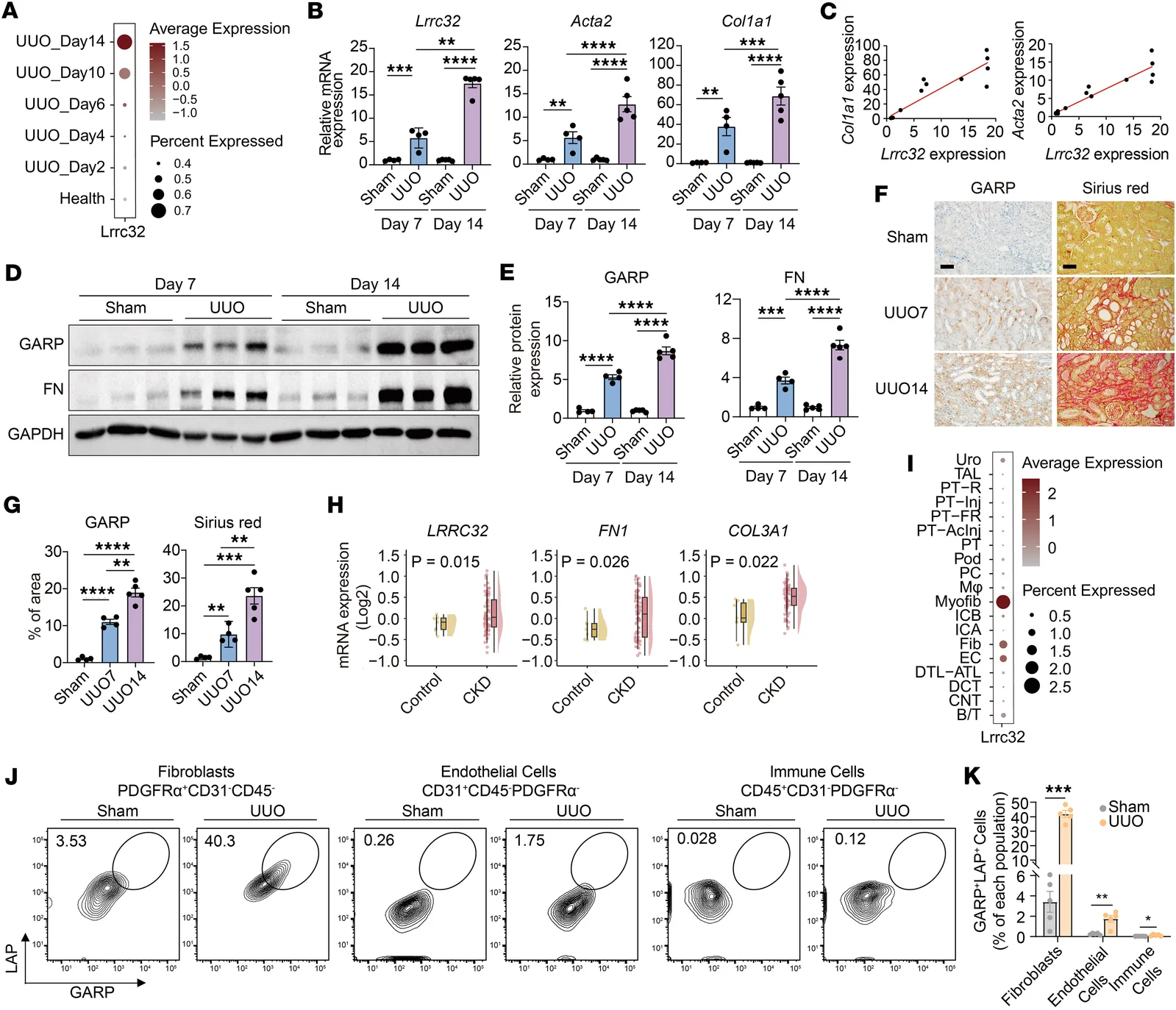
Increased expression of GARP in kidneys of fibrotic mice and CKD patients. (**A**) scRNA-seq analysis for *Lrrc32* expression in the kidneys of mice at different days after UUO. (**B**) mRNA levels of *Lrrc32* and fibrotic markers in mouse kidneys (day 7 or day 14 after surgery) (*n* = 4–5). Sham-operated mice were used as negative controls. (**C**) Pearson’s correlation analysis between *Lrrc32* and fibrotic marker expression levels. (**D** and **E**) Representative (**D**) and quantification (**E**) of immunoblot for GARP and FN expression in UUO kidneys. Each lane represents 1 mouse. FN, fibronectin. (**F** and **G**) Representative (**F**) and quantification (**G**) of immunohistochemical staining for GARP (left) and Sirius red staining (right) of mouse kidney sections. Scale bars: 50 μm. (**H**) Microarray analysis of *LRRC32*, *FN*, and *COL3A1* in renal biopsy specimens from control and CKD patients. (**I**) Bubble plot comparing expression of *Lrrc32* in various cell types in mouse UUO kidneys. (**J** and **K**) Representative flow cytometry plots (**J**) and quantification (**K**) of GARP^+^LAP (TGF-β1)^+^ cell frequencies among fibroblasts, endothelial cells, and immune cells from sham and UUO kidneys. Uro, urothelium; TAL, thick ascending limb of LoH; PT-R, repairing proximal tubule; PT-Inj, injured PT; PT-FR, failed repair PT cells; PT-AcInj, acute injury PT; Pod, podocyte; PC, principal cell of collecting duct; Mφ, macrophage; Myofib, myofibroblast; ICB, type B intercalated cell of collecting duct; ICA, type A intercalated cell of collecting duct; Fib, fibroblast; EC, endothelial cell; DTL, descending limb of loop of Henle (LoH); ATL, thin ascending limb of LoH; DCT, distal convoluted tubule; CNT, connecting tubule; B/T, B and T cells. Data represent mean ± SEM. *P < 0.05; **P < 0.01; ***P < 0.001; ****P < 0.0001. Statistical significance was determined by 2-way ANOVA followed by Tukey’s multiple-comparison test (**B** and **E**) and unpaired 2-tailed Student’s *t* test (**G**, **H**, and **K**).

**Figure 2:**
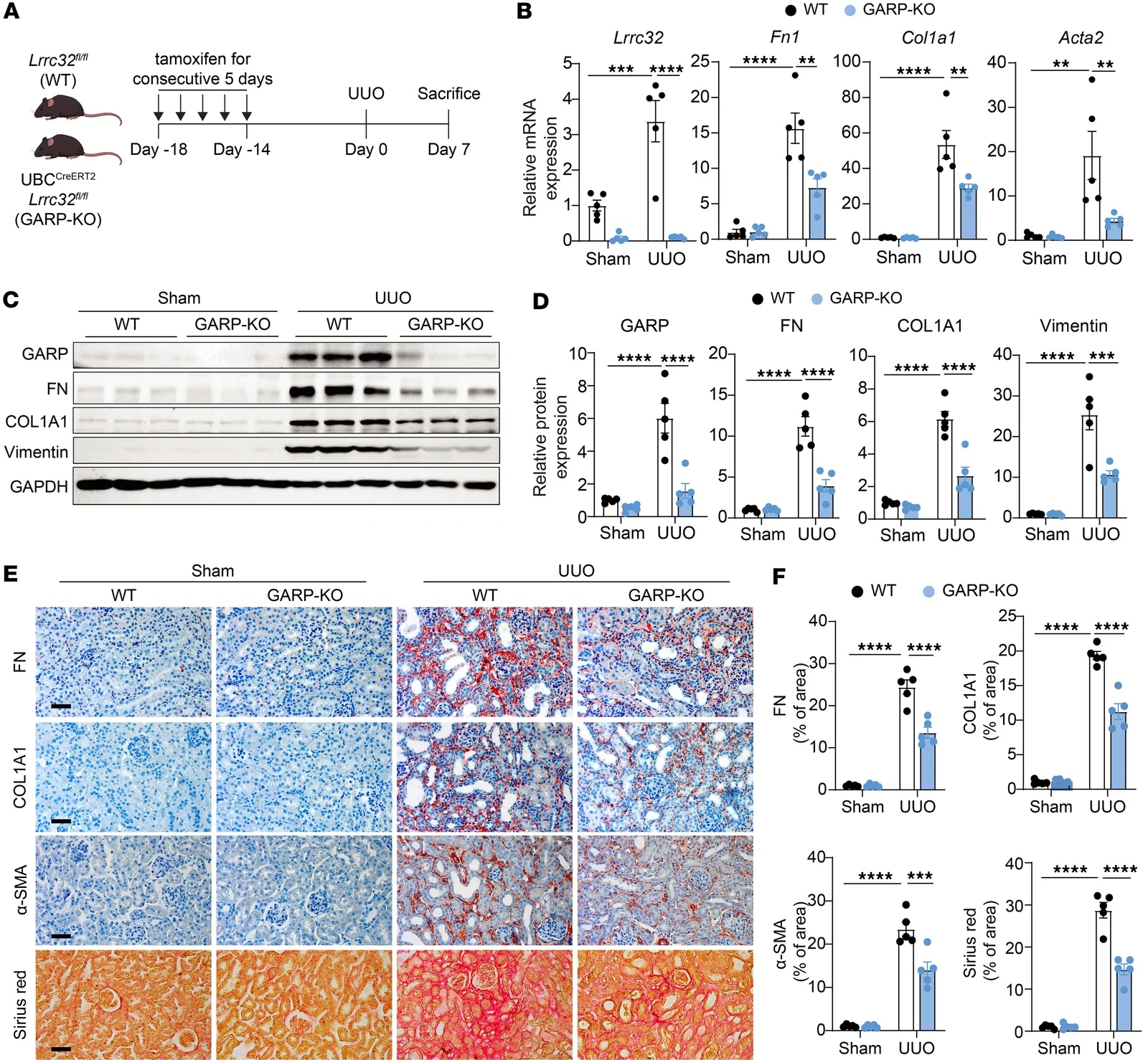
Global deletion of GARP mitigates UUO-induced kidney fibrosis. (**A**) Experimental design. (**B**) mRNA levels of *Lrrc32* and fibrotic markers (*Fn1*, *Col1a1*, and *Acta2*) in kidneys of WT and GARP-KO mice (*n* = 5 per group). (**C** and **D**) Representative (**C**) and quantification (**D**) of immunoblot for GARP, FN, COL1A1, and Vimentin in sham or UUO-treated WT and GARP-KO mice (*n* = 5 per group). Each lane represents 1 mouse. GAPDH was used as a loading control. (**E** and **F**) Representative staining (**E**) and quantitation (**F**) for FN, COL1A1 and α-SMA and Sirius red in kidneys of WT and GARP-KO mice. Scale bars: 50 μm. Data represent mean ± SEM. **P < 0.01; ***P < 0.001; ****P < 0.0001. Statistical significance was determined by 2-way ANOVA followed by Tukey’s multiple-comparison test (**B**, **D**, and **F**).

**Figure 3:**
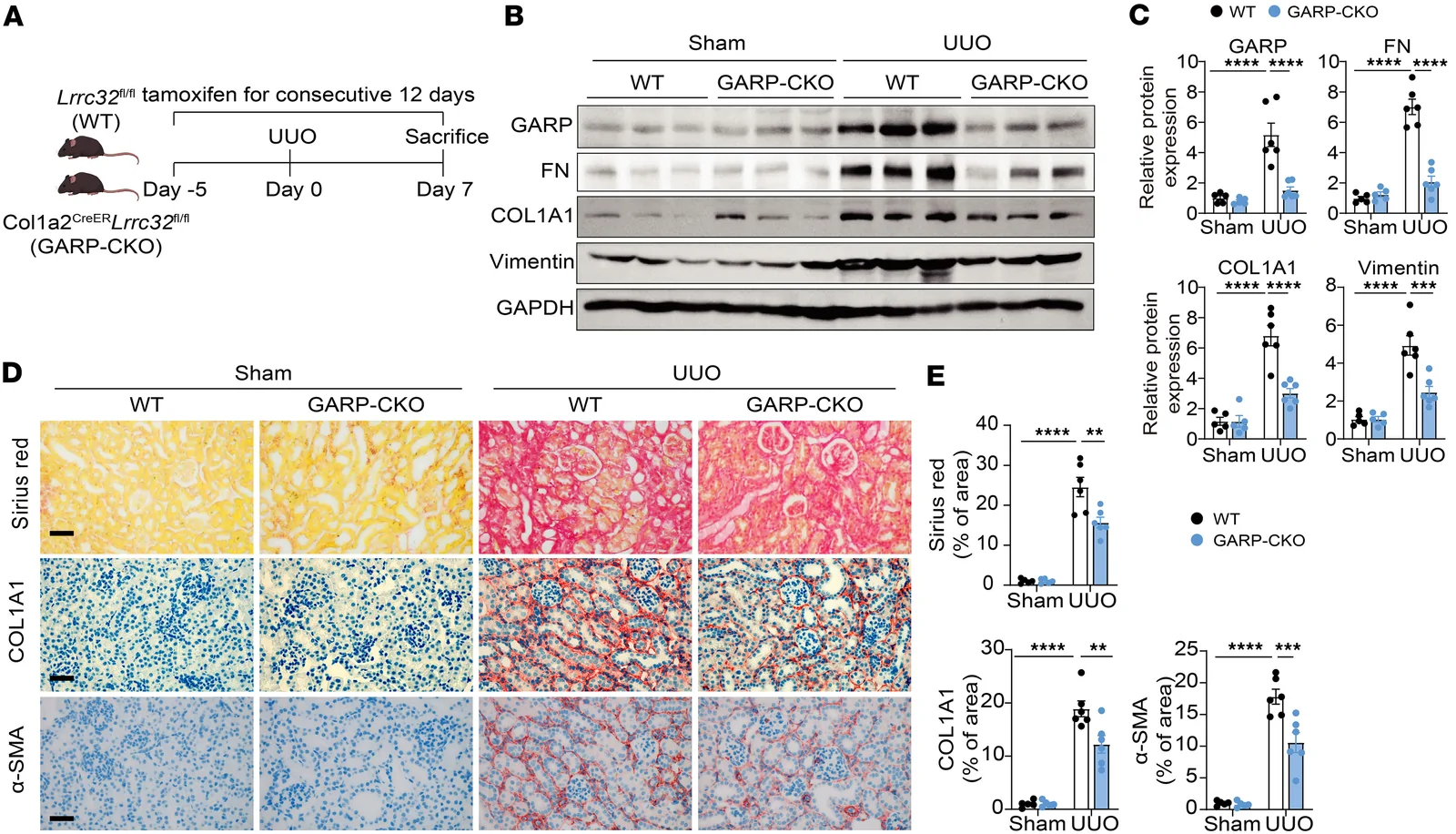
Fibroblast-specific deletion of GARP ameliorates UUO-induced kidney fibrosis in mice. (**A**) Schematic diagram of the experimental design. (**B** and **C**) Representative (**B**) and quantification (**C**) of immunoblot for GARP, FN, COL1A1, and Vimentin in WT and GARP-CKO mice (*n* = 5–6 per group). GAPDH was used as a loading control. (**D** and **E**) Representative staining (**D**) and quantification (**E**) for Sirius red, COL1A1, and α-SMA in WT and GARP-CKO mice (*n* = 6 per group). Scale bars: 50 μm. Data represent mean ± SEM. **P < 0.01; ***P < 0.001; ****P < 0.0001. Statistical significance was determined by 2-way ANOVA followed by Tukey’s multiple-comparison test (**C** and **E**).

**Figure 4:**
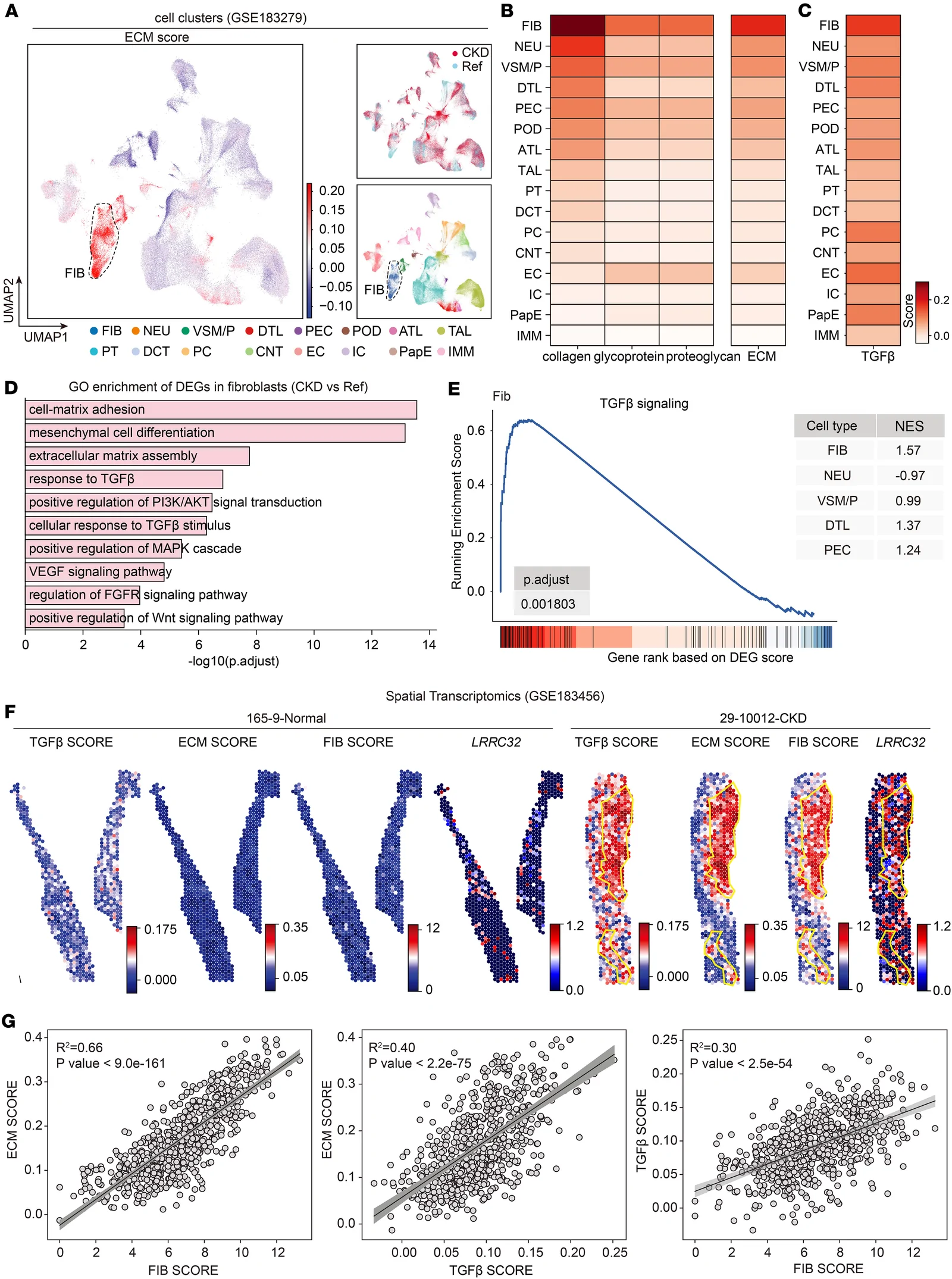
Fibroblasts and TGF-β signaling orchestrate fibrosis progression. (**A**) Uniform Manifold Approximation and Projection (UMAP) embeddings of the integrated sc/snRNA-seq dataset from CKD patients and control samples (GSE183279), colored by ECM score (left), group information (right top) and cell types (right bottom). FIB, fibroblast; NEU, neural cell; VSM/P, vascular smooth muscle cell or pericyte; DTL, descending thin limb cell; PEC, parietal epithelial cells; POD, podocyte; ATL, ascending thin limbs; TAL, thick ascending limb; PT, proximal tubule; DCT, distal convoluted tubule; PC, principal cell; CNT, connecting tubule; EC, endothelial cells; IC, intercalated cells; PapE, papillary tip epithelial cells abutting the calyx; IMM, immune cells. (**B**) Heatmap showing the collagen score, glycoprotein score, and proteoglycan score across cell types, which collectively form the ECM score. (**C**) Heatmap showing the TGF-β score across the cell types. (**D**) Bar plots of representative biological pathways enriched in fibroblasts defined by differentially expressed genes (DEGs). (**E**) Gene set enrichment analysis (GSEA) of fibroblasts for KEGG TGF-β signaling (left). The table (right) lists the normalized enrichment scores (NES) for the top 5 cell types ranked by ECM scores. (**F**) The TGF-β score, ECM score, Fibroblast score, and *LRRC32* expression in the 10× Visium data of control and CKD samples. Each score shares the same threshold in the color bar for comparison. (**G**) Pairwise correlations of TGF-β score, ECM score, and fibroblast score in CKD Visium data. *P* values were calculated using *F*-statistics from ordinary least-squares models, with the error band representing the 95% confidence interval.

**Figure 5:**
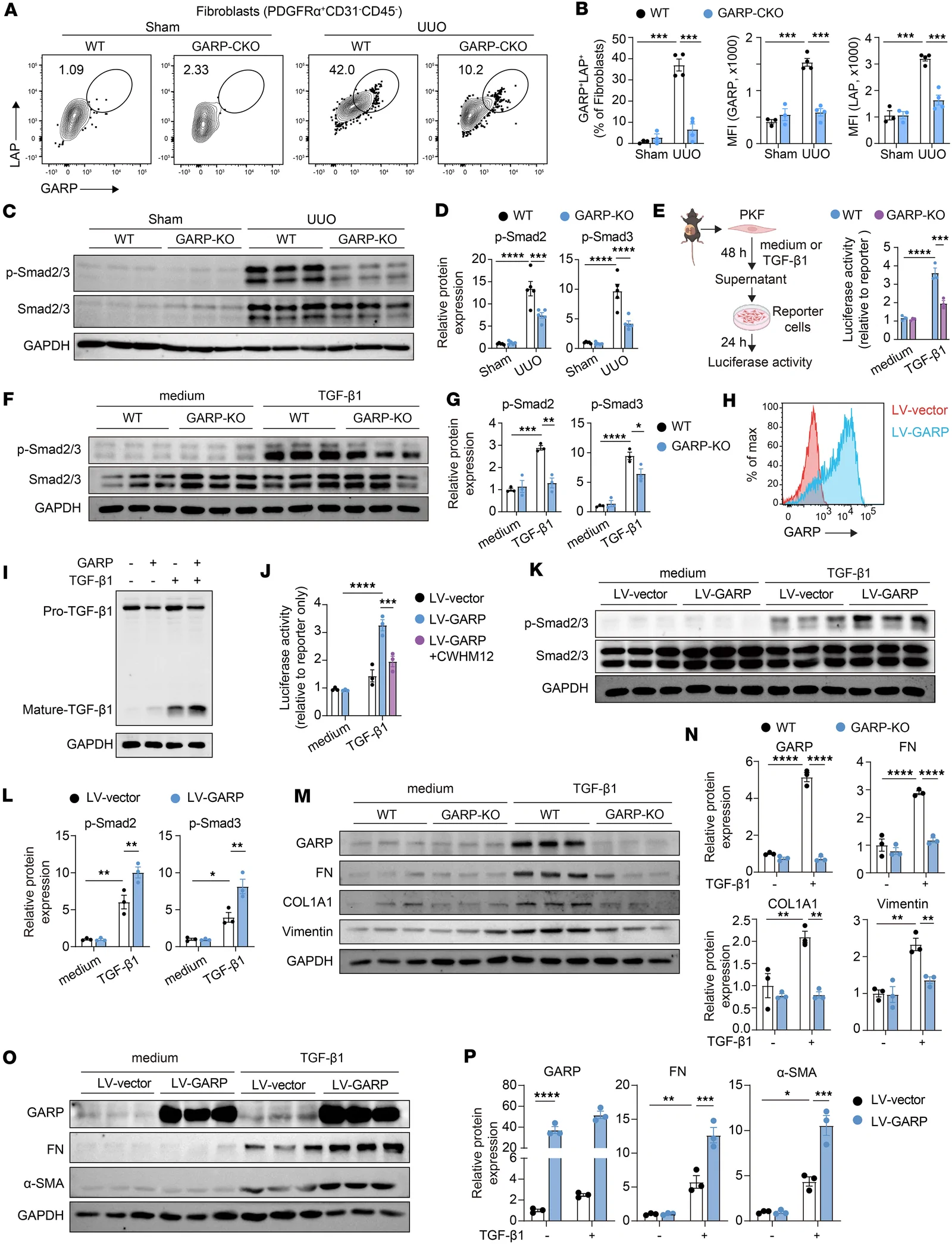
GARP-dependent TGF-β activation sustains Smad signaling and induces fibroblast stimulation. (**A** and **B**) Representative flow cytometry plots (**A**) and quantification (**B**) of GARP and LAP (TGF-β1) expression in kidney fibroblasts from sham or UUO-treated WT and GARP-KO mice (*n* = 3–4). MFI, mean fluorescence intensity. (**C** and **D**) Representative (**C**) and quantification (**D**) of immunoblot for p-Smad2/3 in sham or UUO-treated WT and GARP-KO mice (*n* = 5). (**E**) TGF-β reporter assay detecting active TGF-β released by PKFs with or without TGF-β1 priming. (**F** and **G**) Representative (**F**) and quantification (**G**) of immunoblot for p-Smad2/3 in medium only or TGF-β1–primed PKFs from WT and GARP-KO mice. (**H**) Flow cytometry of GARP expression in NRK-49F cells transduced with empty lentivirus (LV-vector) or GARP-overexpression lentivirus (LV-GARP). (**I**) Representative immunoblots showing TGF-β1 levels in cell lysates from medium (control) or TGF-β1–primed NRK-49F cells transduced with LV-vector or LV-GARP. (**J**) TGF-β reporter cell assay detecting active TGF-β release in NRK49F cells following TGF-β1 treatment in the absence or presence of CWHM-12 (an integrin αv inhibitor). (**K** and **L**) Representative (**K**) and quantification (**L**) of immunoblot for p-Smad2/3 in medium only or TGF-β1–primed NRK-49F cells. (**M** and **N**) Representative (**M**) and quantification (**N**) of immunoblot for GARP, FN, COL1A1, and Vimentin in medium only or TGF-β1–primed PKF cells from WT and GARP-KO mice. (**O** and **P**) Representative (**O**) and quantification (**P**) of immunoblot for GARP, FN, and α-SMA in medium only or TGF-β1–primed NRK-49F cells transduced with LV-vector or LV-GARP. Each lane in **C**, **F**, **I**, **K**, **M**, and **O** represents 1 well of cells. All in vitro experiments were independently repeated 3 times with similar results. Data represent mean ± SEM. *P < 0.05; **P < 0.01; ***P < 0.001; ****P < 0.0001. Statistical significance was determined by 2-way ANOVA followed by Tukey’s multiple-comparison test (**B**, **D**, **E**, **G**, **J**, **L**, **N**, and **P**).

**Figure 6:**
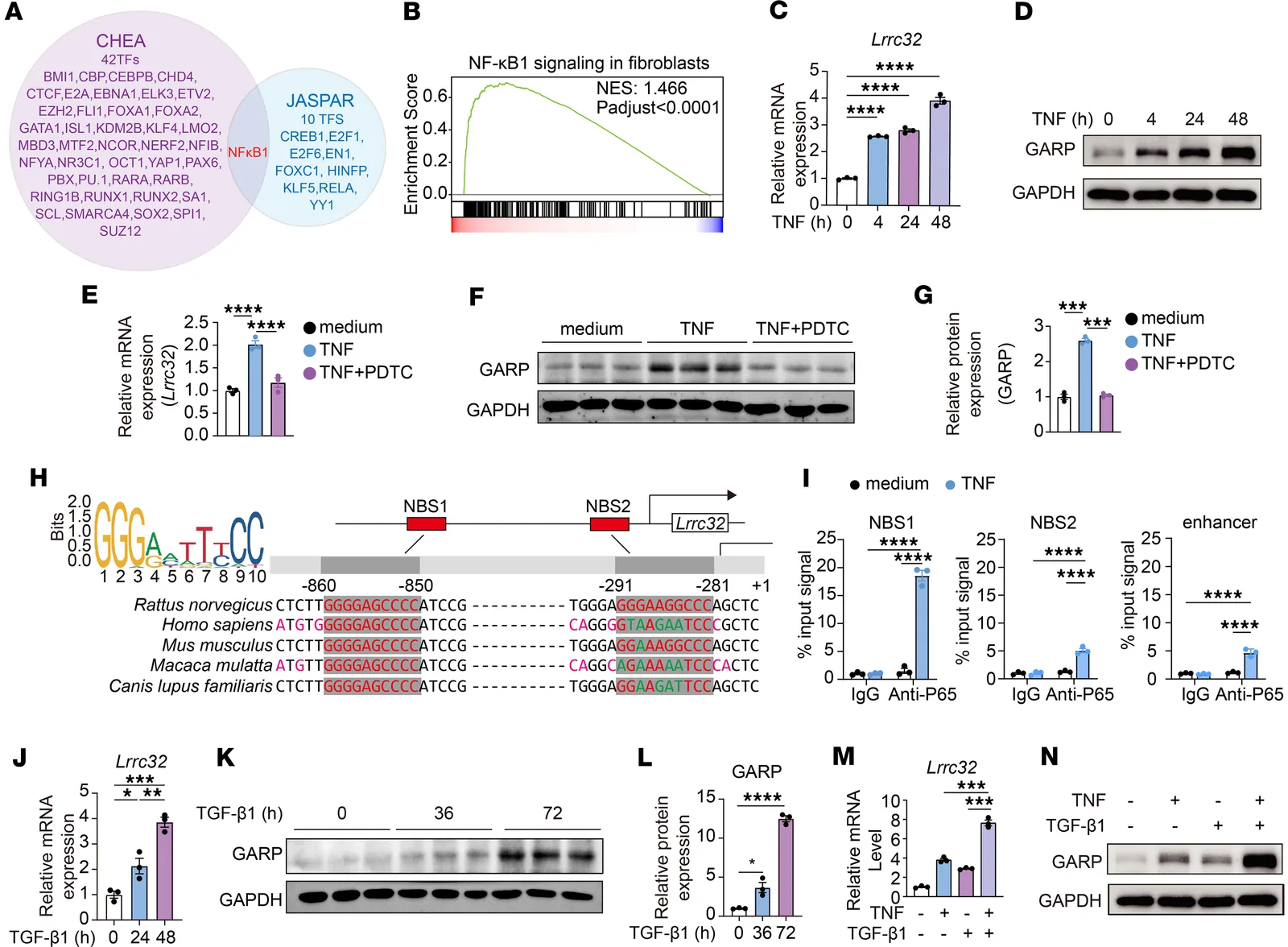
TNF upregulates GARP in an NF-κB–dependent fashion and synergizes with TGF-β1. (**A**) Venn diagram of transcription factors predicted to bind to the *Lrrc32* promoter region using ChEA and JASPAR tools. (**B**) Gene set enrichment analysis (GSEA) of fibroblasts from human kidney single-cell transcriptomic data (GSE183279) comparing CKD and reference samples, showing significant positive enrichment of the Hallmark TNFA signaling via NF-κB gene set in CKD fibroblasts. (**C** and **D**) mRNA (**C**) and protein (**D**) levels of GARP in NRK-49F cells treated with TNF (10 ng/mL) for the indicated durations. (**E**–**G**) mRNA (**E**) and protein (**F** and **G**) levels of GARP in TNF-stimulated NRK-49F cells in the absence or presence of PDTC (an NF-κB inhibitor). Representative images and quantitative analysis are shown in **E** and **F**, respectively. (**H**) The NF-κB consensus motif is shown at the top, and the gray regions mark 2 potential NF-κB binding sites (NBS1 and NBS2) in the rat *Lrrc32* promoter. (**I**) ChIP analysis of NF-κB p65 binding to NBS1 and NBS2 in the rat *Lrrc32* promoter and to the distal *Lrrc32* enhancer in NRK-49F cells under basal or TNF-stimulated conditions. Normal IgG served as a control. (**J**–**L**) mRNA (**J**) and protein (**K** and **L**) levels of GARP in NRK-49F cells treated with TGF-β1 (2 ng/mL) for the indicated durations. Representative images and quantitative analysis are shown in **J** and **K**, respectively. (**M** and **N**) mRNA (**M**) and protein (**N**) levels of GARP in NRK-49F cells treated with TNF, TGF-β1, or the combination of TGF-β1 and TNF. All in vitro experiments were independently repeated 3 times with similar results. Data represent mean ± SEM. *P < 0.05; **P < 0.01; ***P < 0.001; ****P < 0.0001. Statistical significance was determined by 1-way ANOVA (**C**, **E**, **G**, **J**, **L**, **M**) or 2-way ANOVA (**I**) with Tukey’s test for multiple comparisons.

**Figure 7:**
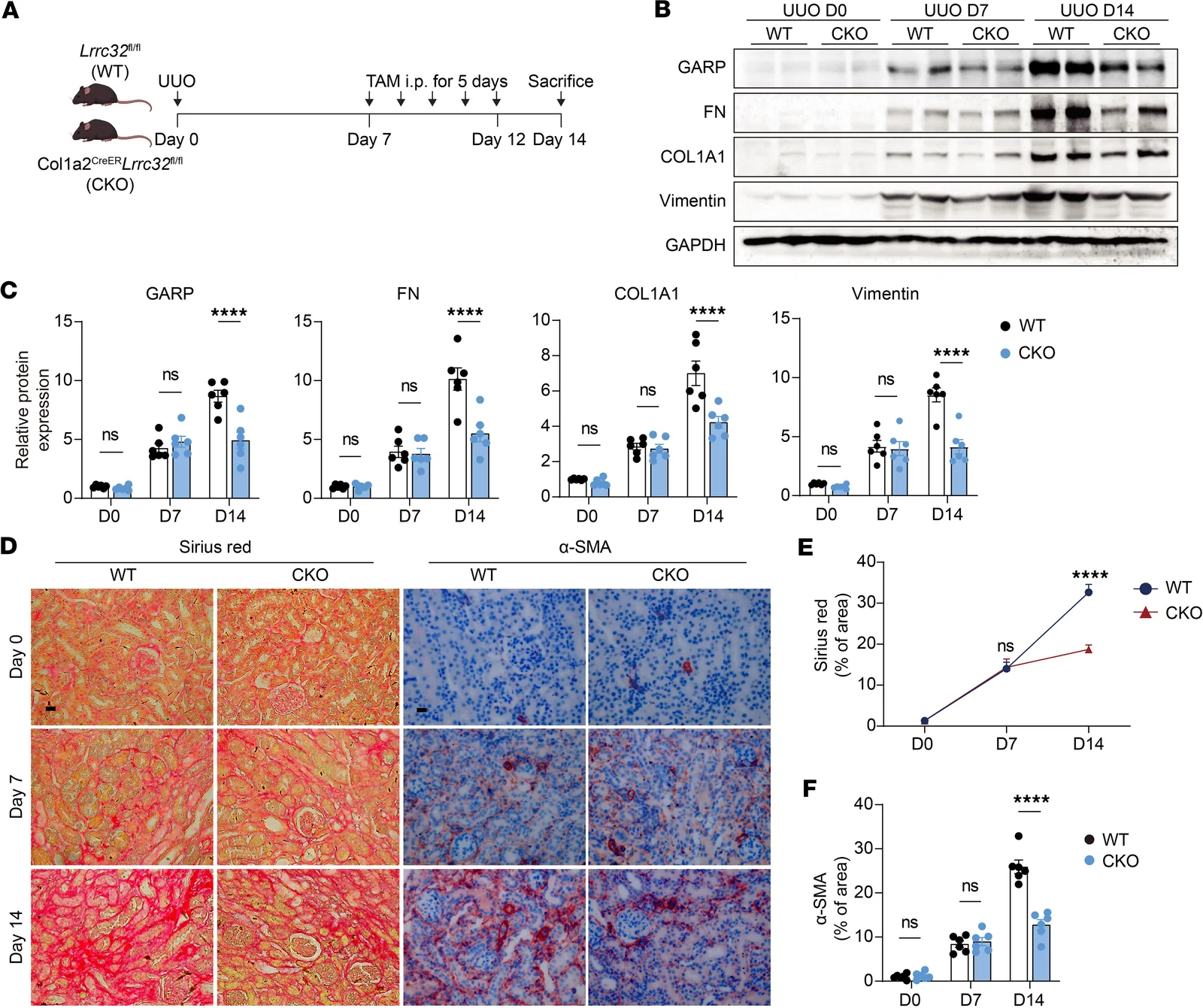
Therapeutic deletion of GARP in fibroblasts reduces established kidney fibrosis. (**A**) Experimental design illustrating the therapeutic targeting of GARP in fibroblasts after fibrosis establishment. (**B** and **C**) Representative immunoblots (**B**) and quantification (**C**) of GARP, fibronectin (FN), COL1A1, and Vimentin in kidneys from WT and GARP-CKO mice treated as in **A** (*n* = 5–6 per group). (**D**–**F**) Representative images (**D**) and quantitative analyses of Sirius red–stained fibrotic area (**E**) and α-SMA–positive area (**F**) in kidney sections from WT and GARP-CKO mice. Scale bars: 20 μm. Data represent mean ± SEM. ****P < 0.0001. NS, not significant (*P* > 0.05). Statistical significance was determined by 2-way ANOVA followed by Tukey’s multiple-comparison test (**C**, **E**, and **F**).

## Version 1 09/17/2026

In-Press Preview

## Version 2 10/22/2026

Electronic publication
