# Supplementary material for: Kidney fibrosis is mediated by GARP-restricted TGF-β activation in fibroblasts
Source: JCI Insight. 2026 Sep 17;11(20):e203039. doi: 10.1172/jci.insight.203039 (PMC13726566; doi:10.1172/jci.insight.203039)
Supplement: Supplementary file 1 [file jciinsight-11-203039-s022.pdf]

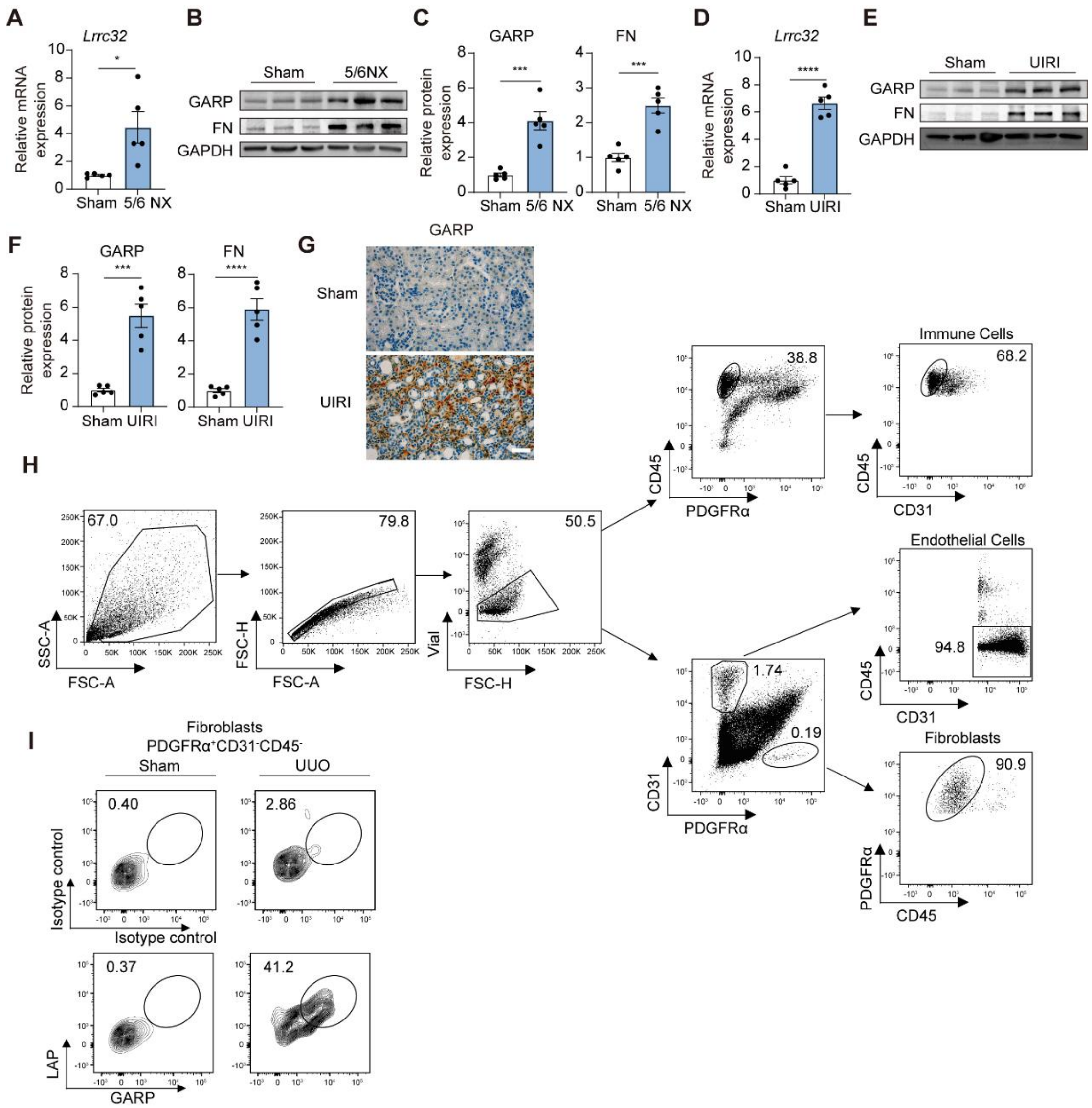

Supplemental Figure 1. GARP is upregulated in fibrotic kidneys of mice and rats. (**A**-**C**) mRNA (A) and protein (B-C) levels of GARP and FN in kidneys from sham or 5/6Nx mice (n = 5 per group). Representative immunoblot images (B) and quantification (C) are shown. (**D**-**F**) mRNA (D) and protein (E-F) levels of GARP and FN in kidneys from sham or unilateral ischemia-reperfusion injury (UIRI)-treated mice (n = 5 per group). Representative immunoblot images (E) and quantification (F) are shown. (**G**) Representative immunohistochemical staining for GARP in kidneys from sham or UIRI mice. Scale bar: 50μm. Images are representative of ≥5 mice per group. (**H**) Gating strategy used to identify kidney cell populations. Fibroblasts were defined as $PDGFR\alpha^{+}CD31^{-}CD45^{-}$cells, endothelial cells as $CD31^{+}CD45^{-}PDGFR\alpha^{-}$cells, and immune cells as $CD45^{+}CD31^{-}PDGFR\alpha^{-}$ cells. (**I**) Representative flow cytometry plots of isotype control and GARP/LAP staining of fibroblasts from sham and UUO kidneys. Data are presented as mean ± SEM, *P < 0.05; ***P < 0.001; ****P < 0.0001. Statistical significance was determined by unpaired two-tailed Student's t-test (A, C, D, F).

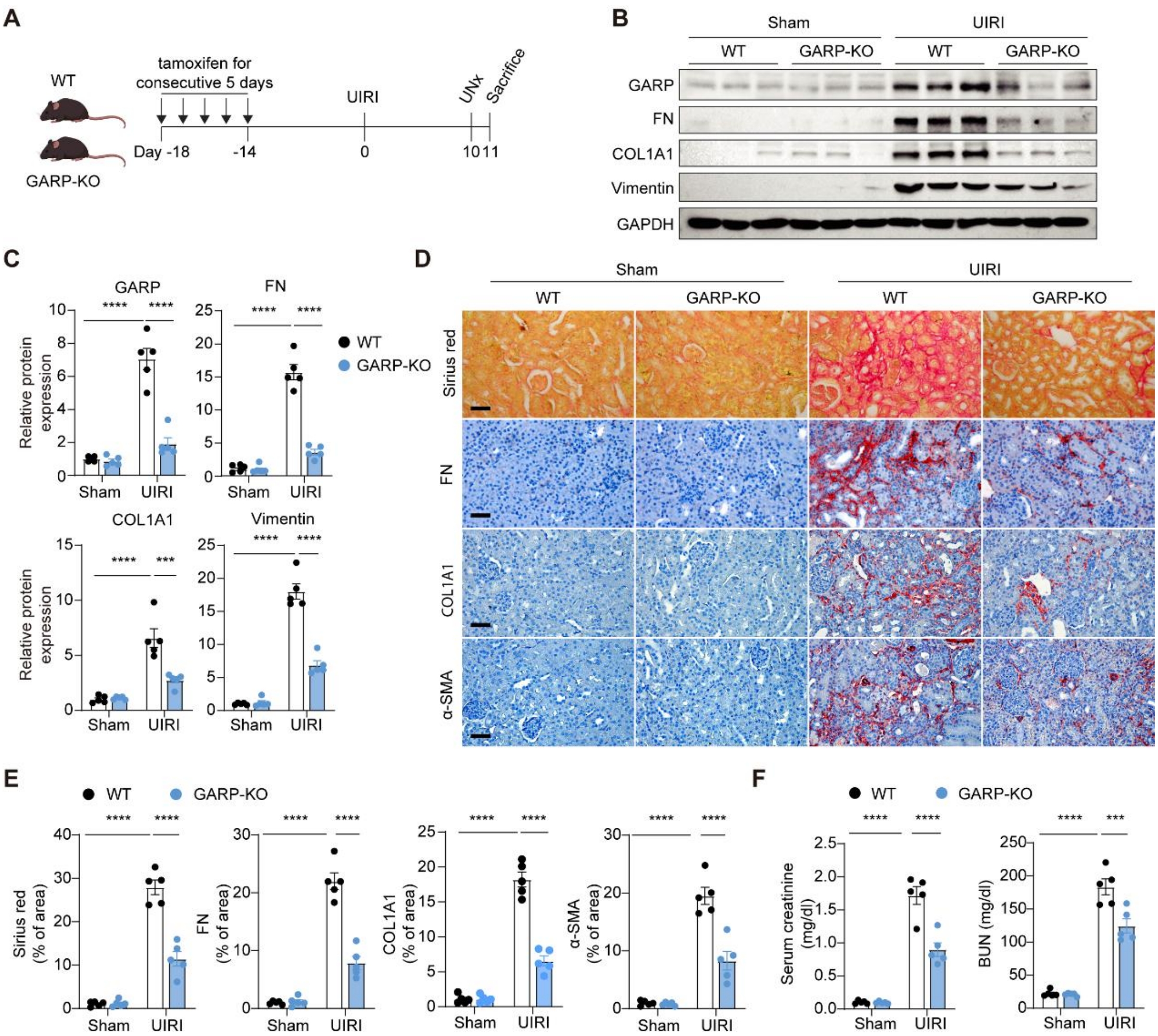

Supplemental Figure 2. Global deletion of GARP in mice mitigates kidney fibrosis induced by UIRI. (**A**) Experimental design. (**B**-**C**) Representative (B) and quantification (C) of immunoblot for GARP, FN, COL1A1, and Vimentin in sham or UIRI-treated WT and GARP-KO mice (n = 5 per group). GAPDH was used as a loading control. (**D**-**E**) Representative staining (D) and quantitation (E) for Sirius red, FN, COL1A1 and α-SMA in kidneys of WT and GARP-KO mice. Scale bar: 50 μm. (**F**) Levels of serum creatinine and blood urea nitrogen (BUN) from indicated mice. Data represent means ± SEM. ***$P < 0.001$; ****$P < 0.0001$. Statistical significance was determined by two-way ANOVA followed by Tukey's multiple-comparison test (C, E, F).

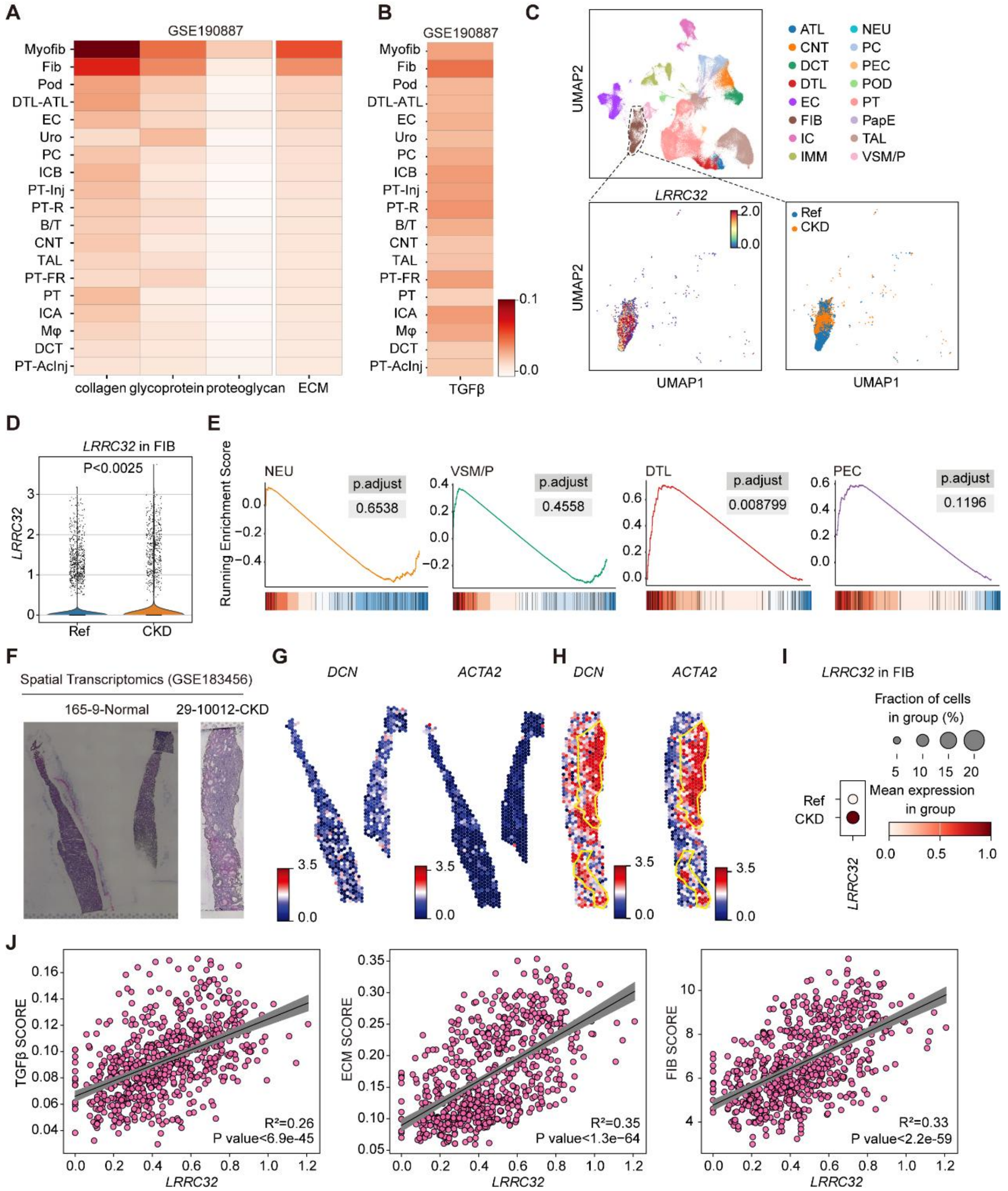

Supplemental Figure 3. Fibroblasts and TGF-β signaling orchestrate fibrosis progression. (**A**) Heatmap showing the collagen score, glycoprotein score and proteoglycan score across cell types from mouse kidney (GSE190887), which collectively form the ECM score. Myofib, myofibroblast; Fib, fibroblast; Pod, podocyte; DTL-ATL, descending/ascending thin limb cell; EC, endothelial cell; Uro, urothelium; PC, principal cell of collecting duct; ICB, type B intercalated cell of collecting duct; PT-Inj, injured PT; PT-R, repairing PT; B/T, immune cell; CNT, connecting tubule; TAL, thick ascending limb; PT-FR, failed repair PT cell; PT, proximal tubule; ICA, type A intercalated cell of collecting duct; Mφ, macrophage; DCT, distal convoluted tubule; PT-AcInj, acute injury PT. (**B**) Heatmap showing the TGF-β score across the cell types, sharing the same y-axis labels with Panel (A). (**C**) UMAP of fibroblasts from the human dataset (GSE183279) (upper panel), colored by group (Ref vs CKD; lower panel, right)

and by *LRRC32* expression (left). Ref, reference group (control). (**D**) Violin plot showing *LRRC32* expression in fibroblasts from Ref and CKD kidneys. (**E**) GSEA enrichment of NEU, VSM/P, DTL and PEC for KEGG TGF-β signaling. NEU, Neural cells; VSM/P, vascular smooth muscle cell or pericyte; DTL, descending/ascending thin limb cell; PEC, Parietal Epithelial Cell. (**F**) H&E staining images of representative control and CKD samples from GSE183456. (**G**-**H**) Gene expression levels of *DCN* and *ACTA2* in the control (G) and CKD (H) samples. (**I**) Dot plot summarizing, within fibroblasts, the fraction of cells expressing *LRRC32* and the mean *LRRC32* expression in the Ref and CKD groups. (**J**) Scatter plots showing associations between *LRRC32* expression and TGF-β score, ECM score, and fibroblast score in fibroblasts.

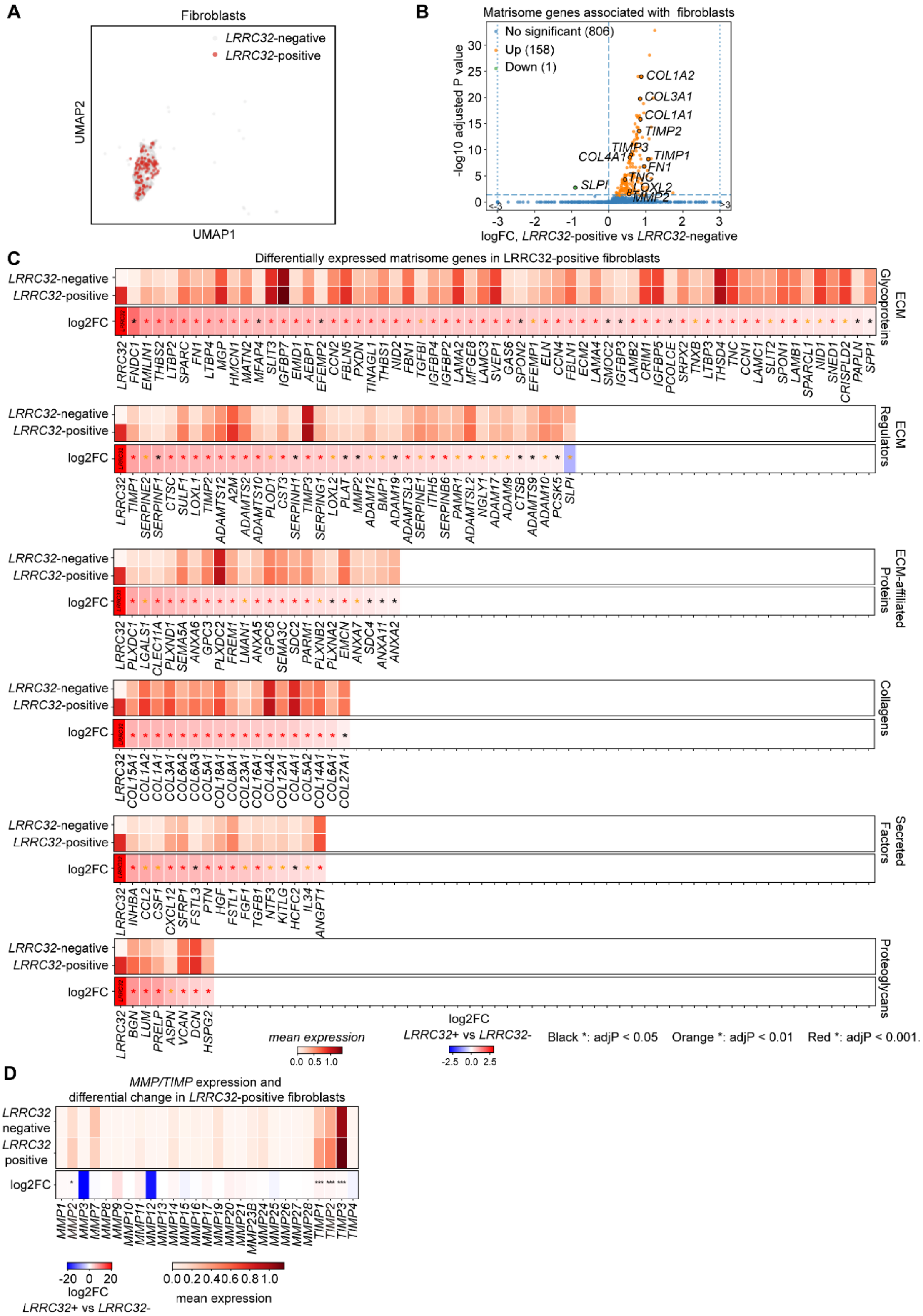

Supplemental Figure 4. Matrisome features associated with *LRRC32*-positive fibroblasts in human kidneys. (**A**) UMAP visualization of fibroblasts from the human kidney sc/snRNA-seq dataset, highlighting *LRRC32*-positive and *LRRC32*-negative fibroblasts. (**B**) Volcano plot showing differentially expressed matrisome genes in *LRRC32*-positive fibroblasts compared with *LRRC32*-negative fibroblasts. Upregulated and downregulated genes are indicated. Representative matrisome genes are labeled.

(**C**) Heatmap showing mean expression and differential changes of differentially expressed matrisome genes in *LRRC32*-positive fibroblasts, grouped by matrisome categories, including ECM glycoproteins, ECM regulators, ECM-affiliated proteins, collagens, secreted factors, and proteoglycans. The top rows show mean expression in *LRRC32*-negative and *LRRC32*-positive fibroblasts, and the bottom row shows log2 fold change. Asterisks indicate adjusted P value thresholds. (**D**) Heatmap showing expression and differential changes of MMP and TIMP family genes in *LRRC32*-positive fibroblasts compared with *LRRC32*-negative fibroblasts.

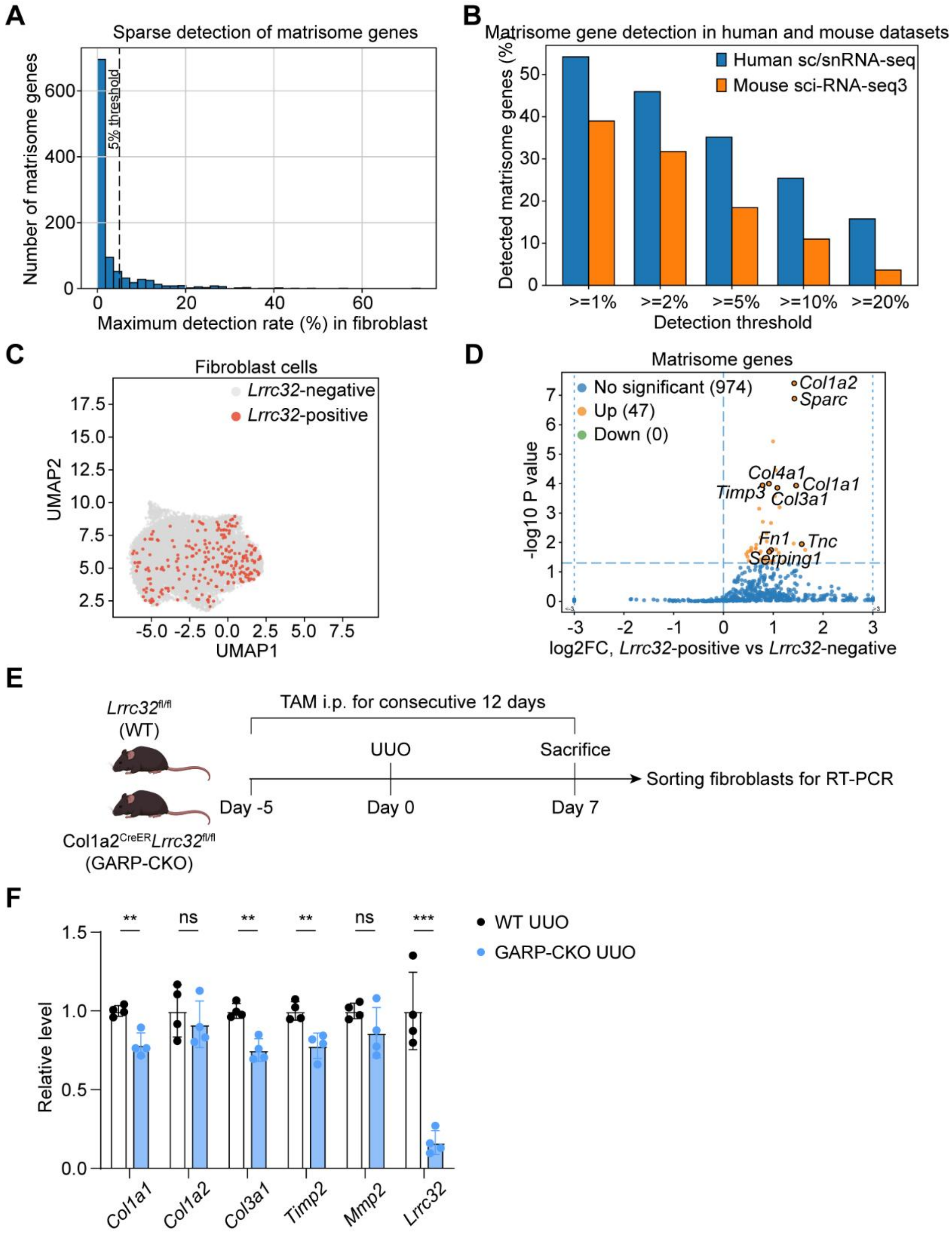

Supplemental Figure 5. Matrisome analysis in mouse fibroblasts. (**A**) Histogram showing sparse detection of matrisome genes in fibroblasts from the mouse scRNA-seq dataset. The dashed line indicates the number of matrisome genes detected above the indicated detection threshold. (**B**) Bar plot comparing matrisome gene detection rates between the human sc/snRNA-seq dataset and the mouse sci-RNA-seq3 dataset across different detection thresholds. (**C**) UMAP visualization of mouse fibroblasts, highlighting *Lrrc32*-positive and *Lrrc32*-negative fibroblasts. (**D**) Volcano plot showing matrisome genes differentially expressed between *Lrrc32*-positive and *Lrrc32*-negative mouse fibroblasts. Representative matrisome genes are labeled. (**E**) Experimental

design for isolating PDGFRα$^+$ fibroblasts from WT and fibroblast-specific GARP-CKO mice after UUO for RT-PCR analysis. (**F**) RT-PCR analysis of selected fibrotic ECM and matrix-remodeling genes in sorted fibroblasts from WT-UUO and GARP-CKO-UUO mice (n = 4 per group). Data represent mean ± SEM. ns, nonsignificant; **P < 0.01; ***P < 0.001. Statistical significance was determined by unpaired two-tailed Student's t-test (F).

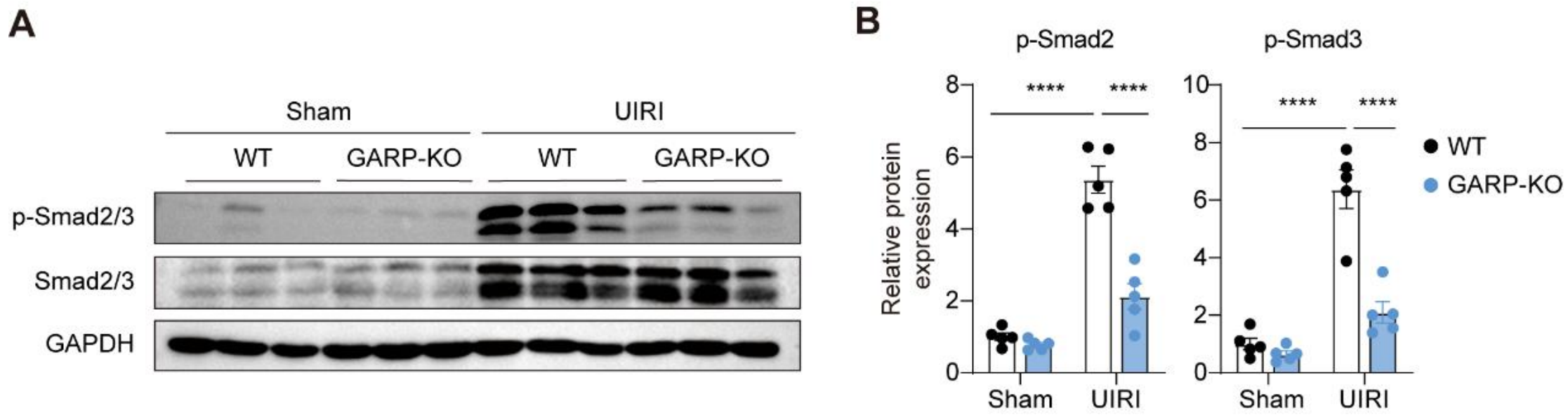

Supplemental Figure 6. GARP-dependent TGF-β activation sustains signaling in kidney fibroblasts. (**A-B**) Representative immunoblot (A) and quantification (B) of p-Smad2/3 in kidneys from WT and GARP-KO mice on day 11 post-UIRI (n = 5 per group). Data represent means ± SEM. ****P < 0.0001. Statistical significance was determined by two-way ANOVA followed by Tukey's multiple-comparison test (B).

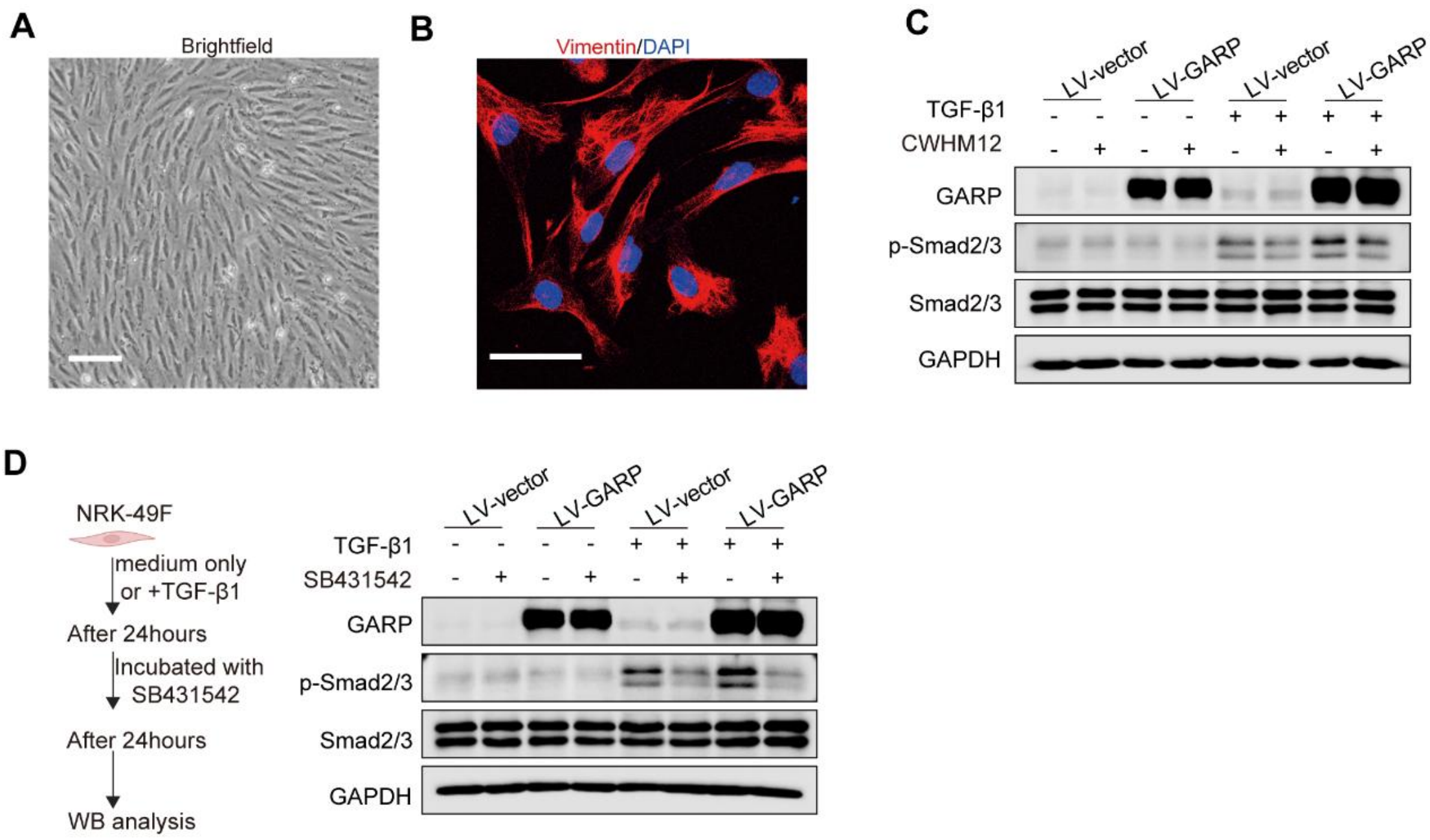

Supplemental Figure 7. Characterization of isolated mouse primary kidney fibroblast (PKF) cells. (**A**-**B**) Brightfield (A) and immunofluorescence images of Vimentin (fibroblast marker) (B) showing the morphology of isolated PKFs. DAPI indicates nuclear staining. Scale bars: 100 μm (A) and 50 μm (B). (**C**-**D**) Representative immunoblots showing the effects of CWHM12 (C), an integrin αv inhibitor, and SB431542 (D), a TGF-β receptor I inhibitor, on GARP expression and Smad2/3 phosphorylation in LV-vector or LV-GARP NRK-49F cells under basal conditions or following TGF-β1 stimulation. Total Smad2/3 and GAPDH were used as loading controls. Experimental design for SB431542 treatment is shown in the left panel of (D). Data in C and D are representative of at least three independent experiments.

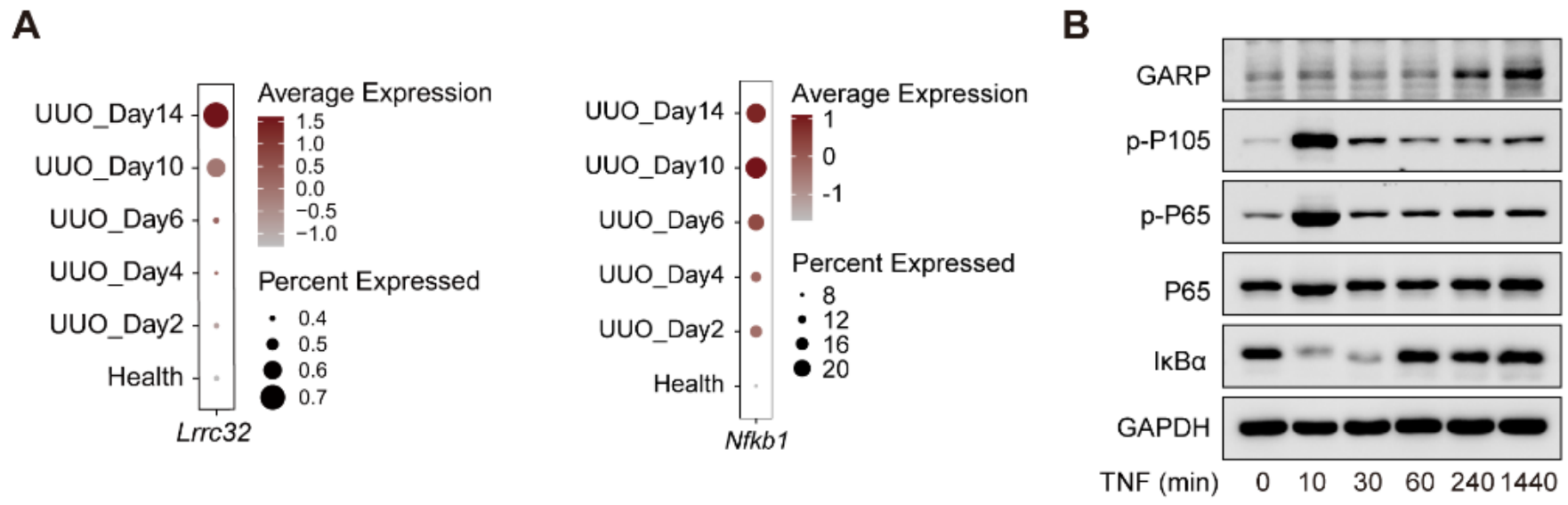

Supplemental Figure 8. Temporal association between NF-κB activation and GARP expression. (**A**) Dot plots showing the expression of *Lrrc32* (left) and *Nfkb1* (right) across fibroblasts from healthy kidneys and UUO kidneys at different time points. (**B**) Representative immunoblot showing time-dependent activation of NF-κB signaling and GARP induction in NRK-49F cells following TNF treatment. p-P105, p-P65, total P65, IκBα, GARP, and GAPDH are shown. GAPDH was used as a loading control. Data are representative of two independent experiments.

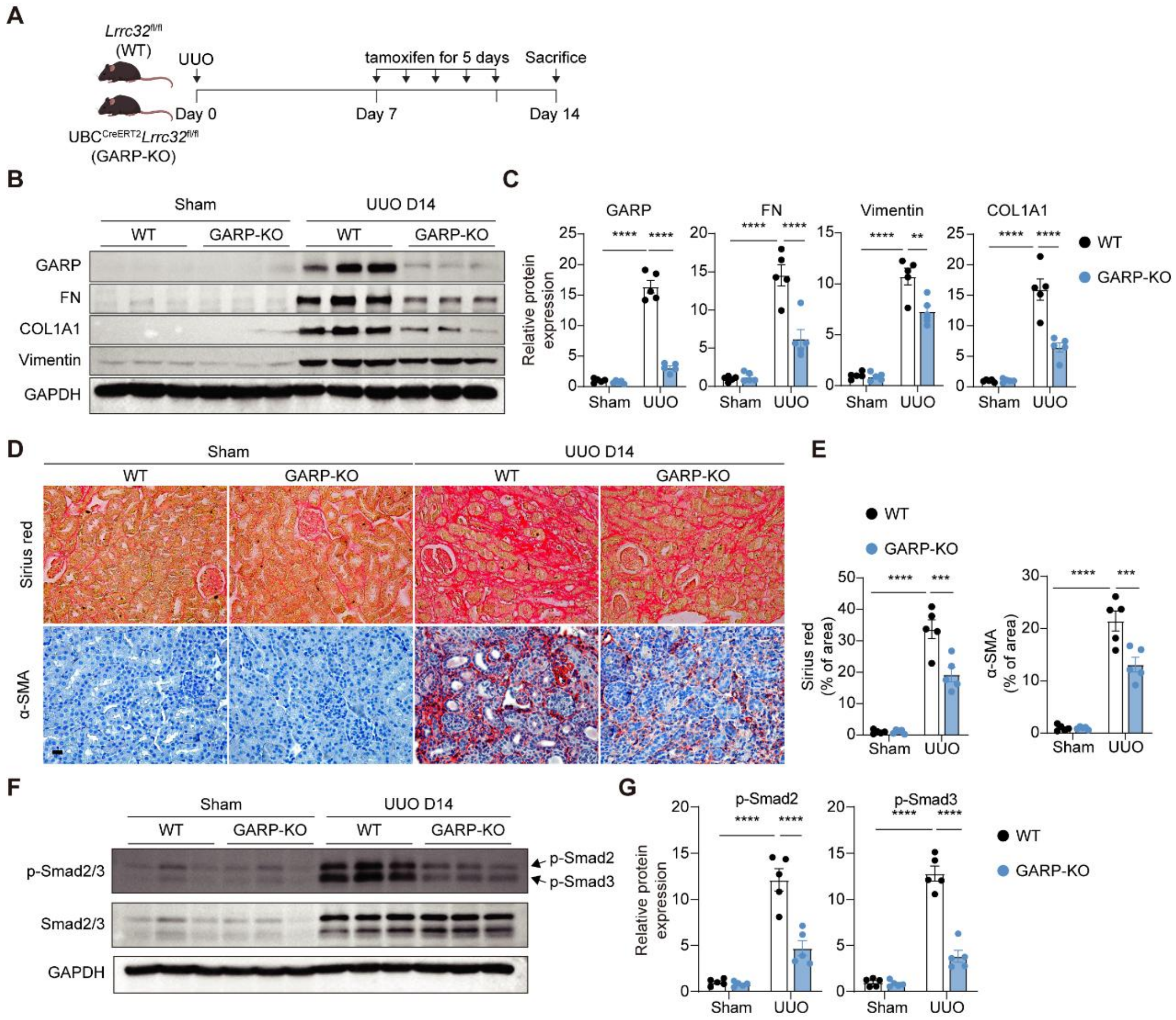

Supplemental Figure 9. Therapeutic deletion of GARP reduces established kidney fibrosis. (**A**) Experimental design illustrating the therapeutic deletion of GARP after fibrosis establishment. (**B**-**C**) Representative immunoblots (B) and quantification (C) of GARP, FN, COL1A1, and Vimentin in kidneys from WT and GARP-KO mice treated as in (A) (n = 5 per group). ns, not significant. (**D-E**) Representative images (D) and quantitative analyses of Sirius red-stained fibrotic area (E) and α-SMA-positive area (E) in kidney sections from WT and GARP-KO mice. (**F**-**G**) Representative immunoblots (F) and quantification (G) of p-Smad2/3 in kidneys from WT and GARP-KO mice treated as in (A). Data represent means ± SEM. **P < 0.01; ***P < 0.001; ****P < 0.0001. Statistical significance was determined by two-way ANOVA followed by Tukey's multiple-comparison test (C, E, G).
