# Supplementary material for: Kidney fibrosis is mediated by GARP-restricted TGF-β activation in fibroblasts
Source: JCI Insight. 2026 Sep 17;11(20):e203039. doi: 10.1172/jci.insight.203039 (PMC13726566; doi:10.1172/jci.insight.203039)

Unedited blot and gel images

Figure 1 D

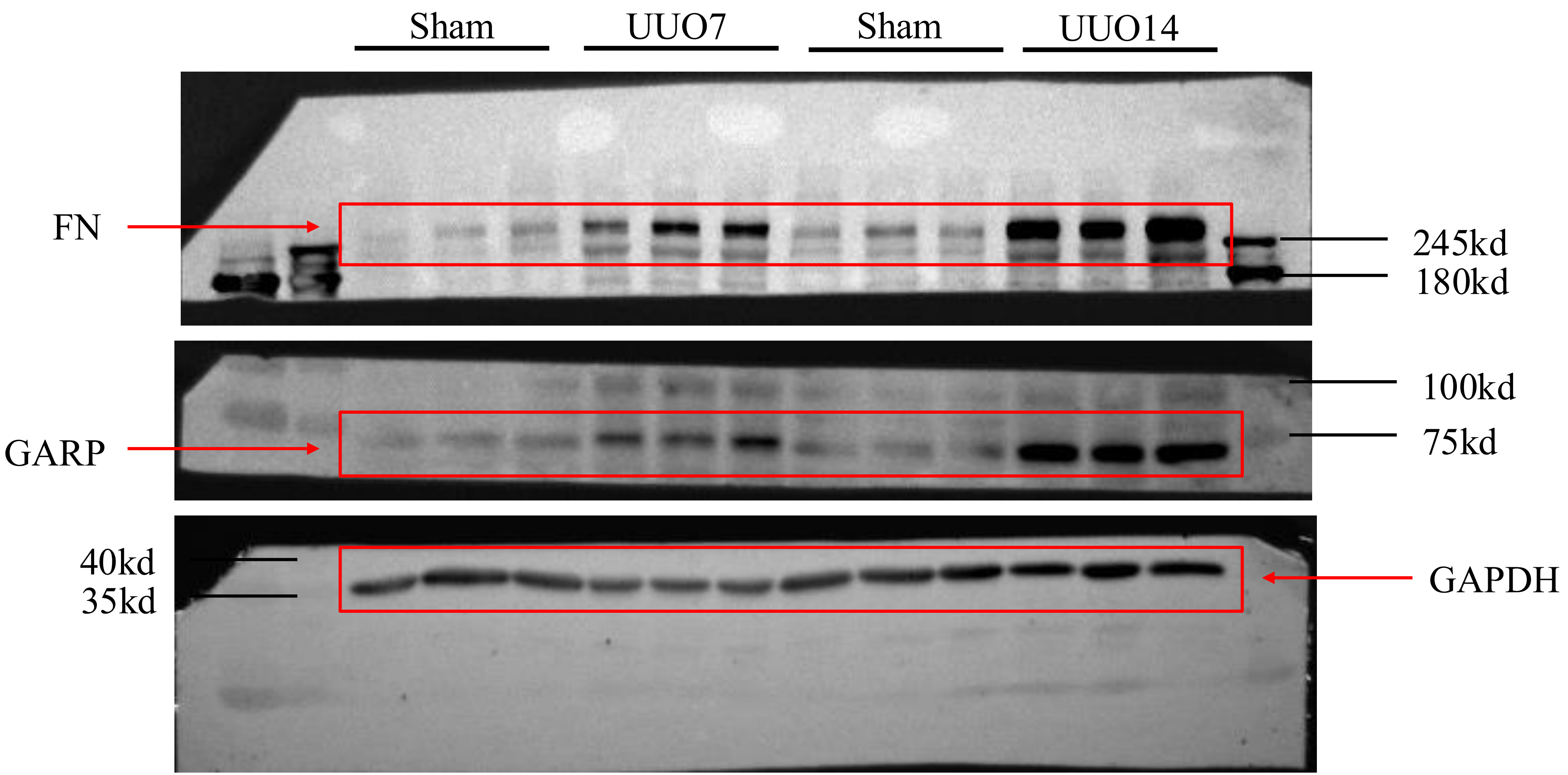

Sham
UUO7
Sham
UUO14
FN
245kd
180kd
100kd
GARP
75kd
40kd
35kd
GAPDH

Figure 2 C

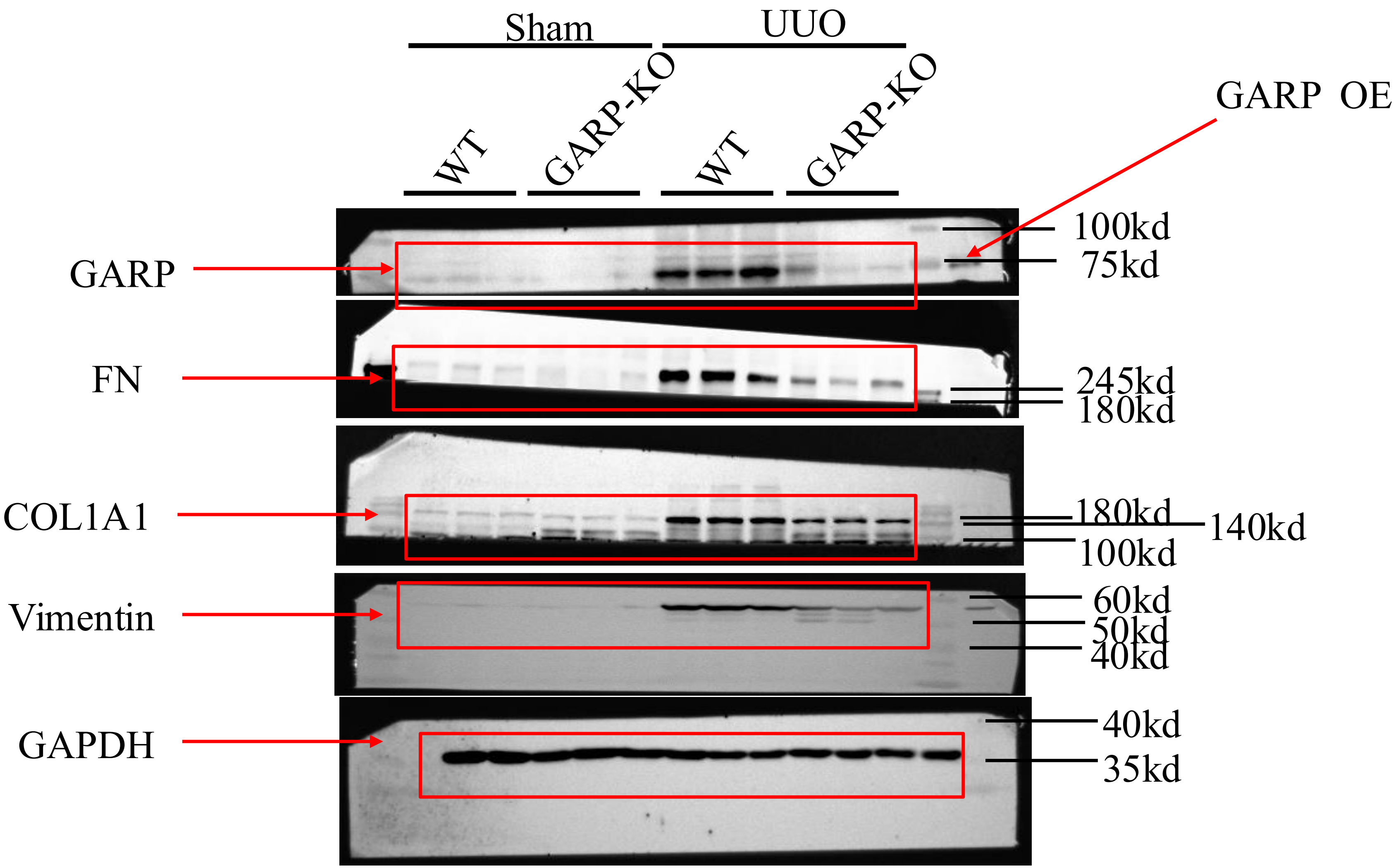

Sham
UUO
WT
GARP-KO
WT
GARP-KO
GARP OE
GARP
FN
COL1A1
Vimentin
GAPDH
100kd
75kd
245kd
180kd
180kd
140kd
100kd
60kd
50kd
40kd
40kd
35kd

Figure 3 B

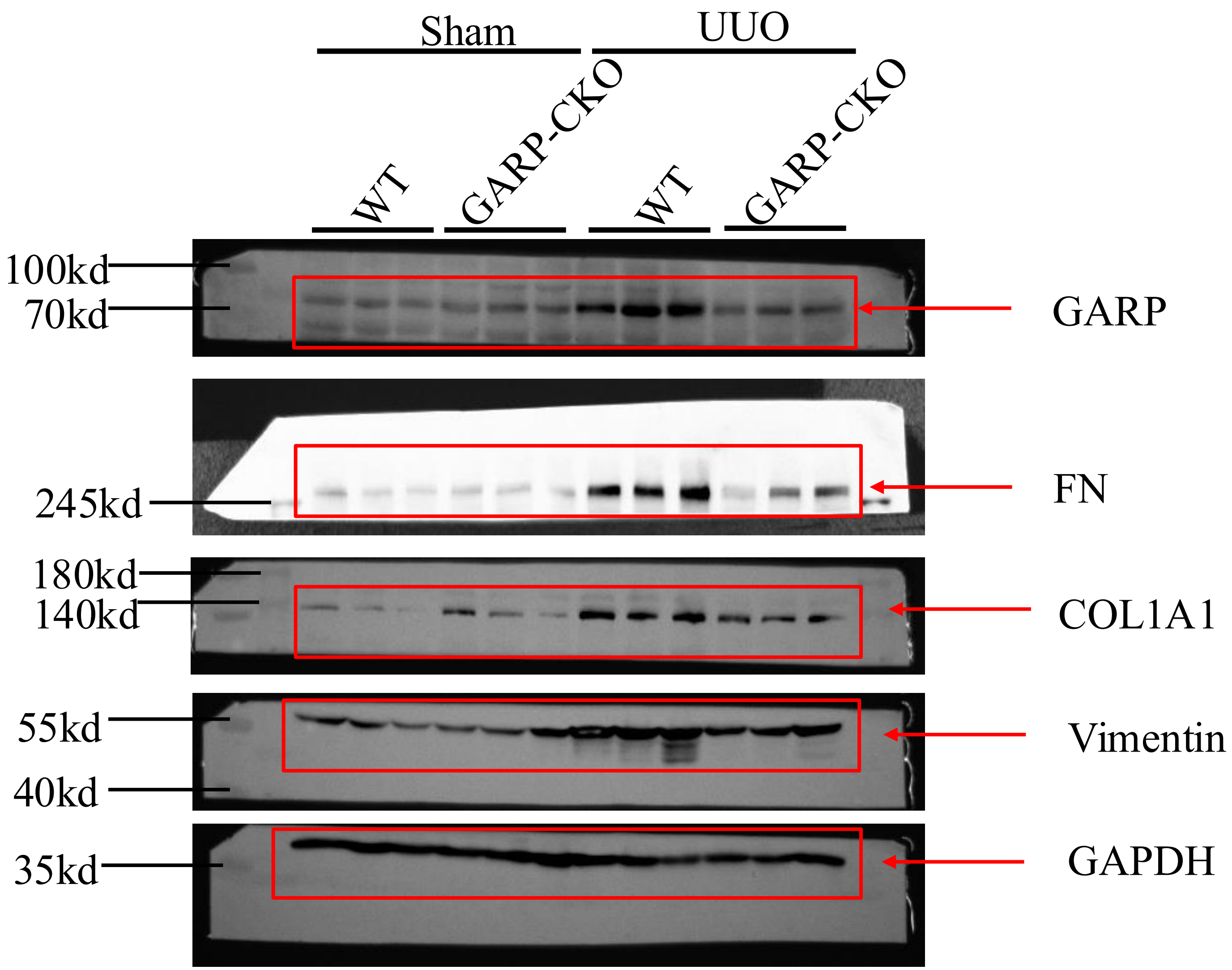

Sham
UUO
WT
GARP-CKO
WT
GARP-CKO
100kd
70kd
GARP
245kd
FN
180kd
140kd
COL1A1
55kd
40kd
Vimentin
35kd
GAPDH

Figure 5 C

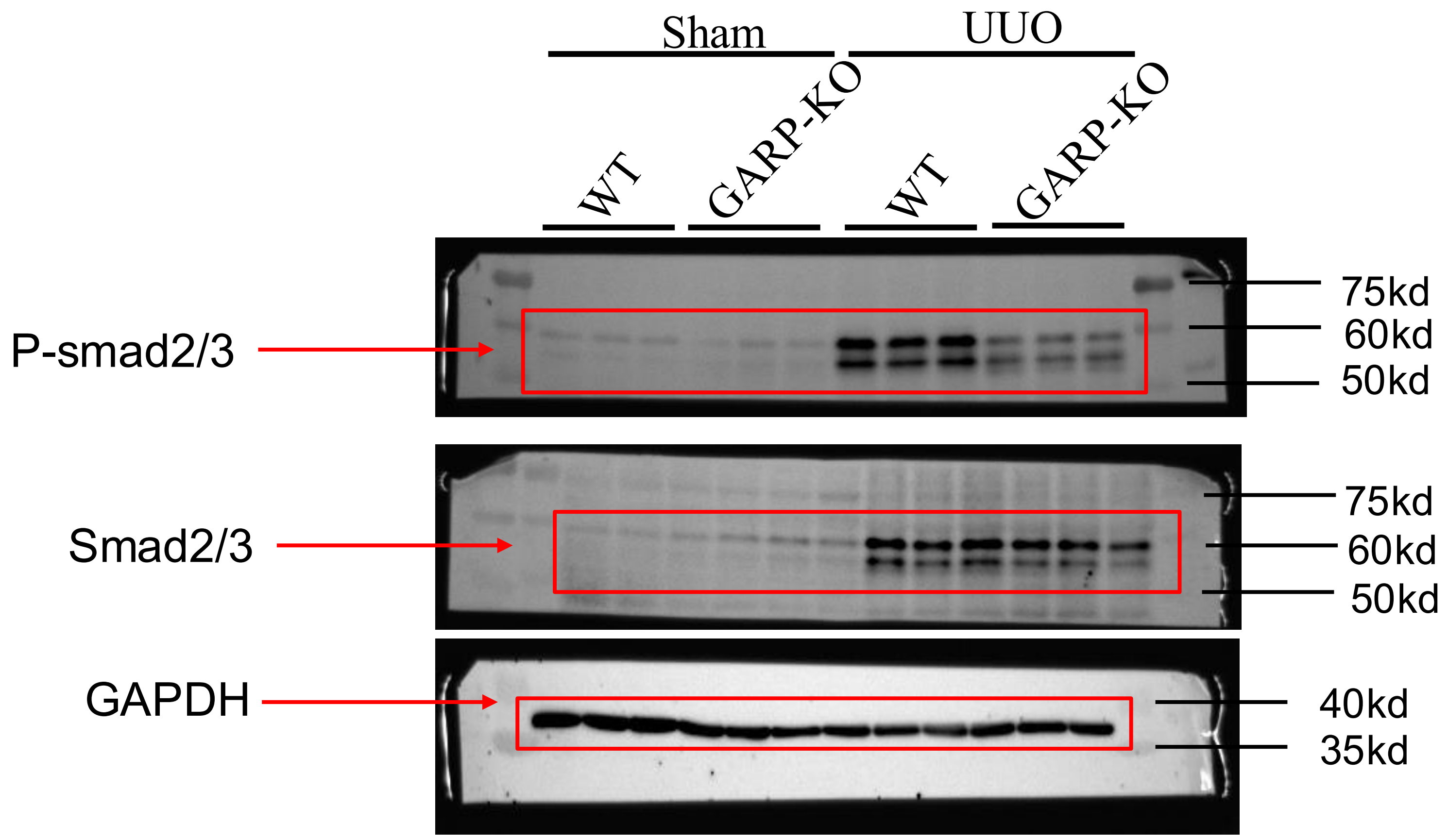

Sham
UUO
WT
GARP-KO
WT
GARP-KO
P-smad2/3
75kd
60kd
50kd
Smad2/3
75kd
60kd
50kd
GAPDH
40kd
35kd

Figure 5 F

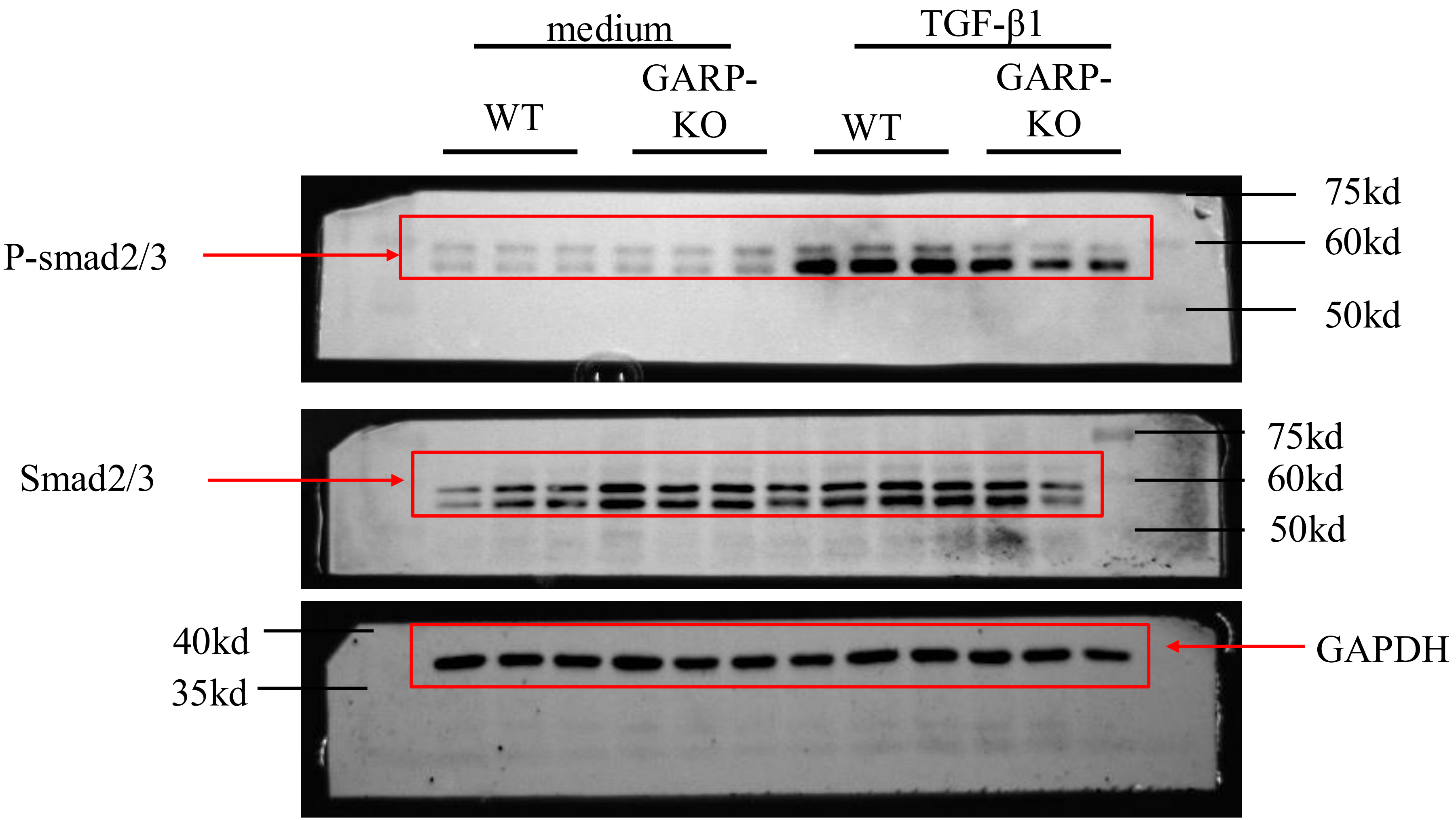

medium
TGF-β1
WT
GARP-KO
WT
GARP-KO
P-smad2/3
75kd
60kd
50kd
Smad2/3
75kd
60kd
50kd
40kd
35kd
GAPDH

Figure 5 I

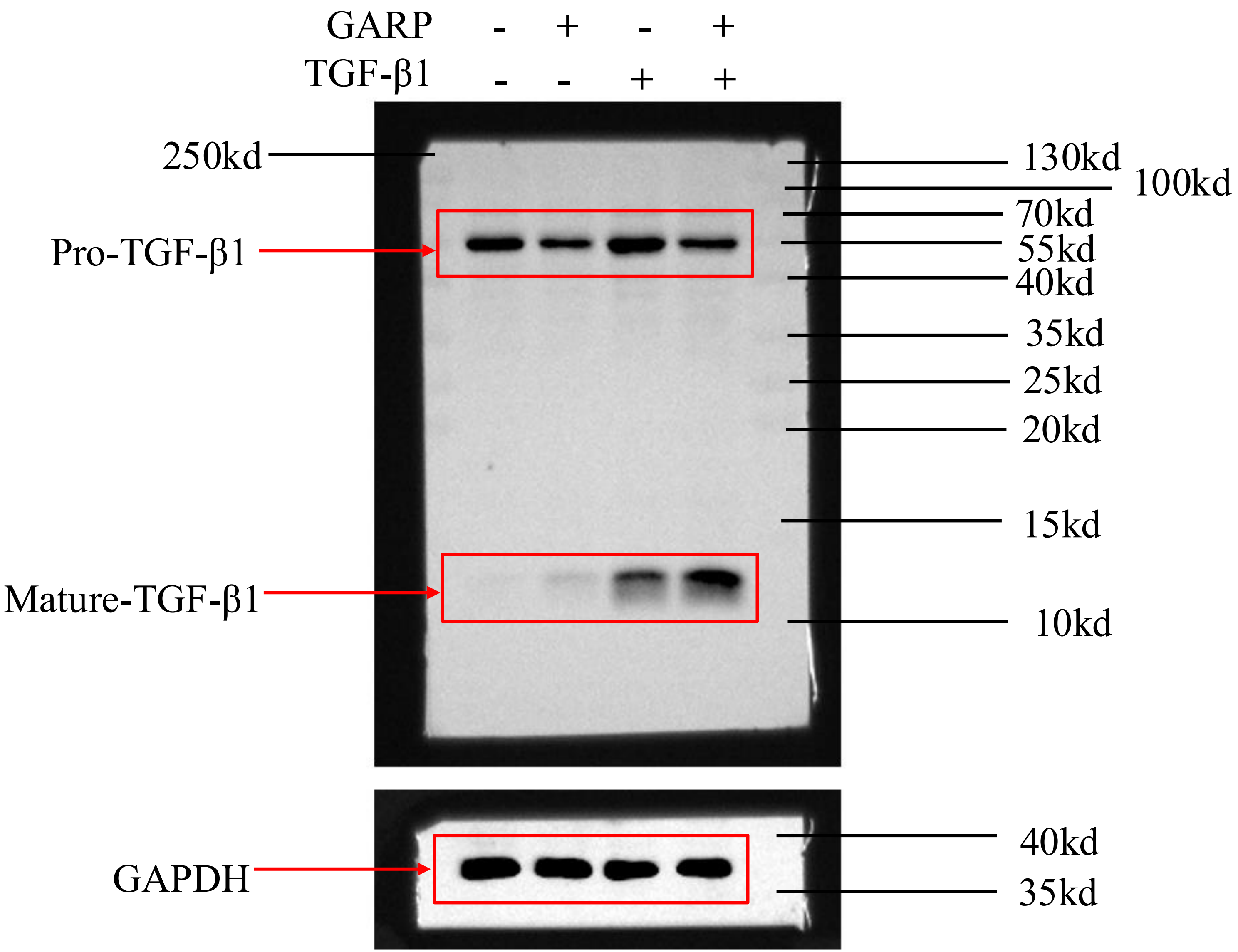

GARP - + - +
TGF-β1 - - + +
250kd
Pro-TGF-β1
Mature-TGF-β1
GAPDH
130kd
100kd
70kd
55kd
40kd
35kd
25kd
20kd
15kd
10kd
40kd
35kd

Figure 5 K

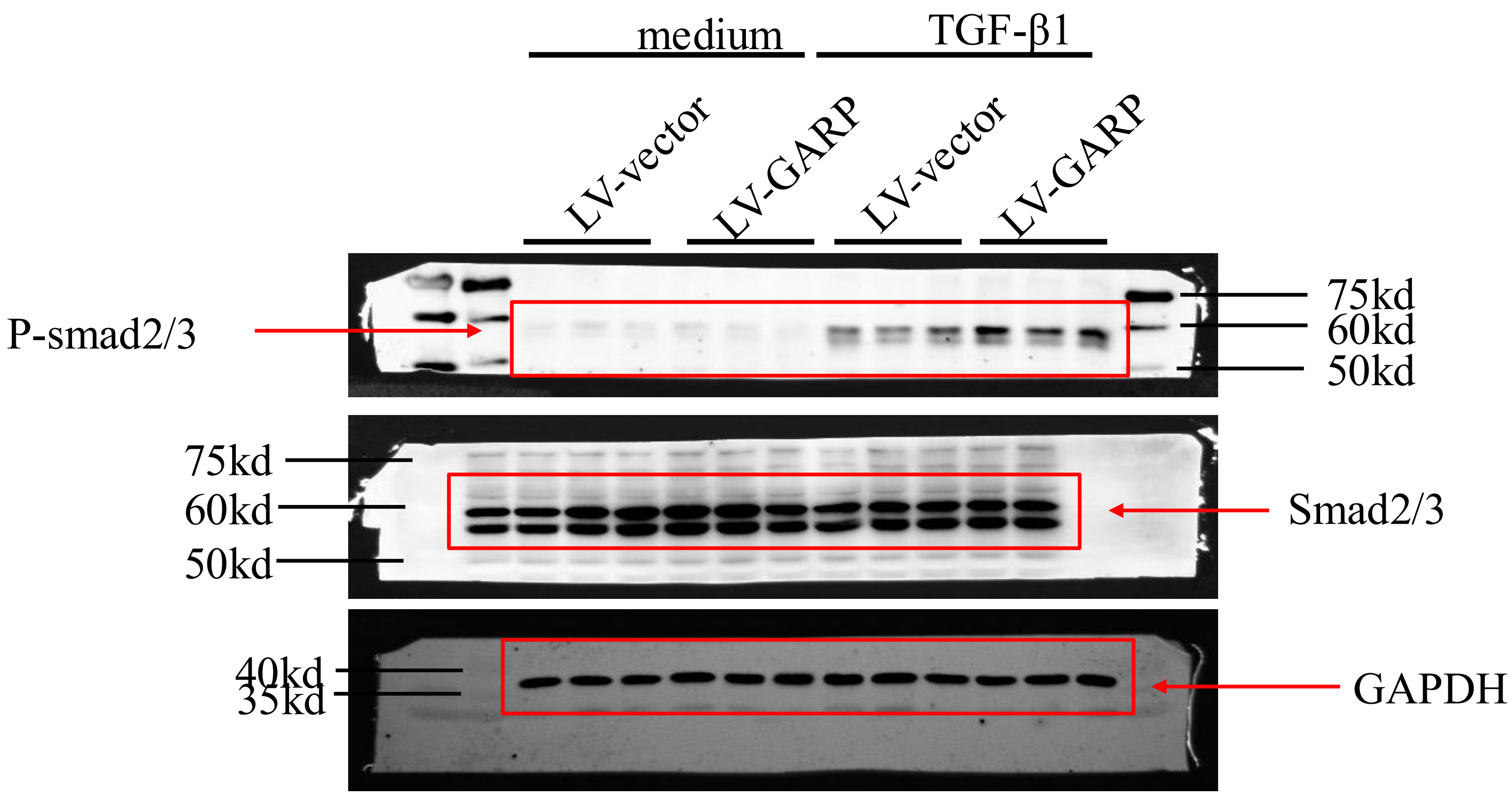
medium
TGF-β1
LV-vector
LV-GARP
LV-vector
LV-GARP
P-smad2/3
75kd
60kd
50kd
75kd
60kd
50kd
Smad2/3
40kd
35kd
GAPDH

Figure 5 M

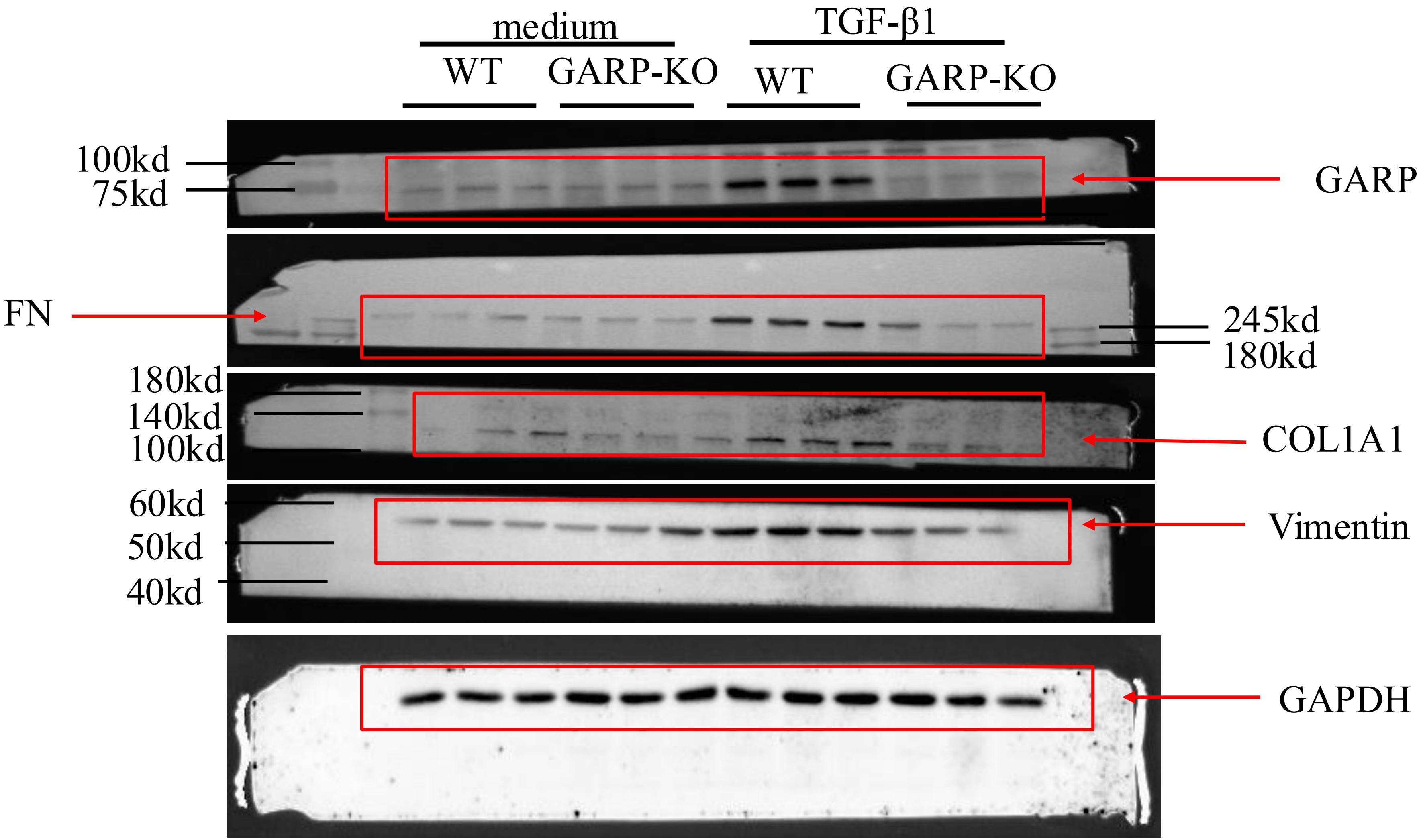
medium
TGF-β1
WT
GARP-KO
WT
GARP-KO
100kd
75kd
GARP
FN
245kd
180kd
180kd
140kd
100kd
COL1A1
60kd
50kd
40kd
Vimentin
GAPDH

Figure 5 O

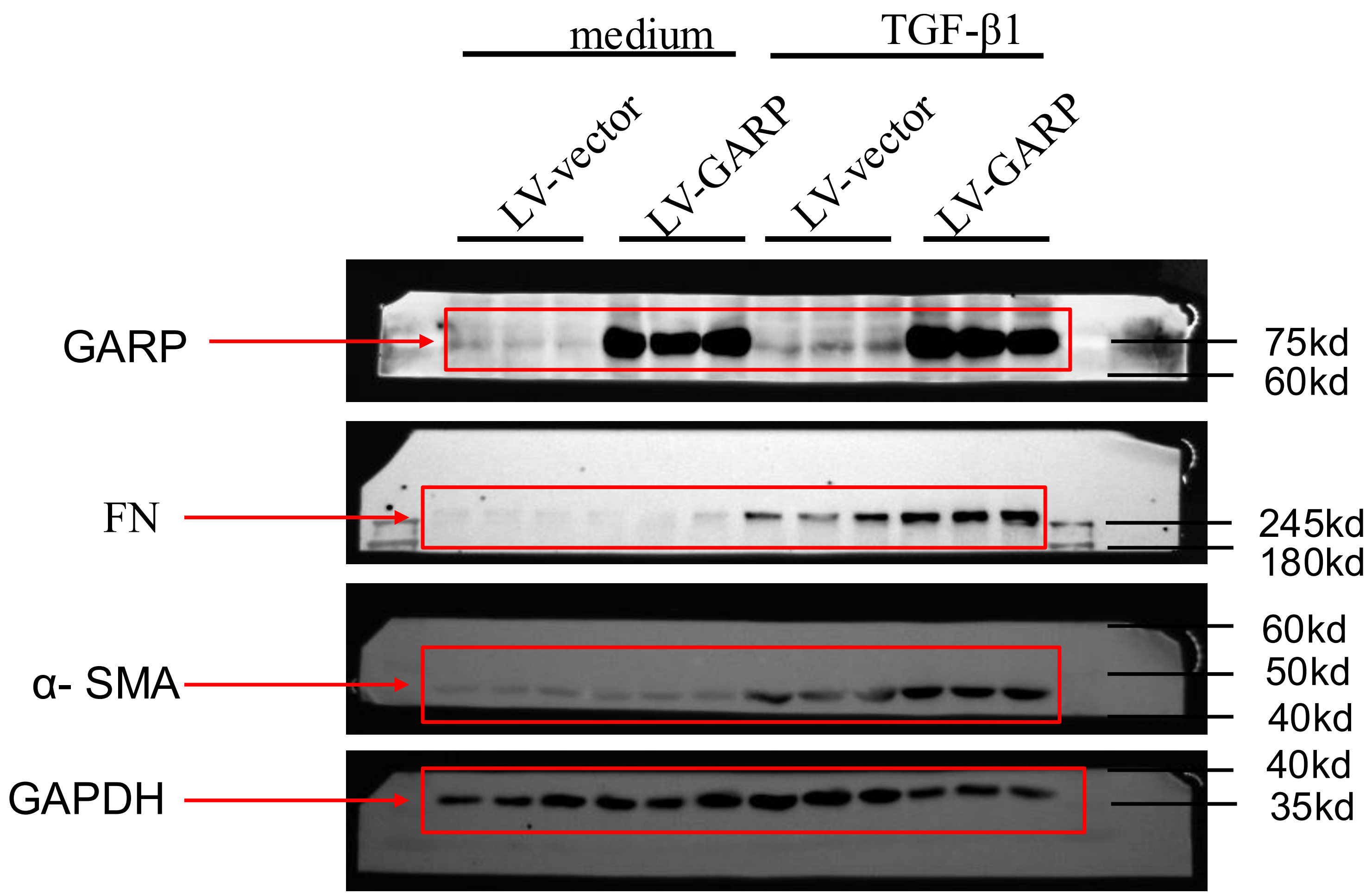

medium
TGF-β1
LV-vector
LV-GARP
LV-vector
LV-GARP
GARP
FN
α- SMA
GAPDH
75kd
60kd
245kd
180kd
60kd
50kd
40kd
40kd
35kd

Figure 6 D

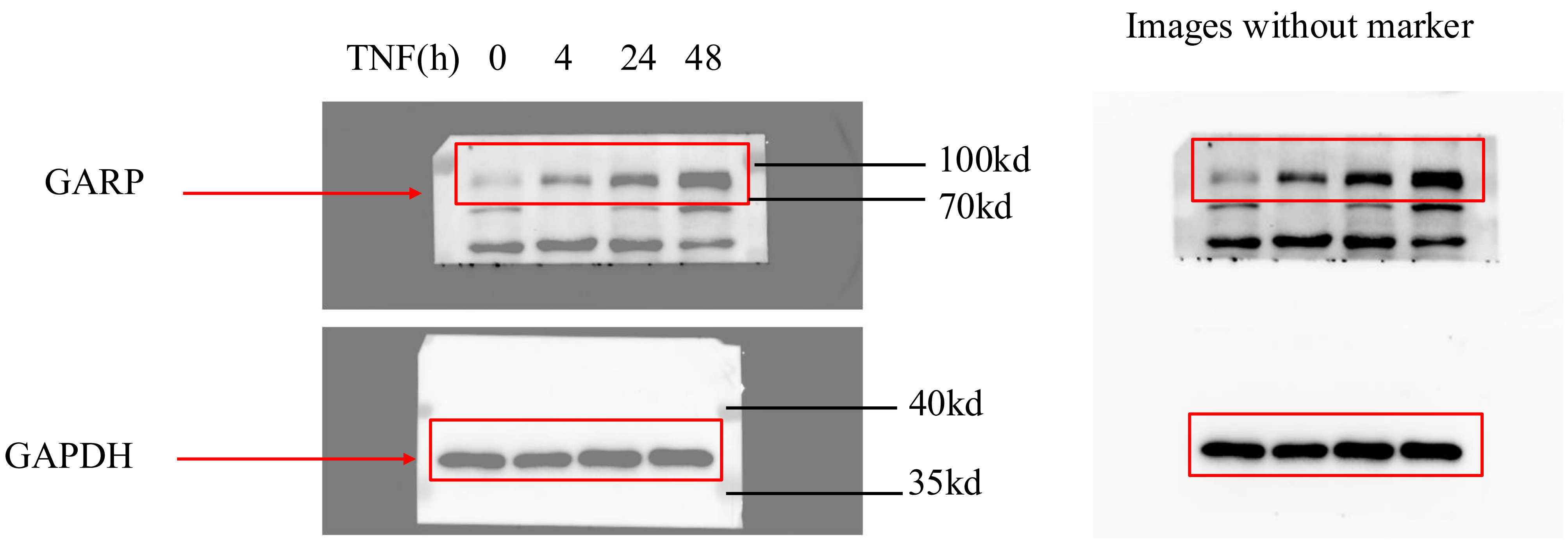
TNF(h) 0 4 24 48
GARP
GAPDH
100kd
70kd
40kd
35kd
Images without marker

Figure 6 F

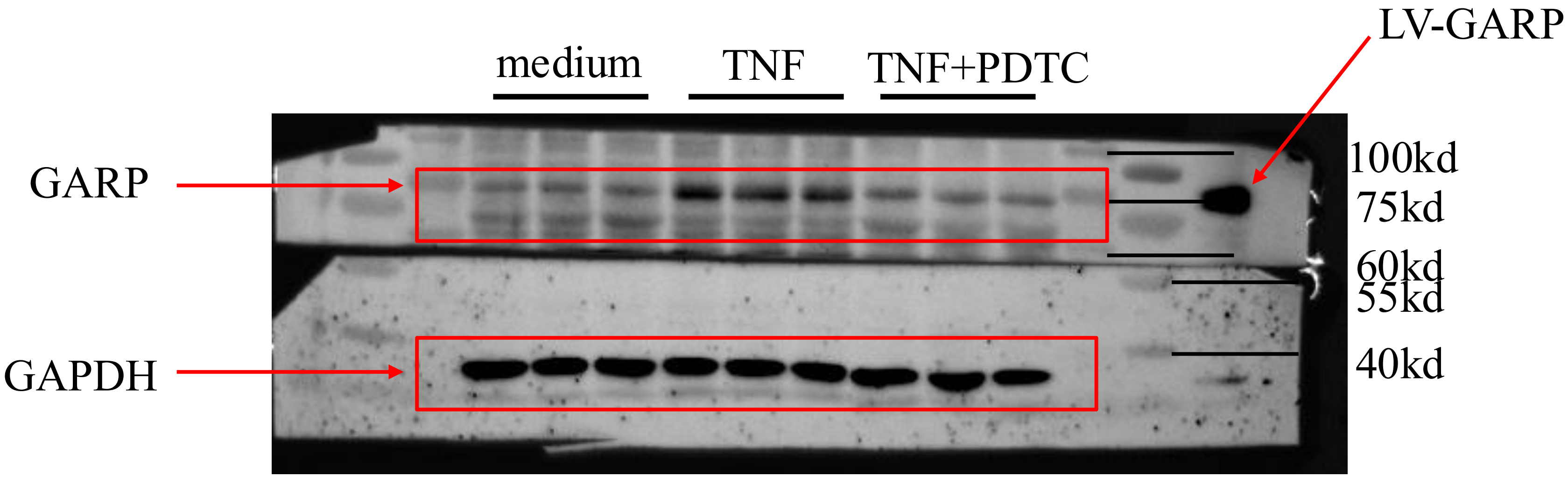
medium
TNF
TNF+PDTC
LV-GARP
GARP
GAPDH
100kd
75kd
60kd
55kd
40kd

Figure 6 K

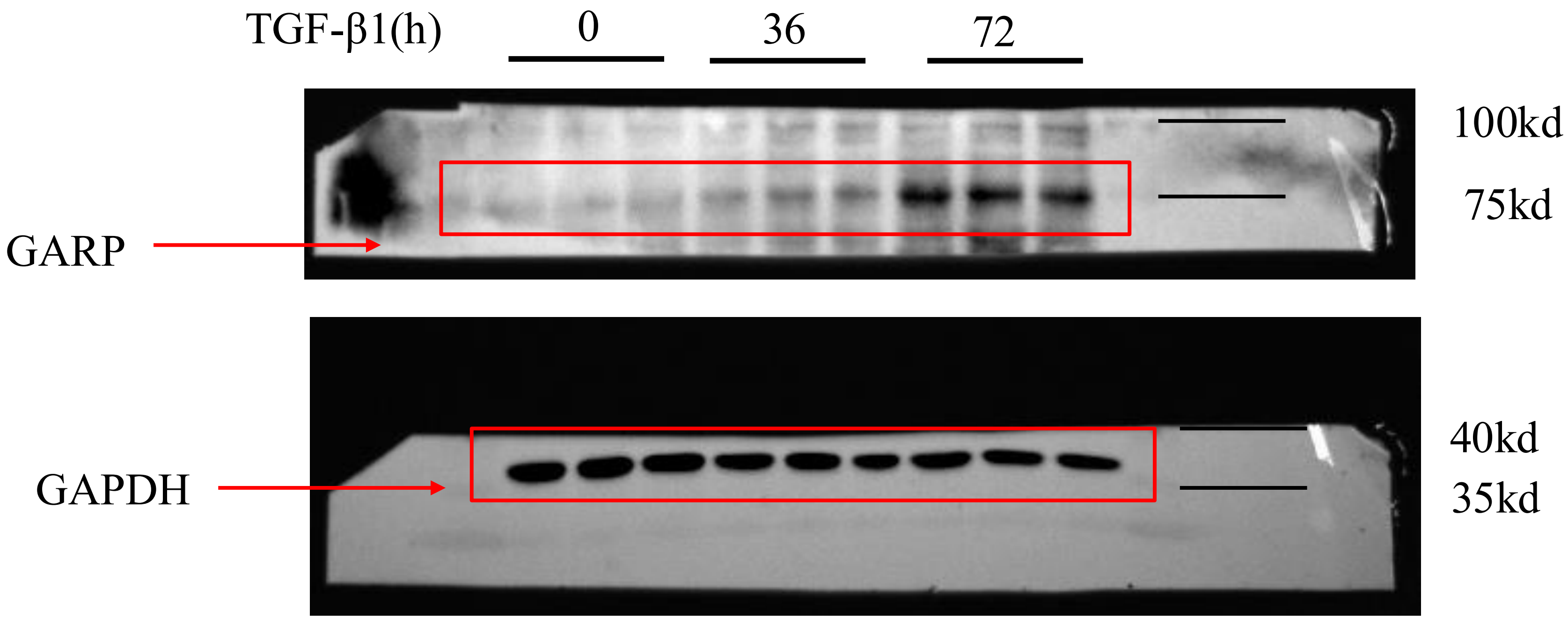

Figure 6 N

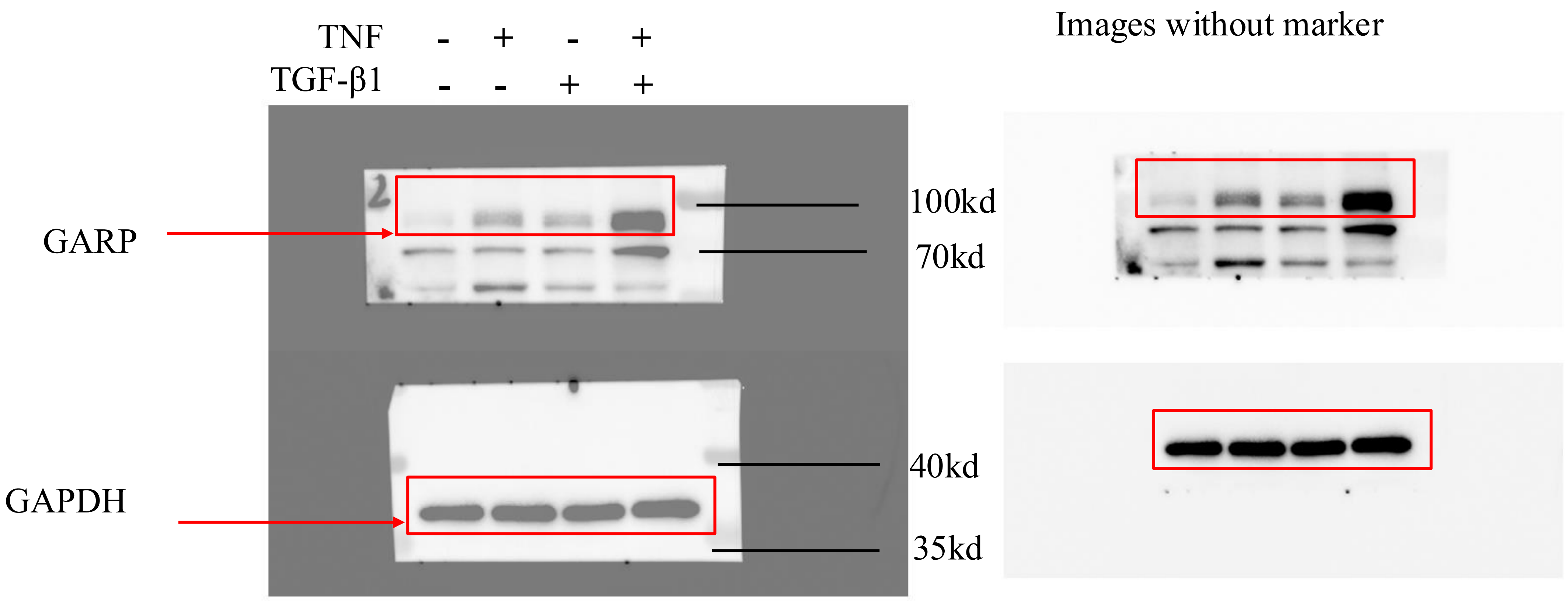
TNF - + - +
TGF-β1 - - + +
GARP
GAPDH
100kd
70kd
40kd
35kd
Images without marker

Figure 7 B

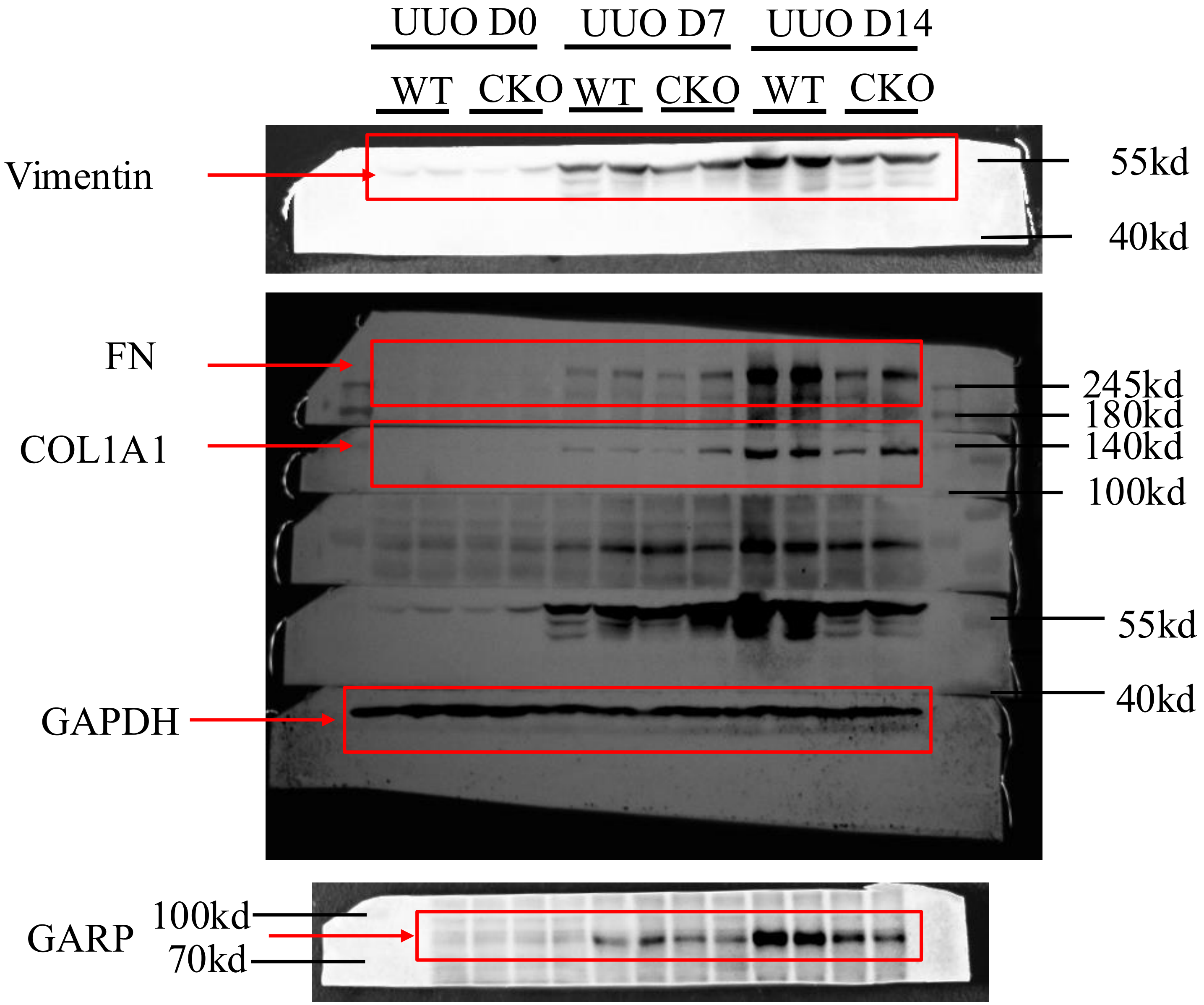
UUO D0
UUO D7
UUO D14
WT
CKO
WT
CKO
WT
CKO
Vimentin
55kd
40kd
FN
COL1A1
245kd
180kd
140kd
100kd
55kd
40kd
GAPDH
GARP
100kd
70kd

Supplemental Figure 1 B

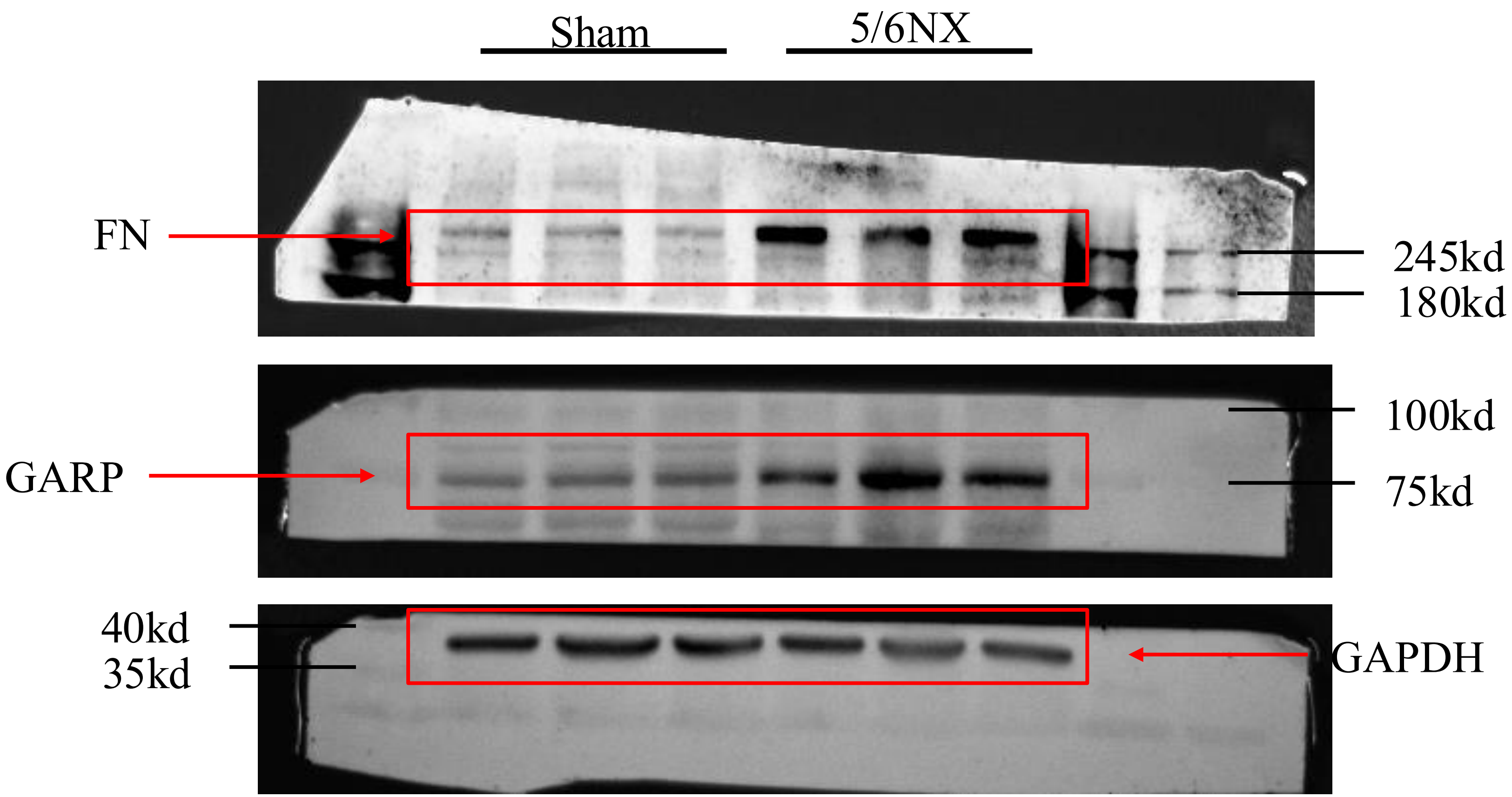

Supplemental Figure 1 E

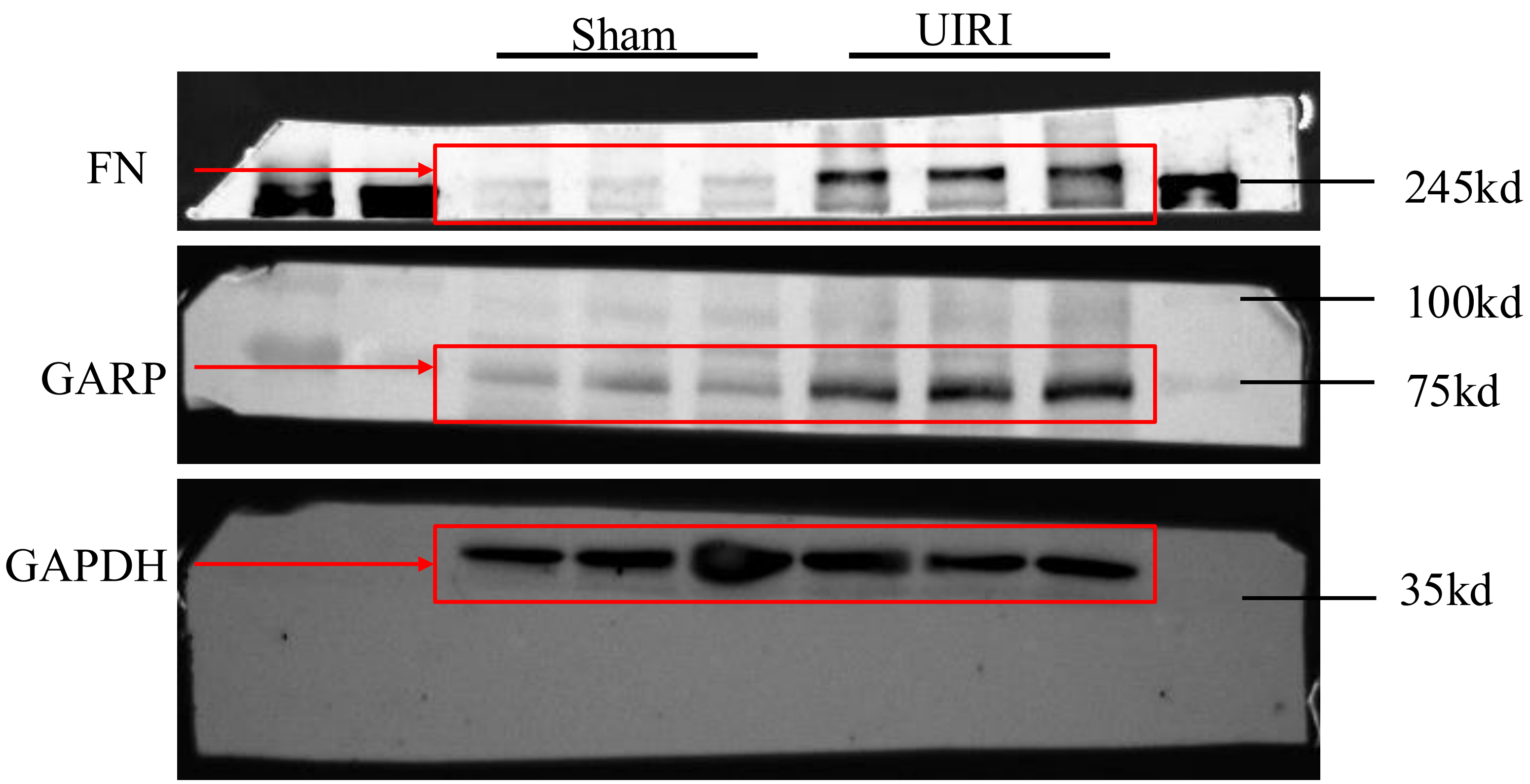

Supplemental Figure 2 B

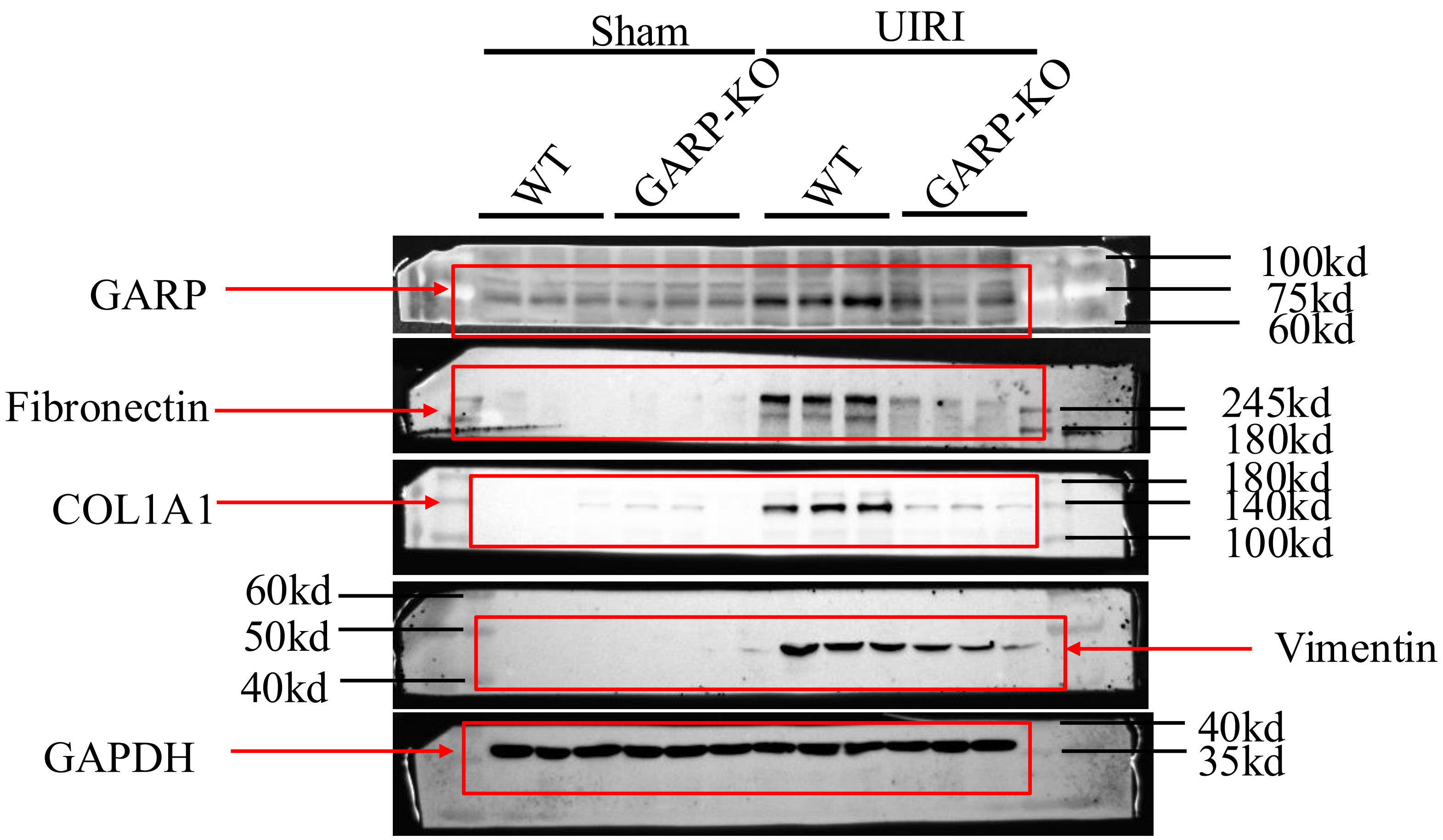

Supplemental Figure 6 A

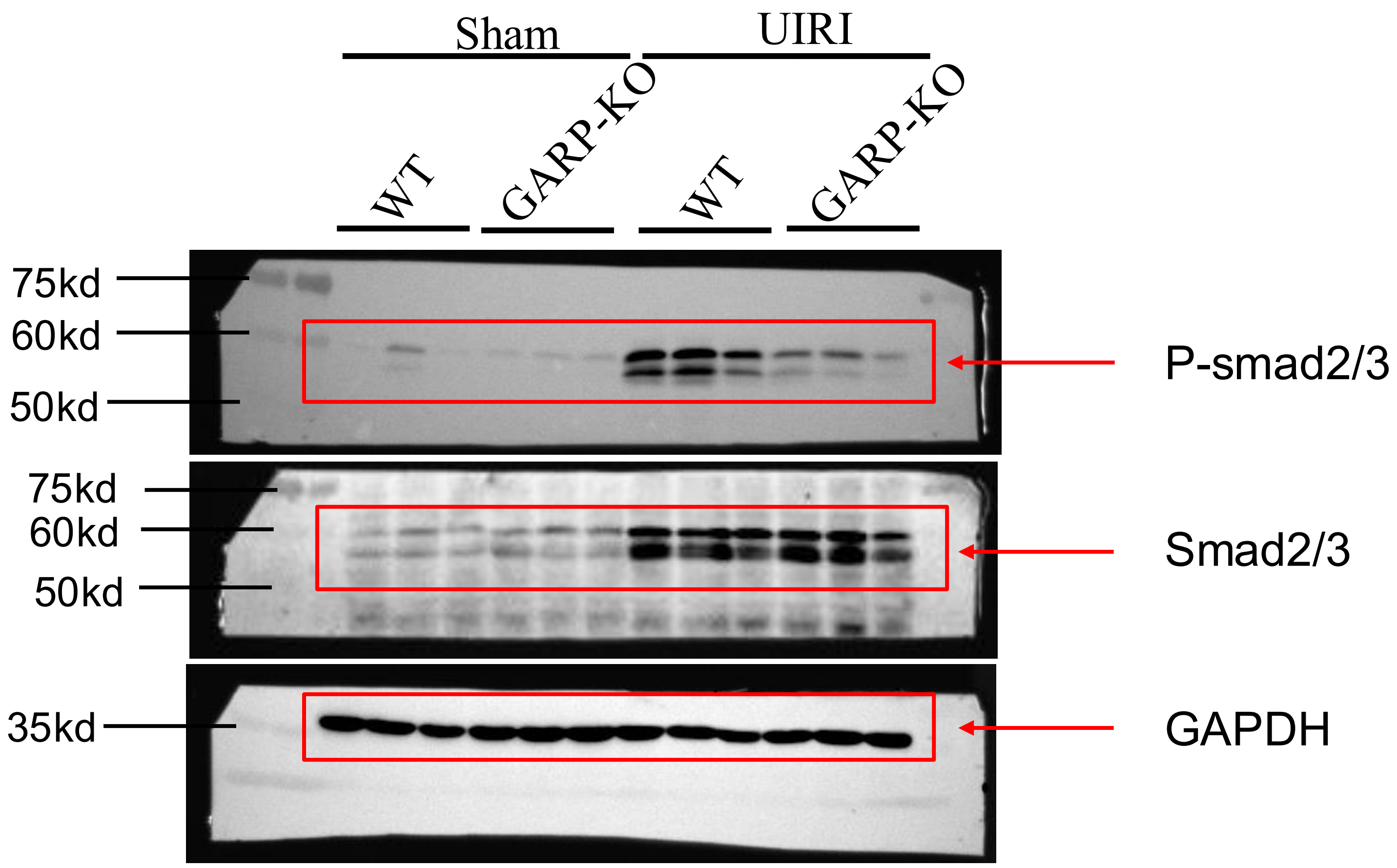

Supplemental Figure 7 C

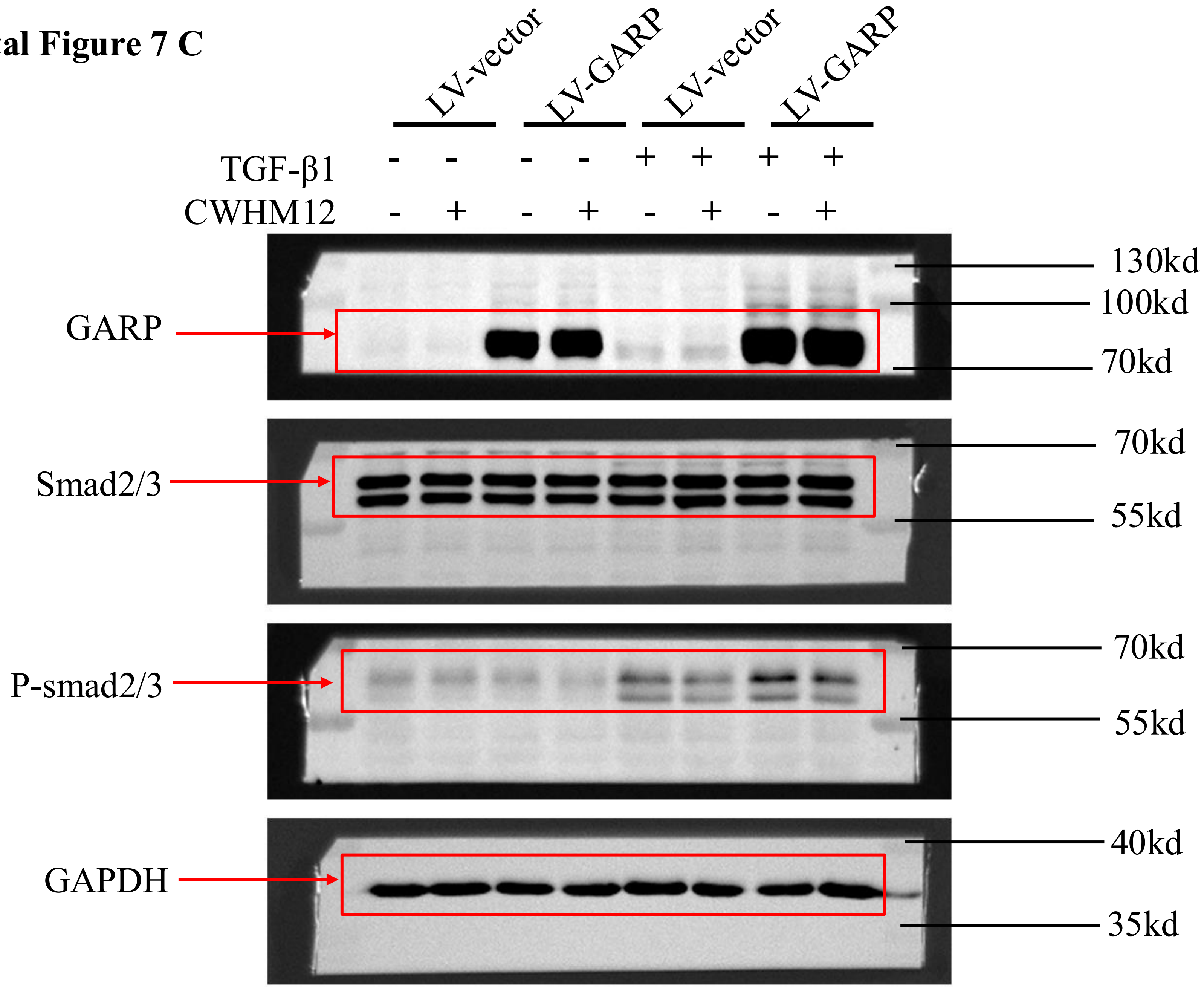

Supplemental Figure 7 D

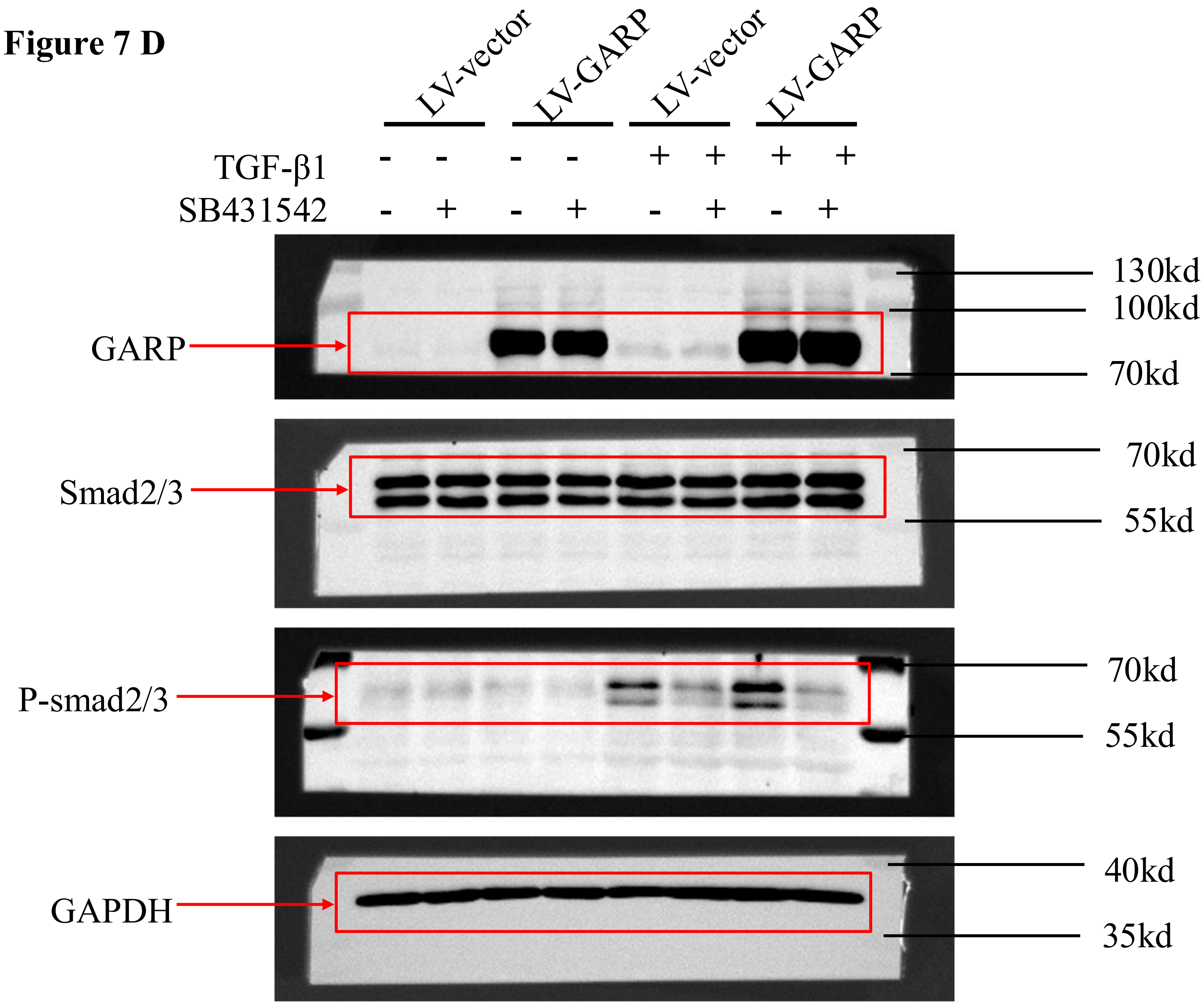

Supplemental Figure 8 B

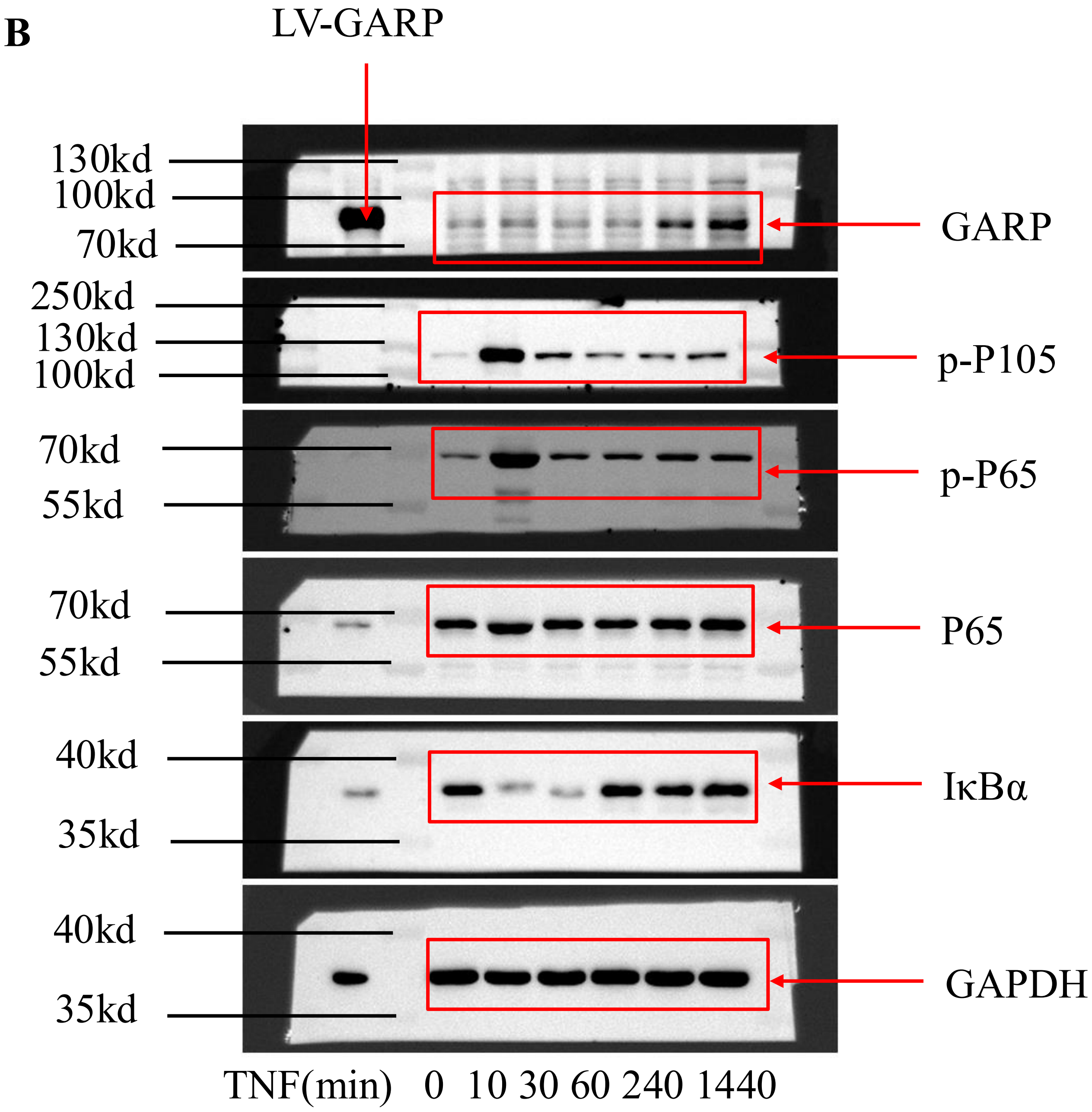

Supplemental Figure 9 B

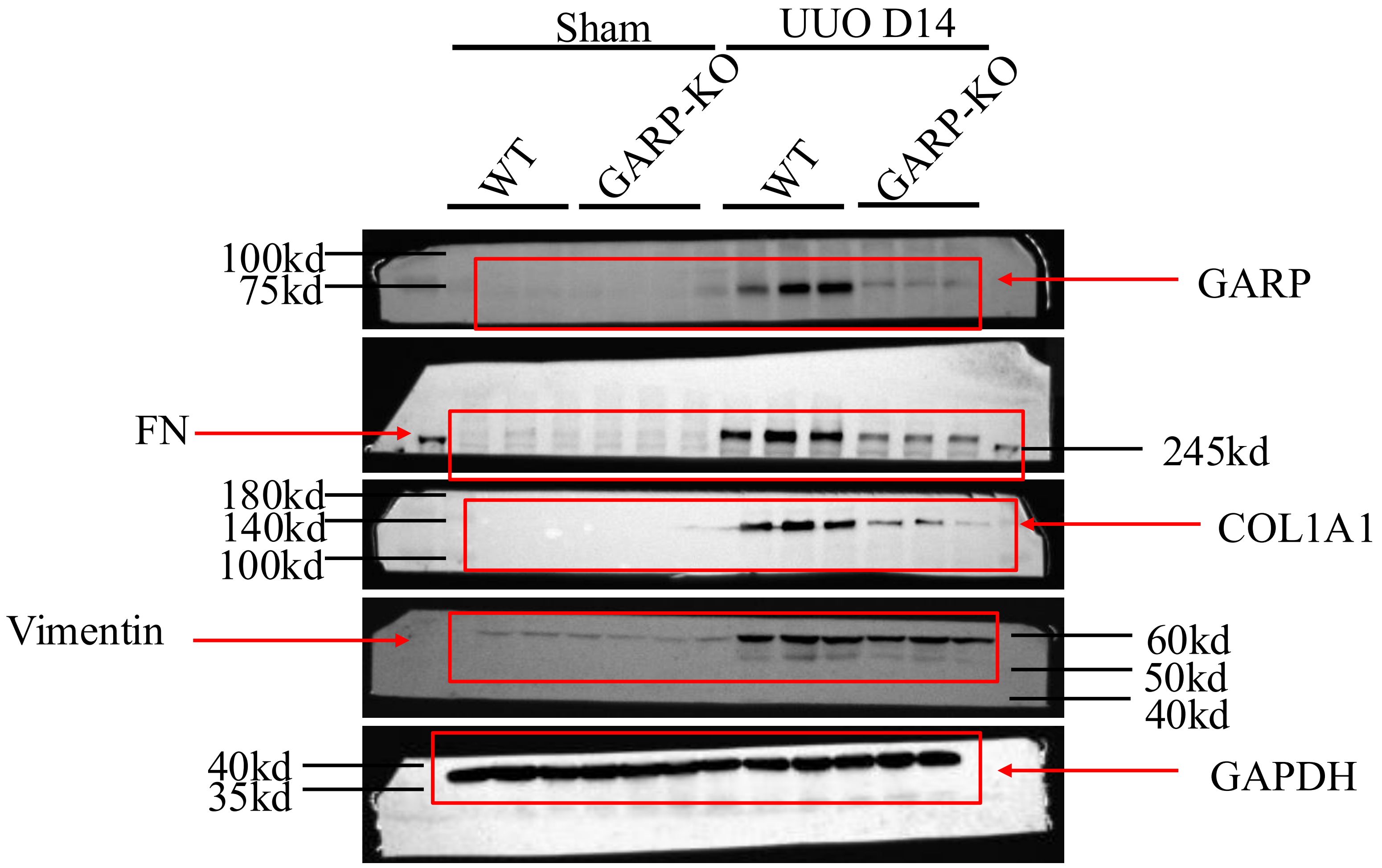

Supplemental Figure 9 F

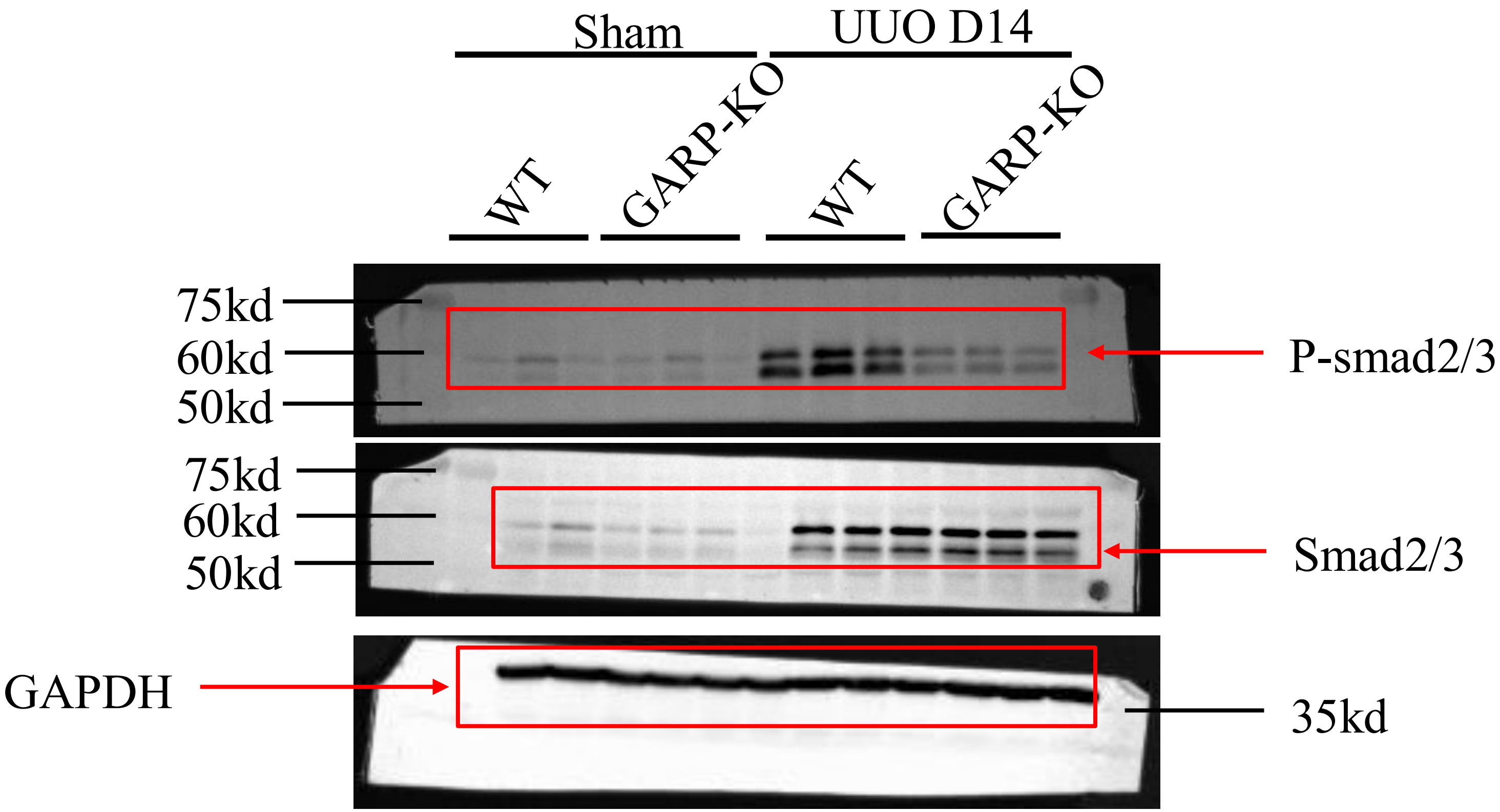

Supplement: Supplementary file 2 [file jciinsight-11-203039-s023.pdf]
